# Development of longitudinal datasets (2000–2020) with high spatiotemporal resolution for air pollution exposure assessment in Canada

**DOI:** 10.1016/j.dib.2025.111730

**Published:** 2025-05-30

**Authors:** Anas Alhusban, Yasar Burak Oztaner, Markey Johnson, Mastaneh Rezasefat, Negin Hojjatzadeh, Israa Moussa, Mahsa Soleimani, Saeed Nadi, Shunliu Zhao, Hwashin Shin, Joyce J.Y. Zhang, Amir Hakami

**Affiliations:** a Department of Civil and Environmental Engineering, Carleton University, Ottawa, Ontario, Canada; b Air Sectors Assessment and Exposure Science Division, Health Canada, Ottawa, Ontario, Canada; c Environmental Health Science and Research Bureau, Health Canada, Ottawa, Ontario, Canada

**Keywords:** Air quality modelling, CMAQ, Machine learning, Hybrid modelling, PM_2.5_, Ozone, Nitrogen dioxide

## Abstract

We developed datasets intended to aid and inform, health and epidemiological studies in Canada by providing highly resolved spatiotemporal concentrations for regulated air pollutants (fine particulate matter PM_2.5_, nitrogen dioxide NO_2_, and ozone O_3_) across Canada. Daily estimates were generated at various spatial resolutions for the years 2000 through 2020. The datasets are based on simulations of the US EPA’s Community Multiscale (CMAQ) model at 12 km horizontal resolution. In an effort to increase the accuracy and spatial resolution of the exposure estimates, especially in complex urban environments, we used statistical and machine learning methods to downscale CMAQ outputs to finer resolutions. Downscaling relies on raw CMAQ results, high-resolution land-use datasets, existing concentrations datasets, and observations from the National Air Pollution Surveillance (NAPS) network. Widely used machine learning (ML) algorithms like random forest and gradient boosting were chosen and proved to be promising. Generated datasets at various spatial resolutions (census divisions, postal codes, gridded 12 km, or 1 km) showed adequate statistical performance and clear representations of spatial features associated with pollutant emissions.

Specifications Table

**Table:** 

Subject	Earth & Environmental Sciences
Specific subject area	*Air pollution modelling: Modelling pollutants (NO_2_, O_3_, PM_2.5_) over Canada for the 21 years period (2000–2020) at various spatial resolution.*
Type of data	Tables (comma separated values CSV file format). Binary (Network Common Data Form (netCDF) file format).
Data collection	The data was generated using software tools with inputs from publicly available data sources.
Data source location	*Institution: Carleton University* *City/Town/Region: Ottawa, Ontario*
Data accessibility	Repository name: Federated Research Data Repository (FRDR) Data identification number:10.20383/103.01169 Direct URL to data: https://www.frdr-dfdr.ca/repo/dataset/375ad7af-db91-42a0-b05e-8896c972aa70
Related research article	*None.*

## 1 Value of the Data

• The main value of this dataset stems from the high spatiotemporal resolution across Canada and for the extended period spanning 2000–2020. To our knowledge, it’s the first publicly available longitudinal dataset for multiple pollutants with daily values and fine scale spatial resolution in Canada.
• The generated datasets are valuable inputs for studies to quantifying the population health burden of air pollution and its longitudinal trends, and for epidemiological studies that aim to characterize the concentration-response relationship for air pollution exposure.
• The datasets help to identify nonattainment of the Canadian Ambient Air Quality Standards (CAAQS) in areas not covered by NAPS.
• By the virtue of their high resolution, the datasets can be used in environmental justice applications and to evaluate inequalities in air pollution exposure.
• Furthermore, the datasets could be utilized to estimate environmental (ecosystem) impacts of air pollution the last two decades.
• The datasets could be used to aid in environmental policy and accountability analysis by providing the retrospective baseline for exposure and burden assessment.

## 2 Background

Air pollution is one of the main environmental hazards that affects population health. A global burden of disease study found that ambient PM_2.5_ alone causes 4.14 million deaths [1]. High-resolution spatiotemporal concentrations of air pollutants are needed to assess exposure in epidemiological studies [2], evaluate attainment with regard to CAAQS, conduct trend analysis and accountability studies, and assess exposure inequalities. However, NAPS monitoring stations do not provide enough spatial coverage, particularly for a country as vast as Canada. Auxiliary data such as those from remote sensing could be used but are limited in their ability to isolate surface concentrations for many pollutants of interest with respect to human health impacts (i.e., gaseous pollutants and PM components) or provide retrospective estimates. Furthermore, in Canada satellites coverage is prone to the effects of cloud and snow which pose quality and reliability issues [3,4], as well as limiting temporal coverage (i.e., satellite derived estimates of PM in Canada are limited to long term estimates). Spatial interpolation techniques can be used where there is a homogeneous distribution of monitoring network and smooth emission gradients; however, spatially interpolating monitoring data has been found to be insufficient on its own to estimate exposure [5].

Chemical Transport Models (CTMs), such as CMAQ, provide physics-based representation of atmospheric concentrations and full temporal coverage; however, they are often limited in their spatial resolution due to computational constraints. Hybrid modelling in the context of exposure estimation entails combining sources of information such as monitoring data, spatial interpolation, statistical models, and physics-based models to generate useful estimates of human exposure [6]. Hybrid models can increase the accuracy of exposure estimates in a complex urban environment [7], and help to better understand the role of exposure at the personal level [8].

## 3 Data Description

A summary of datasets, file names, and descriptions are detailed in Table 1 below, a detailed description of each dataset, visualisations and performance evaluations are found in Sections 3.1 to 3.4.

**Table 1:** Datasets and file names description.

S/N	Dataset Folder	File name	Description
1	CMAQ	CMAQ_Daily_20XX_CarletonU_v2.nc	Daily average surface 12-km by 12-km output files (includes all pollutants).
2	CMAQ	CMAQ_Annual_20XX_CarletonU_v2.nc	Annual average surface 12-km by 12-km output files (includes all pollutants).
3	CD	CD_AVG24H_${pollutant}_20XX_CarletonU_v2.csv	Daily average surface census division concentration
4	CD	CD_MAX1H_${pollutant}_20XX_CarletonU_v2.csv	Daily maximum hourly concentration surface (census divisions).
5	CD	CD_MAX3H_${pollutant}_20XX_CarletonU_v2.csv	Daily maximum 3 h moving average concentration surface (census divisions).
6	CD	CD_MAX8H_${pollutant}_20XX_CarletonU_v2.csv	Daily maximum 8 h moving average concentration surface (census divisions).
7	HYBRID	HYBRID_LUR_${pollutant}_20XX_CarletonU_v2.csv	Daily average surface at the postal code level.
8	ML	ML_RF_Daily_20XX_CarletonU_v1.nc	Daily average 1 km surface covering most of Canada produced using Random Forest (includes all pollutants).
9	ML	ML_HGB_Daily_20XX_CarletonU_v1.nc	Daily average 1-km surface covering most of Canada produced using Histogram Gradient Booster (includes all pollutants).
10	ML	ML_RF_Monthly_20XX_CarletonU_v1.nc	Monthly average 1-km surface covering most of Canada produced using Random Forest (includes all pollutants).
11	ML	ML_HGB_Monthly_20XX_CarletonU_v1.nc	Monthly average 1-km surface covering most of Canada produced using Histogram Gradient Booster (includes all pollutants).
12	ML	ML_RF_Annual_20XX_CarletonU_v1.nc	Annual average 1-km surface covering most of Canada produced using Random Forest (includes all pollutants).
13	ML	ML_HGB_Annual_20XX_CarletonU_v1.nc	Monthly average 1-km surface covering most of Canada produced using Histogram Gradient Booster (includes all pollutants).
14	All	ReadMe.txt	Detailed description and additional information

### 3.1 12-km CMAQ simulations

**Fig. 1:**
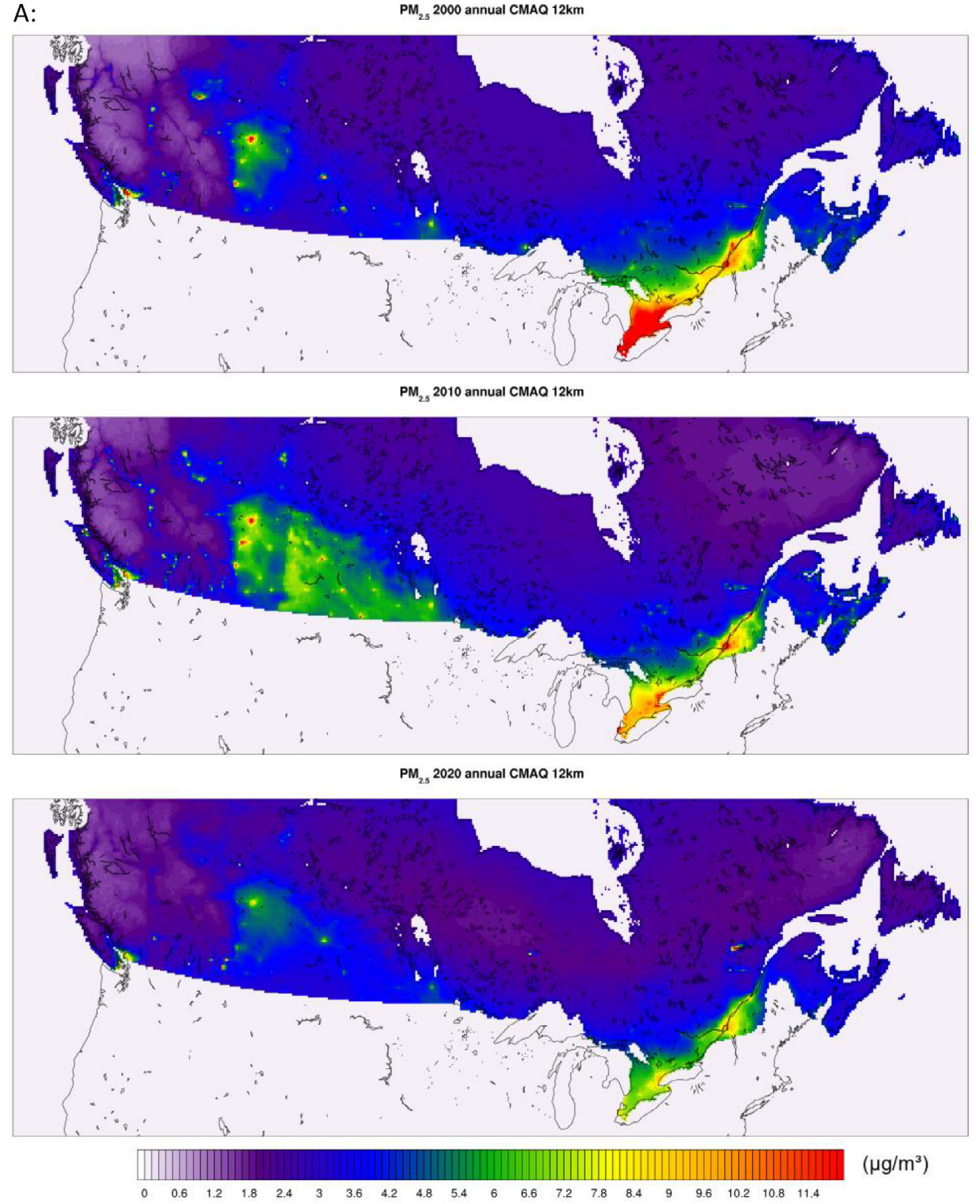

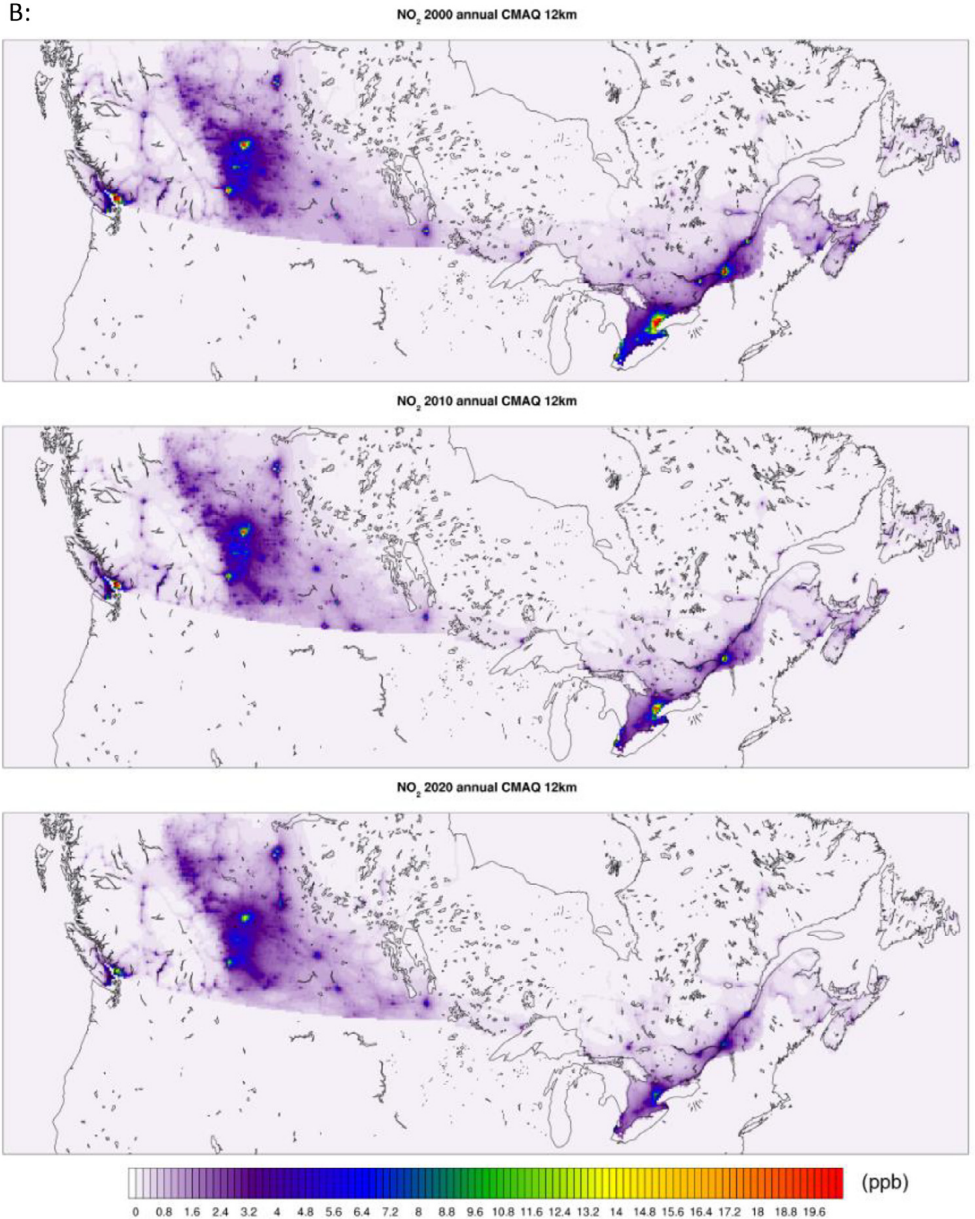

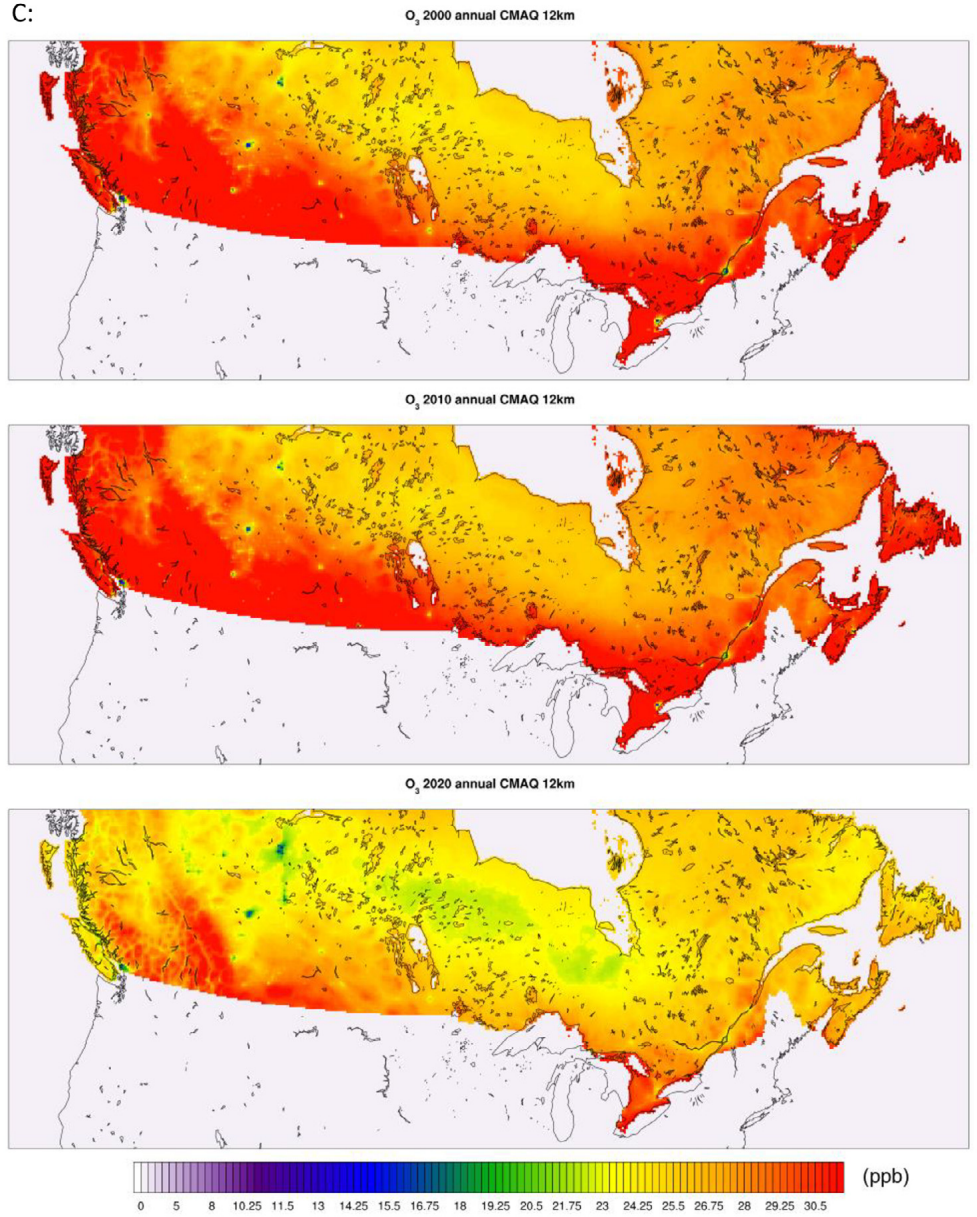
CMAQ annual average concentration surfaces (Canada) for the years 2000, 2010, and 2020 (a) PM_2.5_ in μg/m^3^ (b) NO_2_ in ppb (c) O_3_ in ppb.

All of the generated datasets are directly based on, or are derived from CMAQ simulations at 12-km resolution over North America. CMAQ raw outputs are in the netCDF4 file format. The files contain daily and annual averages for the pollutants PM_2.5,_ NO_2_, and O_3_ for the full period. CMAQ annual concentration plots for the sample years 2000, 2010, and 2020 featuring the pollutants PM_2.5_, NO_2_, and O_3_ are shown in Fig. 1 below. The statistical performance of base CMAQ runs is adequate across modelled years and for all pollutants as the domain wide correlation coefficient for daily estimates against NAPS for most years and pollutants above 0.5 (see Fig. 6 for comparative evaluation).

### 3.2 Census division datasets

**Fig. 2:**
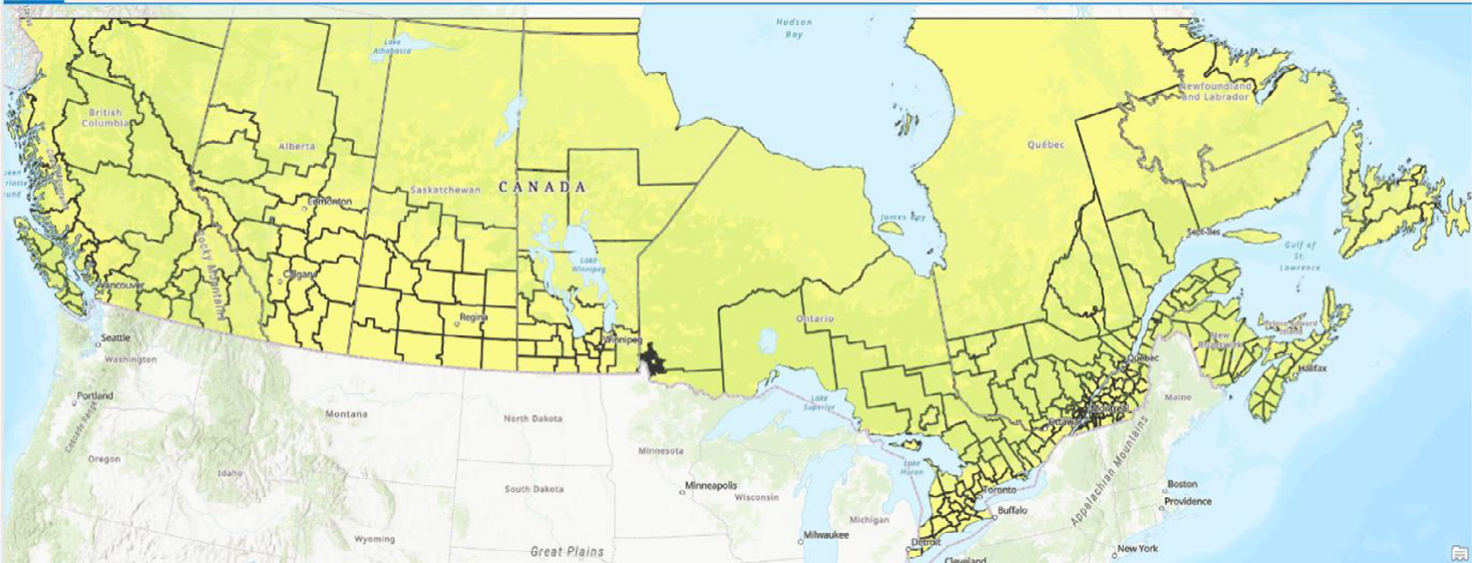
Census divisions contained in the datasets.

Census Division (CD) datasets are processed from CMAQ 12 km base hourly datasets and cover most of the 10 provinces encapsulating 99.6 % of the 2016 Canadian population as can be seen in Fig. 2 below. The CD datasets include the full duration (2000–2020) for the pollutants (PM_2.5_, NO_2_, and O_3_), and they are tabulated in CSV format by year and include daily average, one hour maximum, three hours maximum, and 8 hours maximum moving average concentrations. In these files the rows represent dates and the columns are CD identification numbers. A sample snippet for PM_2.5_ daily averages featuring few census divisions and the beginning of the year 2010 is shown in Table 2 as an example. The statistical performance of the aggregated CD level dataset is in the same neighborhood of the base CMAQ performance. The performance evaluation results for the 24-hours daily average are shown in Table 3.

**Table 2:** Census divisions file snippet from the PM_2.5_ daily average files in μg/m^3^.

Date	1001	1002	1003	1004	1005
**2010-01-01**	5.0628	1.6741	1.2412	1.9855	3.1946
**2010-01-02**	7.8040	5.4704	3.8020	2.7694	2.8502
**2010-01-03**	3.6142	3.0199	2.0488	1.6488	1.5084
**2010-01-04**	2.1857	1.7466	0.8613	1.7133	1.8074
**2010-01-05**	3.5353	2.4086	1.5609	1.6809	2.6048
**2010-01-06**	6.1675	2.7244	2.0442	2.1828	2.2998
**……..**	……	……	……	……	……

**Table 3:** CD level 24-h average performance evaluation for PM_2.5_, NO_2_, and O_3_.

Year	PM_2.5_		NO_2_		O_3_	
	R	MB (µg/m^3^)	R	MB (ppb)	R	MB (ppb)
**2000**	0.66	1.42	0.73	−4.00	0.59	5.23
**2001**	0.74	1.52	0.73	−3.79	0.66	4.01
**2002**	0.69	1.35	0.68	−3.92	0.60	3.55
**2003**	0.75	1.29	0.73	−3.63	0.60	3.26
**2004**	0.80	1.15	0.71	−3.04	0.54	4.32
**2005**	0.82	0.87	0.71	−2.87	0.63	3.72
**2006**	0.75	0.83	0.69	−2.57	0.59	3.42
**2007**	0.77	0.86	0.69	−2.52	0.60	3.32
**2008**	0.76	1.10	0.69	−2.15	0.52	2.15
**2009**	0.69	0.90	0.67	−2.22	0.47	2.90
**2010**	0.62	0.53	0.69	−1.95	0.57	2.90
**2011**	0.71	1.08	0.67	−1.85	0.50	-1.72
**2012**	0.70	0.42	0.68	−1.46	0.56	3.04
**2013**	0.76	0.54	0.65	−1.62	0.51	-0.72
**2014**	0.73	0.03	0.65	−1.83	0.43	2.06
**2015**	0.61	1.77	0.65	−1.45	0.39	2.61
**2016**	0.61	1.32	0.66	−1.45	0.38	2.77
**2017**	0.60	1.28	0.65	−1.22	0.32	1.98
**2018**	0.69	-0.89	0.74	−1.87	0.62	-0.56
**2019**	0.62	0.00	0.74	−1.44	0.67	-0.03
**2020**	0.54	-0.70	0.72	−1.63	0.65	-0.42

### 3.3 CMAQ-LUR hybrid datasets

CMAQ datasets are downscaled to 6-digit postal codes using existing Land Use Regression (LUR) models as hybrid datasets that are based on CMAQ daily averages and annual datasets. These high-resolution datasets span the full 21 years; however, it is only limited to PM_2.5_ and NO_2_ as no high-resolution LUR exists for ozone. The datasets are presented as yearly CSV tables including daily concentrations for each postal code available for that year, where the rows represent the postal code and columns are the dates in the Julian format. Table 4 shows a snippet of the NO_2_ CSV file for the year 2020. The statistical performance of the CMAQ-LUR hybrid datasets versus the observations is matching the base CMAQ performance for PM_2.5_ but is slightly lower for NO_2_. (refer to Fig. 6 that shows the inter-dataset comparison).

**Table 4:** CMAQ-LUR hybrid datasets file snippet for 2020 NO_2_ in ppb.

POSTAL CODE	2020001	2020002	2020003	2020004	2020005	2020006	2020007	2020008
**A0A1A0**	0.145	0.691	0.264	0.694	0.284	0.804	0.931	0.219
**A0A1B0**	2.657	1.031	0.844	2.534	2.649	2.961	4.015	0.838
**A0A1C0**	0.093	0.342	0.312	0.889	0.338	1.072	1.214	0.264
**A0A1E0**	0.324	2.024	1.831	0.563	0.283	0.220	0.679	2.000
**A0A1G0**	3.383	3.642	3.018	3.698	3.432	2.539	5.753	5.008
**A0A1H0**	0.573	0.853	0.361	0.615	0.973	0.112	1.016	2.265
**A0A1J0**	1.361	4.575	3.624	5.367	2.420	2.046	9.606	5.627
**….**	…..	…..	…..	…..	…..	…..	…..	…..

### 3.4 Machine learning (ML) hybrid datasets

**Fig. 3:**
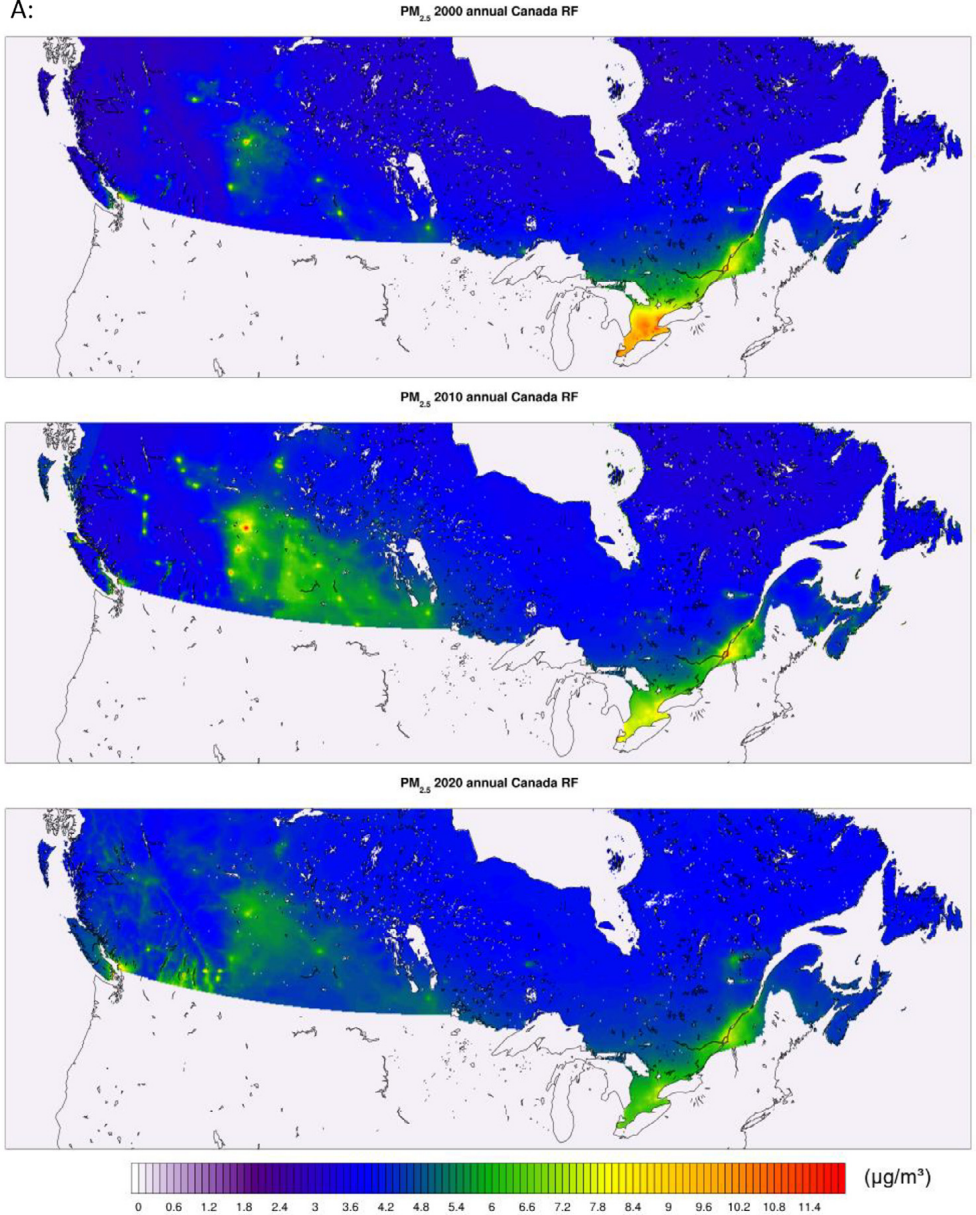

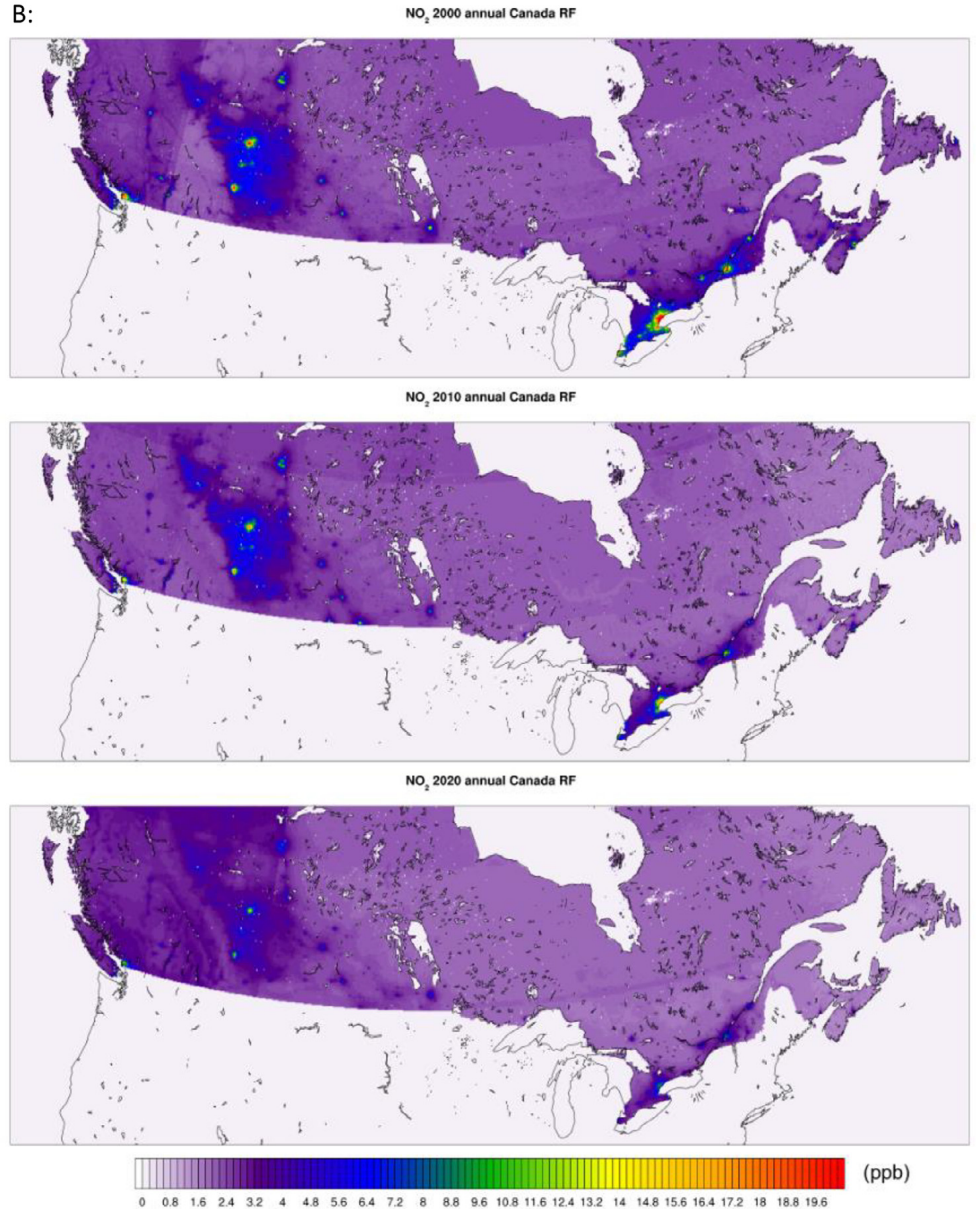

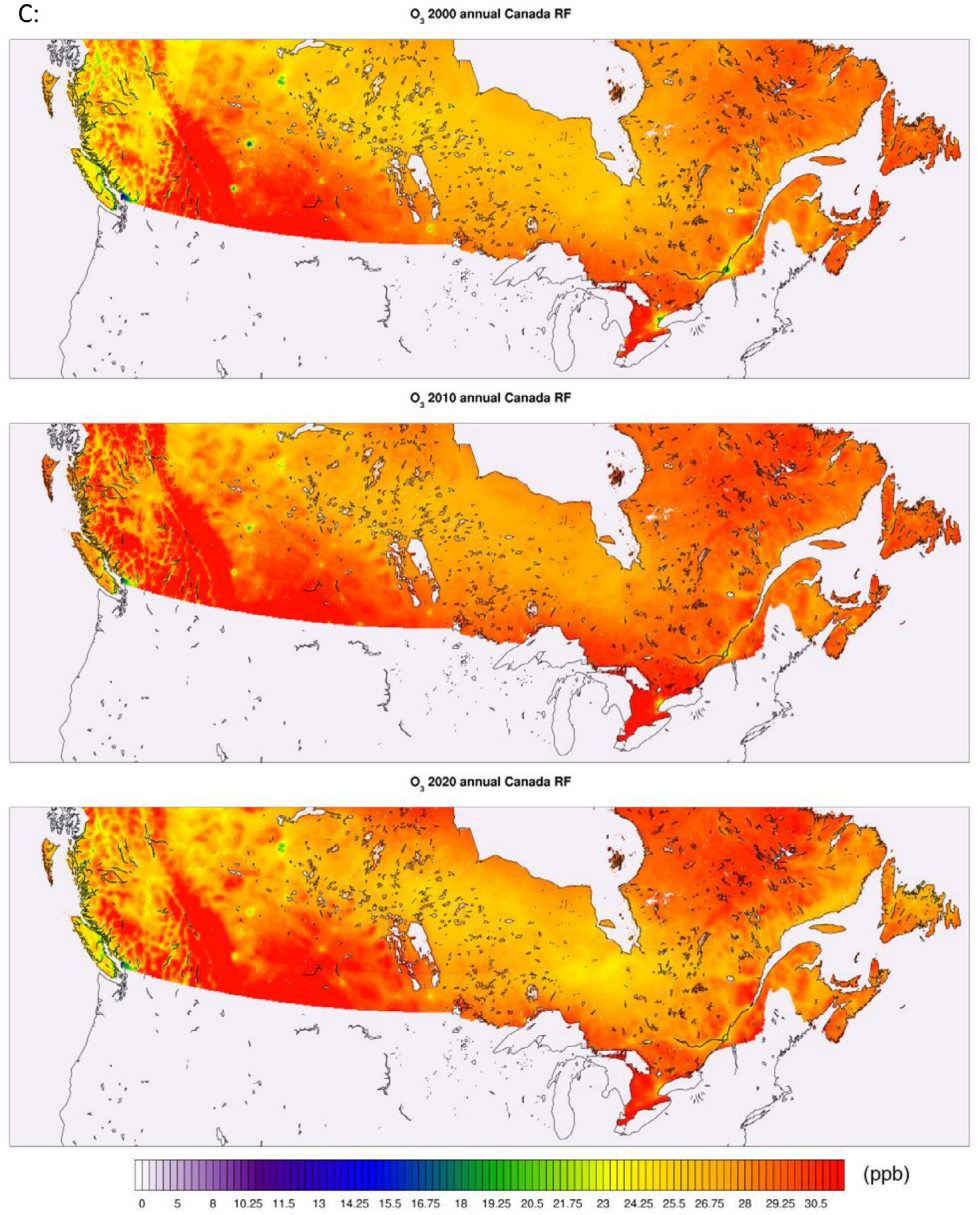
RF annual average concentration surfaces (Canada) for the sample years 2000, 2010, and 2020 (a) PM_2.5_ in µg/m^3^ (b) NO_2_ in ppb (c) O_3_ in ppb.

**Fig. 4:**
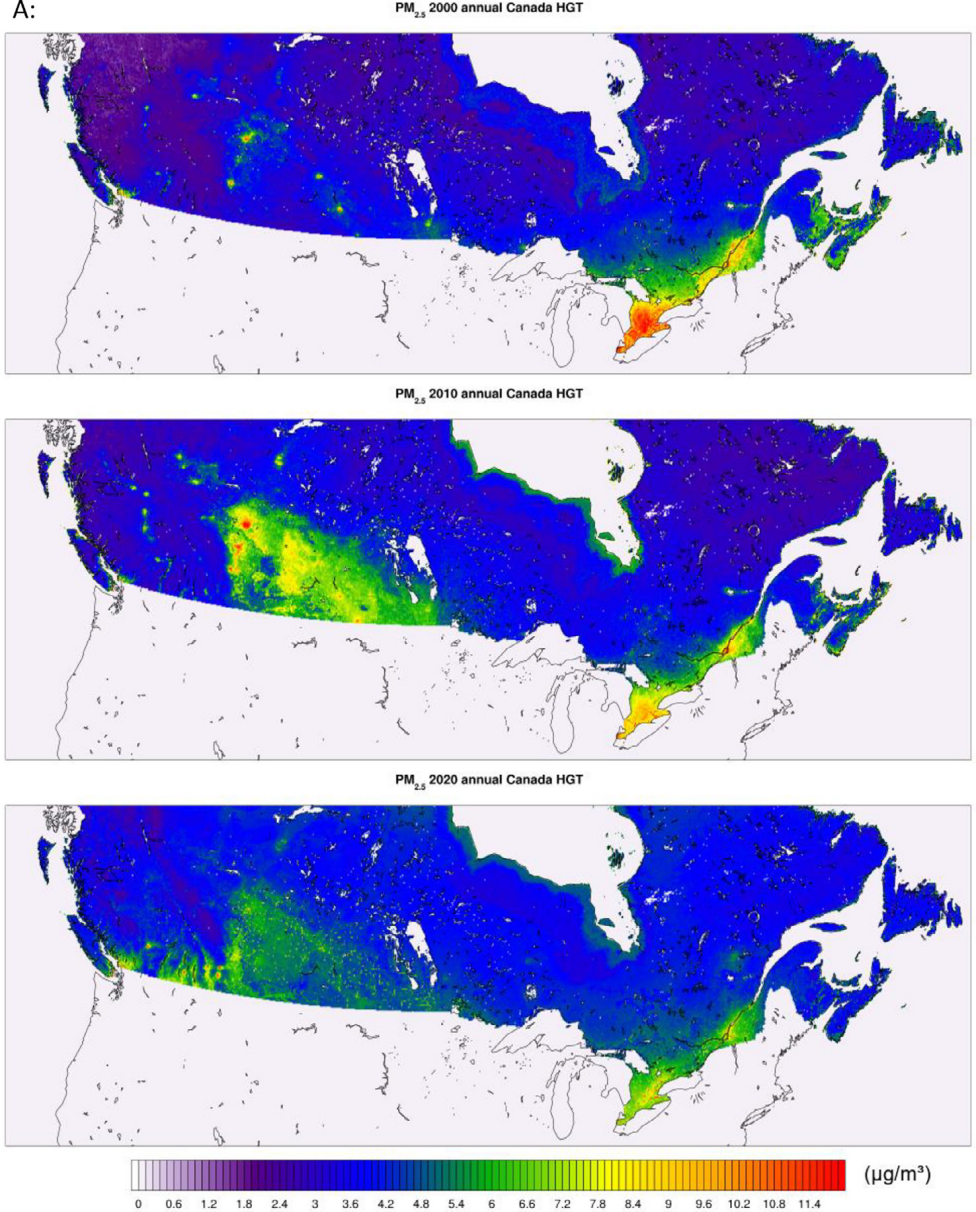

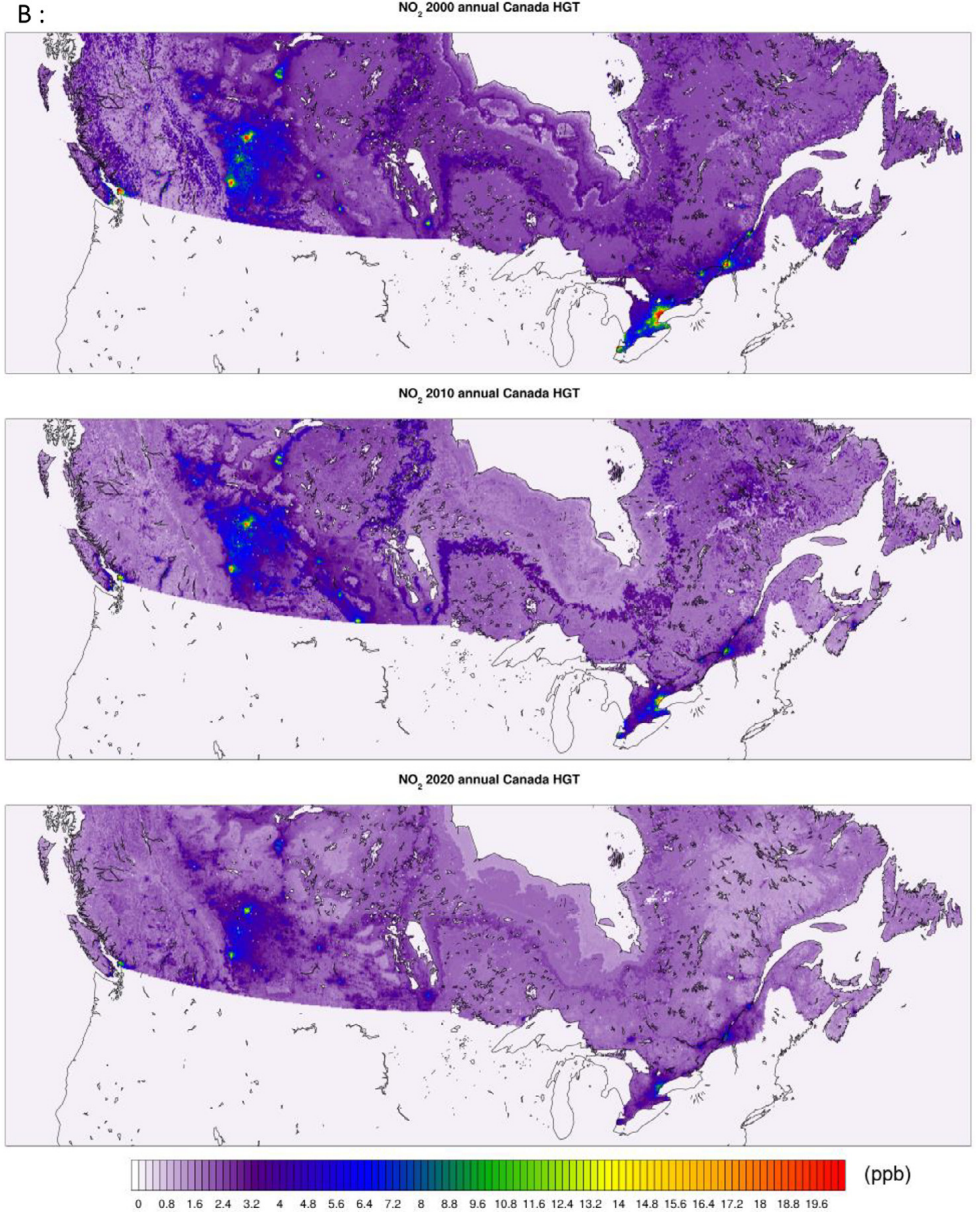

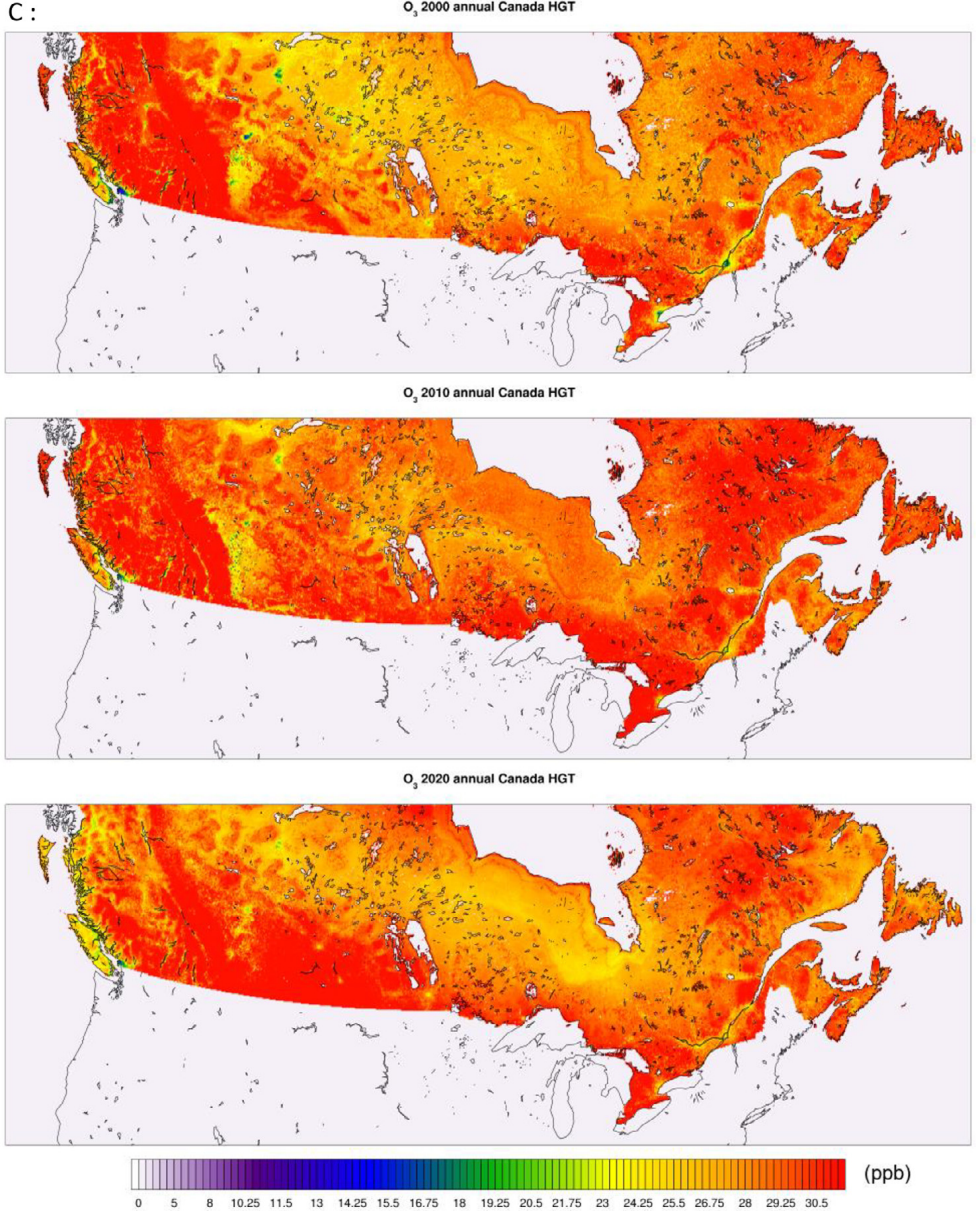
HGB annual average concentration surfaces (Canada) for the sample years 2000, 2010, and 2020 (a) PM_2.5_ in µg/m^3^ (b) NO_2_ in ppb (c) O_3_ in ppb.

**Fig. 5:**
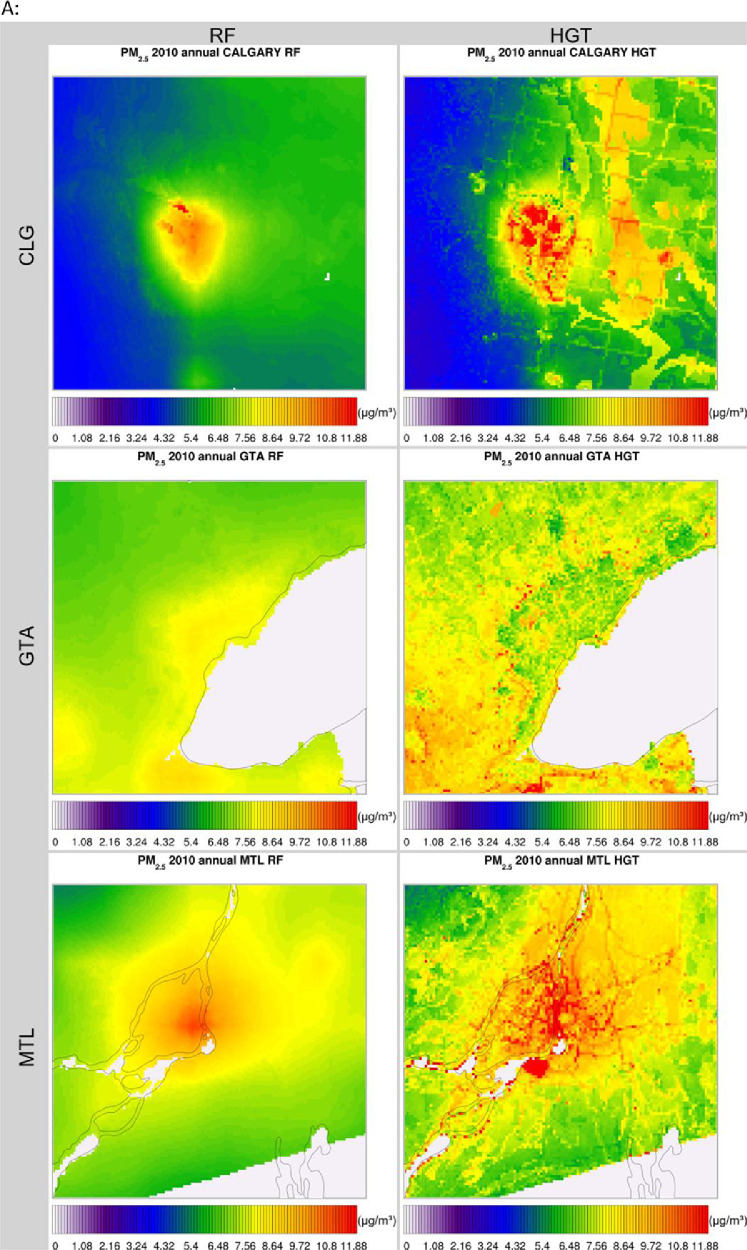

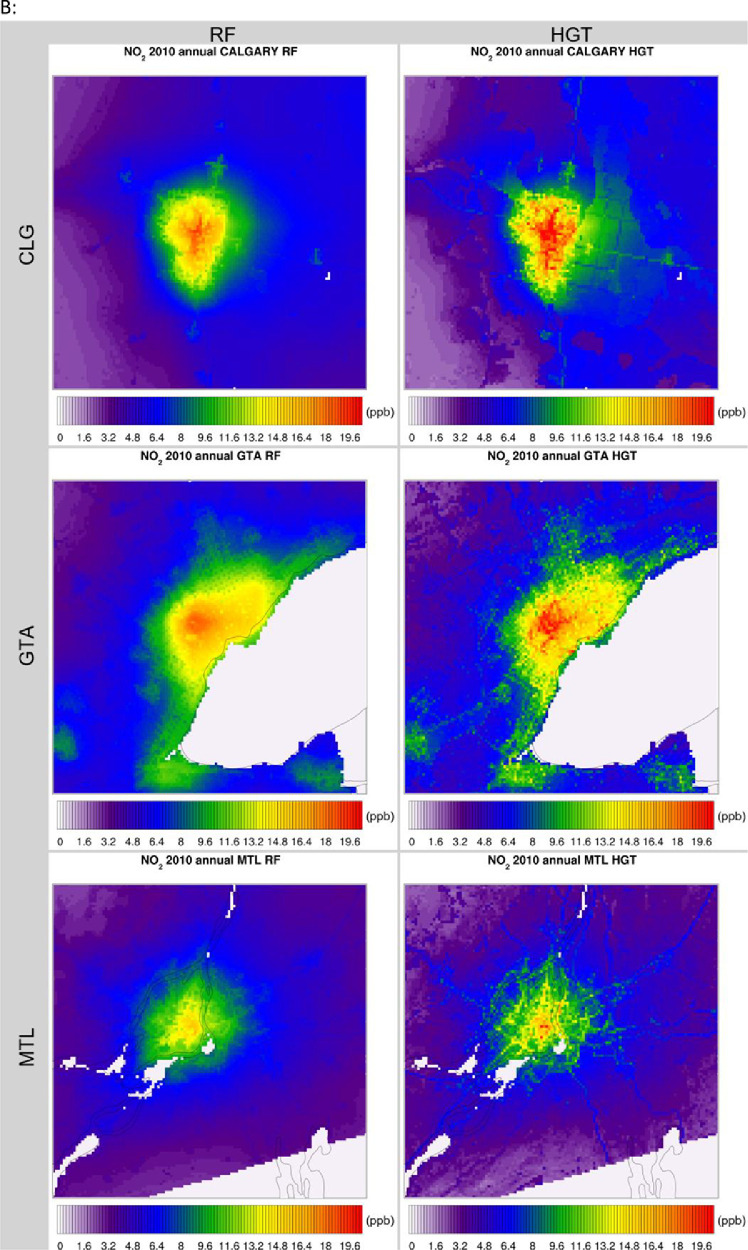

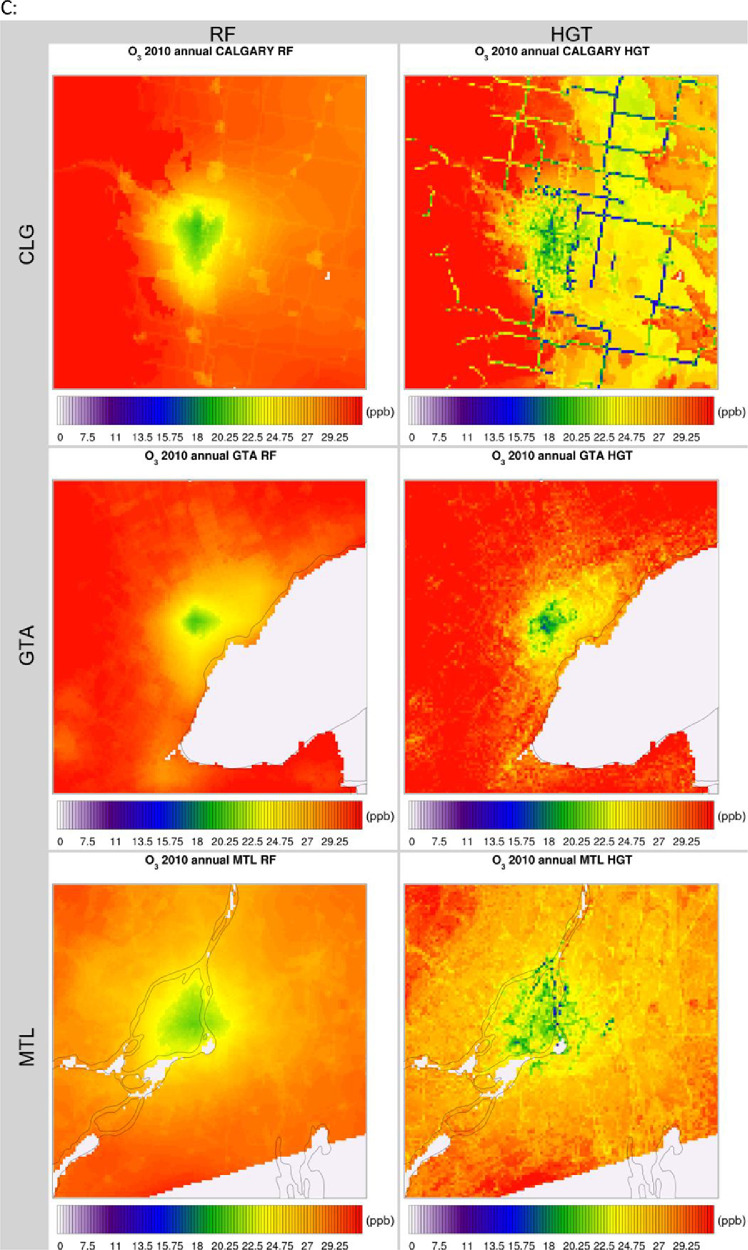
RF & HGB annual average concentration surfaces (Calgary, Toronto, Montreal) for the sample year 2010 (a) PM_2.5_ in µg/m^3^ (b) NO_2_ in ppb (c) O_3_ in ppb.

**Fig. 6:**
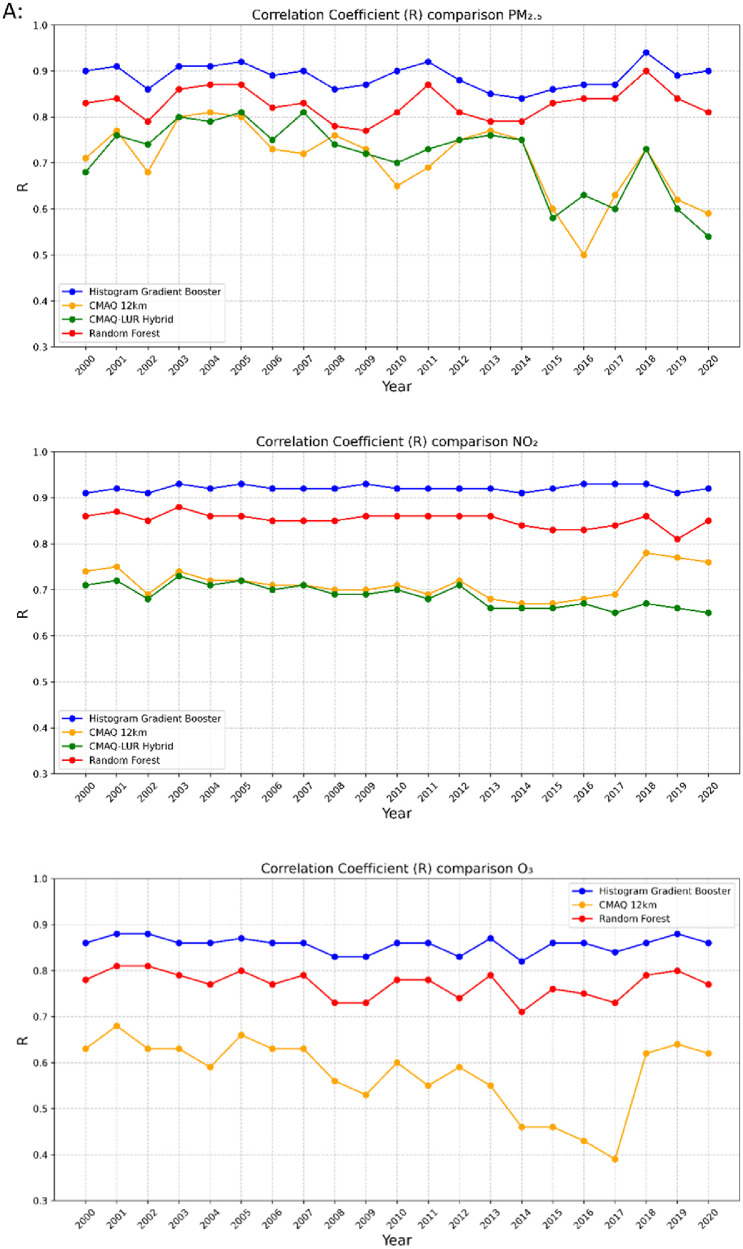

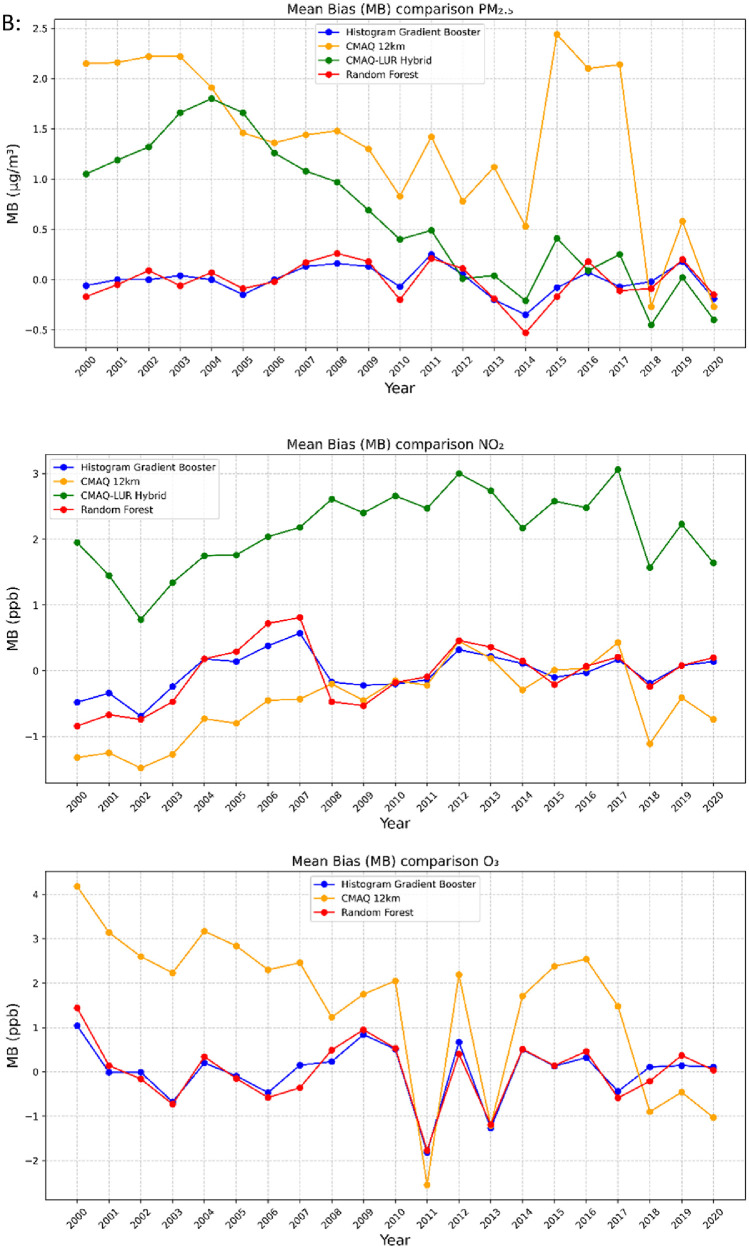
Comparative statistical performance evaluation of models CMAQ-12km, CMAQ-LUR Hybrid, RF, and HGB for PM_2.5_, NO_2_, O_3_ (a) correlation coefficients (b) mean bias.

The high-resolution 1-km by 1-km concentration surfaces were produced using two supervised machine learning algorithms in random forest (RF) and histogram gradient booster (HGB). The gridded outputs are produced in netCDF4 format and available for Canada. Sample concentrations produced with RF and HGB for Canada are shown below in Fig. 3, Fig. 4 respectively, while concentrations for the three cities of Toronto, Montreal, and Calgary appear in Fig. 5. RF produces smooth concentration surfaces that generally follow the CMAQ’s spatial patterns. Conversely, HGB produces heterogenous and location dependant concentrations, especially in urban areas where a stark contrast in land-use and emissions gradients exists. All datasets perform well against NAPS, a comparative performance evaluation of base CMAQ runs and both machine learning methods can be found in Fig. 6 below. The statistical performance evaluation includes correlation coefficient (R) and mean bias (MB). The test set phase wise statistical performance is shown in Table A1 in the appendix. Generally, HGB outperforms RF, while both ML models outperform base CMAQ and hybrid models for all pollutants. Both models performed well for most geographical regions for PM_2.5_, while the Southern Atlantic and the Rockies did not perform as well for O_3_ and NO_2_. Comparative performance with individual NAPS stations can be seen in Figures A1 (R) and A2 (MB) in the appendix.

## 4 Experimental Design, Materials and Methods

A summary of all the publicly available models, tools, and input data that were used in CMAQ modelling and further processing can be found in Table 5 below.

**Table 5:** Public input data, tools, and models used in the generation of concentration datasets.

Model/Input	Provider	Type of Data	Webpage
WRF	University Corporation for Atmospheric Research	Fortran source code	https://www2.mmm.ucar.edu/wrf/users/download/get_source.html
ERA5	ECMWF	GRIB2	https://cds.climate.copernicus.eu/
Upper atmosphere weather observation	NCEP but downloaded from Research Data Archive	little_r	https://rda.ucar.edu/datasets/d351000/
Surface Observational Weather Data		little_r	https://rda.ucar.edu/datasets/d461000/
SMOKE	CMAS center	Fortran source code	https://www.cmascenter.org/smoke/
Emissions inventories (US)	US EPA	text, csv	https://gaftp.epa.gov/Air/nei/
Emissions inventories (Canada)	ECCC	csv	https://open.canada.ca/data/en/dataset/fa1c88a8-bf78-4fcb-9c1e-2a5534b92131
EPA’s emissions modelling platforms	US EPA	Scripts (csh, py), text, csv, FF10, and executables	https://gaftp.epa.gov/Air/emismod/
CMAQ (including MCIP)	CMAS center	Fortran source code	https://www.cmascenter.org/cmaq/
Northern Hemisphere CMAQ runs	EPA	netCDF	https://drive.google.com/drive/folders/1A1ZzJE1t7OgwSezQNvy3rt9aATnXA0k2
NAPS observations	ECCC	csv	https://open.canada.ca/data/en/dataset/1b36a356-defd-4813-acea-47bc3abd859b
Census boundary files	Statistics Canada	Shapefiles	https://www12.statcan.gc.ca/census-recensement/2011/geo/bound-limit/bound-limit-2016-eng.cfm
Population age and sex Catalogue number: 98-400-X2016003	Statistics Canada	csv	https://www12.statcan.gc.ca/census-recensement/2016/dp-pd/index-eng.cfm
Annual postal code level PM_2.5_ and NO_2_	CANUE	csv	https://www.canuedata.ca/index.php
Monthly existing PM_2.5_ from Satellite data	Washington University in Saint Louis	netCDF4	2000-2018 V4: https://wustl.app.box.com/v/ACAG-V4NA03-PM25/folder/119797060328 2019-2020 V5: https://wustl.app.box.com/v/ACAG-V5GL0502-GWRPM25/folder/293391109905
High resolution Local Climate Zones	World Urban Database and Access Portal Tools	TIFF	https://zenodo.org/records/8419340
ETOPO 2022 Global Relief Model (Bedrock)	NOAA	netCDF	https://www.ncei.noaa.gov/products/etopo-global-relief-model
NRN	Statistics Canada (publisher)	Shapefile	https://open.canada.ca/data/en/dataset/3d282116-e556-400c-9306-ca1a3cada77f
ODIAC Fossil Fuel CO₂ Emissions	NASA	TIFF	https://db.cger.nies.go.jp/dataset/ODIAC/DL_odiac2022.html
Python scikit learn	Community project	Package (python codes)	https://scikit-learn.org/stable/

### 4.1 CMAQ simulations at 12 km resolution

Input preparation is a critical step in atmospheric modelling, and determines its success in capturing the atmospheric states. Similar to other regional-scale CTMs, CMAQ is mainly driven by two important inputs i.e., meteorology and emissions. The meteorological fields are created using the Weather Research and Forecasting (WRF) [9]. We run WRF [versions 3.9.1 (2000-2017) & 4.3.3 (2018-2020)]. WRF is initialized with the European Centre for Medium-Range Weather Forecast (ECMWF) fifth generation global reanalysis dataset (ERA5), the entire dataset that is publicly available and was obtained through ECMWF Climate Data Store. ERA5 was chosen after a sensitivity run that showed that it outperformed the US National Center for Environmental Prediction (NCEP) North American Regional Reanalysis (NARR) data in Ontario, Quebec, and British Columbia for O_3_. To reduce the model’s error propagation, the meteorological simulations are run daily with a 6 h spin up period for each day. WRF’s OBSGRID tool was used to infuse the observational datasets NCEP ADP Global Surface and Upper Air Observational Weather Data with nudging with used surface observations that were blended into the ERA5 analysis using WRF’s OBSGRID tool. Both observational datasets were obtained from the Research Data Archive at the National Center for Atmospheric Research (NCAR-RDA). WRF selected physics options include WRF Single-Moment 5-class scheme (microphysics), Noah Land Surface Model (land surface), Yonsei University scheme (planetary boundary layer scheme), Kain-Fritsch scheme with default trigger (Cumulus Parameterization).

Emissions for base CMAQ simulations were processed using the Sparse Matrix Operator Kernel Emissions (SMOKE) and inputs from EPA’s emissions modelling platforms. The US Environmental Protection Agency (EPA) periodically prepares emissions modelling platforms to facilitate air quality modelling. These platforms include both criteria and toxic pollutants. The emissions data are based on the National Emissions Inventories (NEIs) for the US, and the Canadian Air Pollutant Emission Inventories (APEI). The platforms also include the latest available Environment and Climate Change Canada (ECCC) inventory that matches the target year. National inventories take a tremendous amount of work and thus cannot be prepared on a yearly basis, which dictates that the platforms for non-inventory years be projected from inventory years. The boundary conditions were generated by running hemispheric CMAQ in-house, obtained from the EPA directly, or downloaded from the EQUATES publicly available dataset.

As CMAQ does not work directly with WRF outputs, a processing module, Meteorology-Chemistry Interface Processor (MCIP) [10] that carries out horizontal and vertical coordinate transformations on WRF outputs and calculates other fields needed for the CMAQ model to run. We ran two versions of CMAQ [5.2 (2000-2017) and 5.4 (2018-2020)] [11] as the simulations were conducted throughout multiple years. CMAQ simulations were conducted for a domain covering most of North America with a horizontal grid spacing of 12-km for the years 2000-2020. The modelling domain is concentric with EPA’s 12US1 domain but extends further north and east to suit our need to cover more Canadian territory refer to Figure A3 in the appendix. The domain extends 551 cells east to west, 391 cells north to south, and 35 layers vertically. The simulations were run on the computational resources of the Digital Research Alliance of Canada. For performance evaluation in Canada, we use observations from the National Air Pollution Surveillance (NAPS) network. NAPS assembles a national database by collecting the measured pollutant concentrations from local municipal, provincial, and territorial monitoring networks. It is coordinated by ECCC which is also responsible for the program’s quality assurance [12]. The hourly CSV NAPS files for PM_2.5_, NO_2_, and O_3_ are publicly available on the ECCC website. The stations are spatially matched to the 12-km CMAQ grid cells, then hourly/daily time series from the model and observations are compared and analyzed using codes written in the programming language python.

### 4.2 Census division

Health data at times are provided at spatial resolutions coarser than those of the CMAQ simulations. This necessitates providing concentration surfaces at geographic denominations and formats such as census divisions that may be more convenient for health studies. Thus, we processed CMAQ hourly outputs into local time using a time-zone conversion file. To have a common CD reference throughout the study period, the year 2016 was selected. Census boundaries and a higher-resolution dissemination areas (DA) population were taken from Statistics Canada processed using GIS tools to match the 12-km grid-cells to CD boundaries. We then aggregated the 12-km grid-cells concentrations that are enclosed in a CD based on its population weight within said CD. This was done to avoid dilution in population exposure resulting from area weighting, and was particularly true for larger rural CDs. To carry out performance evaluation, NAPS stations falling in a CD were simply averaged and then compared to CD level concentrations.

### 4.3 Hybrid modelling

For the CMAQ-LUR dataset, we combined the CMAQ outputs with high-resolution LUR concentration datasets using techniques that resemble bias correction. The high-resolution annual datasets were sourced from the Canadian Urban Environmental Health Research Consortium (CANUE) from existing concentration datasets [2,13]. The resolution of the CANUE datasets was set to the 6-digit postal code, which varies in area between urban and rural settings, thus the spatial resolution varies dramatically between urban and rural settings [14]. The PM_2.5_ dataset incorporates a fusion of satellite-derived aerosol optical depth, chemical transport modelling, and ground-level measurements facilitated by the geographically weighted regression technique (GWR). Conversely, the NO_2_ dataset used for the hybrid model was compiled specifically for the year 2006 for Canada, leveraging background-level estimates from satellite retrievals, geographic variables, and deterministic gradients. The land use regression (LUR) model incorporates predictive variables such as proximity to significant emission sources and summer rainfall.

After discarding values falling outside of the CMAQ modelling domain, we matched the postal code centroids to the closest CMAQ grid-cell based on minimal centroid to center distance. Then, we applied both additive and multiplicative bias correction for fusing the daily CMAQ averages with the annual high-resolution dataset ((1), (2)). The two equations were adapted from simple bias correction techniques used in air quality forecasting ensembles [15]. Tests indicated that mixing the two methods resulted in better statistical performance. Thus, depending on the daily CMAQ values at each postal code, the mixed (hybrid) method will either use values produced by the additive method if the CMAQ daily average is lower than yearly average or the multiplicative method when the CMAQ daily value is higher than the annual average.

(1) $$C_{hybrid,additive}=\;C_{CMAQ\left(daily\right)}+\left(C_{GWR|LUR\left(annual\right)}-\;C_{CMAQ\left(annual\right)}\right)$$

(2) $$C_{hybrid,multiplicative}=\frac{C_{GWR|LUR\left(annual\right)}}{C_{CMAQ\left(annual\right)}}\times C_{CMAQ\left(daily\right)}$$

### 4.4 Machine learning (RF and HGB)

Machine learning has been used extensively in air pollution modelling in recent years. We employed two of the widely used supervised machine learning algorithms, namely random forest (bagging) and gradient booster (boosting). Random forest is an ensemble learning technique that merges regression prediction models based on many subsamples [16], reducing the risk of overfitting and is less sensitive to outliers. RF is relatively easy to use as it does not require scaling or extensive parameter tuning. Histogram gradient booster is an ensemble boosting algorithm that trains models consecutively, i.e., each model tries to rectify the errors made previously. It gets its name because it distributes values into discrete bins to construct histograms during training which improves speed and increases efficiency. Both algorithms were applied using python’s scikit-learn machine learning library.

To train the ML models we linearly interpolate base CMAQ run daily average target species concentrations (PM_2.5_, NO_2_, O_3_) in local time into the 1-km chosen domain covering the Canadian side of the mother domain to ensure a smooth surface without 12-km structural grid artifacts. Similarly, CMAQ meteorological inputs such as surface temperature, planetary boundary layer height, precipitation, wind speed, and wind direction were all interpolated. We also used high-resolution datasets population files (Dissemination Area) acquired from Statistics Canada. For land-use input data, we opted for the high-resolution local climate zone v3 [17] and altitude data (30 arc-seconds) from ETOPO 2022 Global Relief Model downloaded from NOAA. The Canadian national road network (NRN) was used to create four variables that represent the length of each road class (urban primary, rural primary, urban secondary, and rural secondary) within a grid-cell. As road information from NRN is static and does not convey any traffic count data, we used the 1-km by 1-km National Institute for Environmental Studies (NIES) open-source fossil fuel CO_2_ emissions [18] masked to the road network as a surrogate. Additionally, we used a day of the week classifier (weekday, Saturday, and Sunday) to account for temporal activity patterns. For PM_2.5_ models, we also used as inputs, existing publicly available datasets with monthly mean surface concentration estimates at a spatial resolution of 0.01° (∼1 km), the datasets are based on satellite retrievals, ground observations, and global CTM model simulations [13,19].

**Fig. 7:**
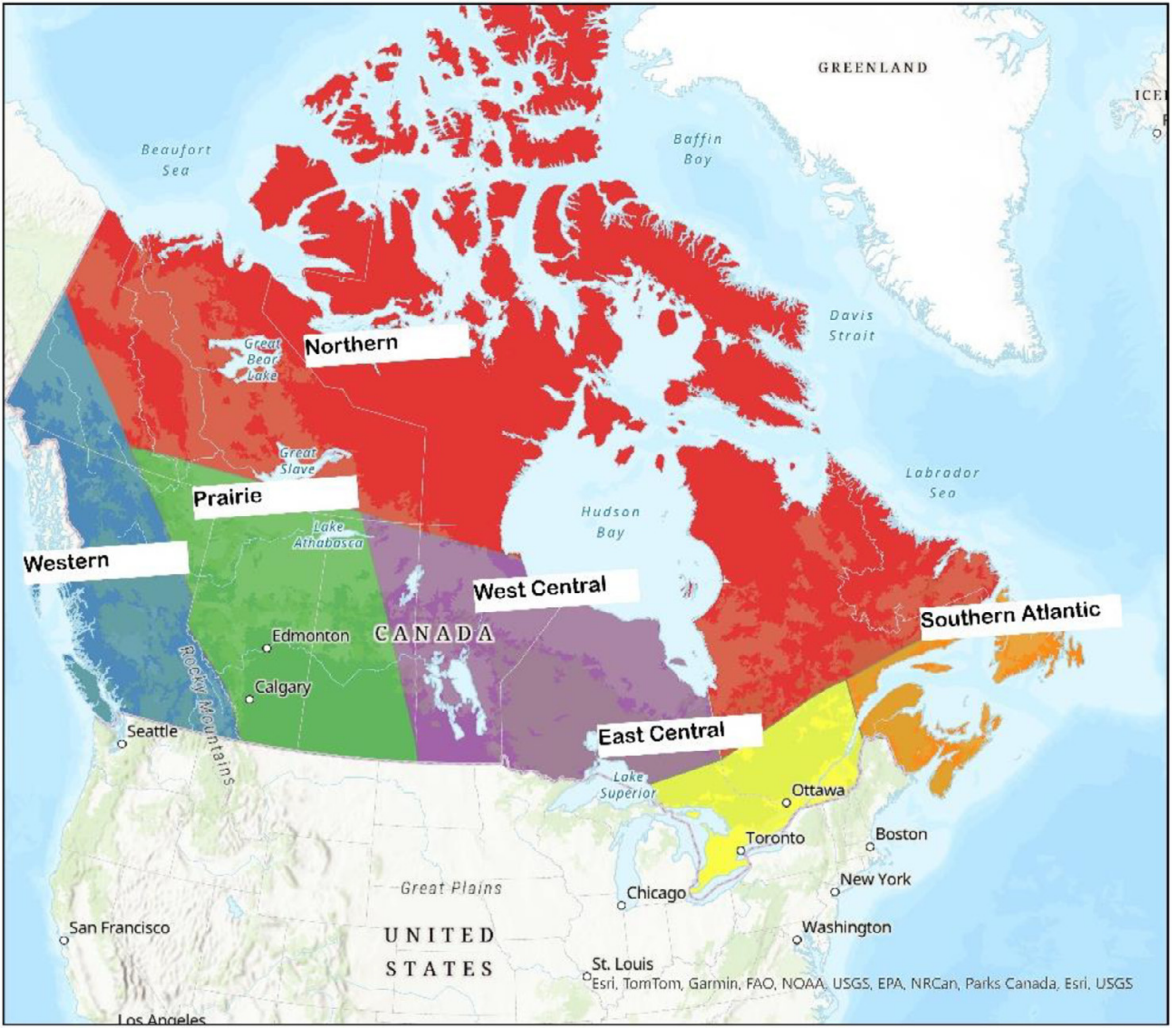
Canadian airsheds based on CCME classification.

To ensure proper representation across Canada, we applied cross-validation based on regions that resemble the airsheds used by the Canadian Council of Ministers of the Environment (CCME). The airsheds (Fig.7) are intended to tackle transboundary pollution and take into consideration large air masses movements, prevalent meteorological conditions over the long term, topography, and provinces air zone boundaries. Every year 20 % of the stations within an airshed were withheld, the model was trained in phases to match the inventory year used in SMOKE.

## Limitations

Due to the chaotic nature of the atmosphere, the large uncertainty in emissions estimates, and the limitations inherent in modelling choices, the outputs of chemical transport models are always subject to a range of uncertainties. As all our datasets are based on CMAQ simulations, these uncertainties exist and extend to all of them. While data fusion through bias correction and machine learning helps in improving the representativeness of the results, the underlying uncertainties are likely to persist. The efficacy of statistical methods is dependent on the accuracy and coverage of the training data.

Machine learning datasets were generated as a first attempt at exploring their viability, efficacy, and accuracy for downscaling CTM simulations. The performance and representativeness of ML datasets would closely depend on the quality of underlying inputs, particularly those at high resolutions. As a first attempt and proof-of-concept for a Canadian exposure dataset, detailed quality assurance was not feasible for this version of ML datasets as the evaluation of ML products should go beyond statistical performance to include close examination of spatial features; details that are not present in the provided ML datasets.

## Ethics Statement

The authors of this paper confirm that the work and datasets included in this article does not involve human subjects, animal experiments, or any data collected from social media platforms.

## CRediT Author Statement

**Anas Alhusban:** Conceptualization, Methodology, Project administration, Visualization, Investigation, Formal analysis, Validation, Data Curation, Software, Writing. **Burak Oztaner:** Conceptualization, Methodology, Project administration, Investigation, Software, Validation. **Markey Johnson:** Conceptualization, Funding acquisition, Methodology, Resources, Project administration. **Mastaneh Rezasefat:** Software, Investigation. **Negin Hojjatzadeh:** Investigation, Software. **Mahsa Soleimani:** Investigation. **Israa Moussa:** Investigation. **Saeed Nadi:** Investigation. **Shunliu Zhao:** Software. **Hwashin Shin:** Conceptualization, Funding acquisition, Project administration, Validation. **Joyce Zhang:** Project administration, Resources. **Amir Hakami:** Conceptualization, Methodology, Supervision, Funding acquisition, Resources, Writing.

All authors have contributed to the final manuscript preparation.

## Data Availability

FRDR)Longitudinal datasets (2000 – 2020) with high spatiotemporal resolution for air pollution exposure assessment in Canada (Original data).

## Appendix Supplementary materials

Image, application 1
