# Supplementary material for: Development of longitudinal datasets (2000–2020) with high spatiotemporal resolution for air pollution exposure assessment in Canada
Source: Data Brief. 2025 May 30;61:111730. doi: 10.1016/j.dib.2025.111730 (PMC12197882; doi:10.1016/j.dib.2025.111730)
Supplement: Supplementary file 1 [file mmc1.docx]

Data in Brief

Appendix for

**Development of longitudinal datasets (2000 – 2020) with high spatiotemporal resolution for** **air pollution exposure assessment in Canada**

**Authors**

Anas – Alhusban^1^, Yasar Burak – Oztaner^1^, Markey – Johnson^2^, Mastaneh – Rezasefat^1^, Negin – Hojjatzadeh^1^, Israa – Mousa^1^, Mahsa – Soleimani^1^, Saeed – Nadi^1^, Shunliu – Zhao^1^, Hwashin – Shin^3^, Joyce J. Y.– Zhang^2^, Amir – Hakami^1^**.*

**Affiliations**

1: Department of Civil and Environmental Engineering, Carleton University, Ottawa, Ontario, Canada.

2: Air Sectors Assessment and Exposure Science Division, Health Canada, Ottawa, Ontario, Canada.

3: Environmental Health Science and Research Bureau, Health Canada, Ottawa, Ontario, Canada.

**Corresponding author’s email address and Twitter handle**

*amir.hakami@carleton.ca*

**Introduction**

This File contains 1 table and 3 supplementary figures. Further information is provided on statistical performance (Table A1), stations-based performance for three pollutants (PM_2.5_, NO_2_, and O_3_) through Figure A1 (a,b,c) and Figure A2 (a,b,c), the CMAQ modeling domain (Figure A3).

| **Table A1: Statistical performance of different simulation phases** | | | | | | | | | | |
| --- | --- | --- | --- | --- | --- | --- | --- | --- | --- | --- |
| **Phase** | **Poll** | Test set (20% airshed) CMAQ | | | Test set (20% airshed) Random Forest | | | Test set (20% airshed) HGT | | |
|  |  | **R** | **MSE** | **MB** | **R** | **MSE** | **MB** | **R** | **MSE** | **MB** |
| Phase1  (2008-2014) | PM25 | 0.719 | 17.259 | 0.893 | 0.792 | 10.710 | 0.067 | 0.836 | 8.458 | 0.006 |
|  | NO2 | 0.700 | 31.789 | -0.555 | 0.847 | 13.487 | 0.165 | 0.905 | 8.378 | 0.038 |
|  | O3 | 0.543 | 86.827 | 0.973 | 0.742 | 44.789 | 0.004 | 0.815 | 32.530 | 0.068 |
| Phase2  (2000-2007) | PM25 | 0.767 | 46.983 | 1.751 | 0.834 | 29.536 | -0.083 | 0.877 | 25.102 | -0.057 |
|  | NO2 | 0.762 | 36.240 | -1.759 | 0.863 | 26.678 | 0.096 | 0.902 | 21.465 | 0.085 |
|  | O3 | 0.625 | 31.639 | 2.930 | 0.778 | 22.746 | 0.178 | 0.846 | 18.648 | 0.0362 |
| Phase3  (2015-2017) | PM25 | 0.605 | 51.702 | 1.717 | 0.759 | 31.789 | 0.139 | 0.761 | 29.563 | 0.118 |
|  | NO2 | 0.702 | 45.571 | -0.258 | 0.829 | 32.733 | -0.145 | 0.902 | 24.879 | 0.085 |
|  | O3 | 0.388 | 35.323 | 2.642 | 0.716 | 20.184 | -0.017 | 0.805 | 16.535 | 0.002 |
| Phase4  (2018-2020) | PM25 | 0.639 | 42.711 | -0.223 | 0.828 | 32.676 | 0.034 | 0.845 | 28.362 | 0.014 |
|  | NO2 | 0.781 | 38.912 | -1.098 | 0.847 | 32.122 | -0.101 | 0.903 | 25.606 | -0.087 |
|  | O3 | 0.603 | 23.878 | -0.241 | 0.786 | 19.318 | -0.210 | 0.841 | 16.119 | -0.234 |


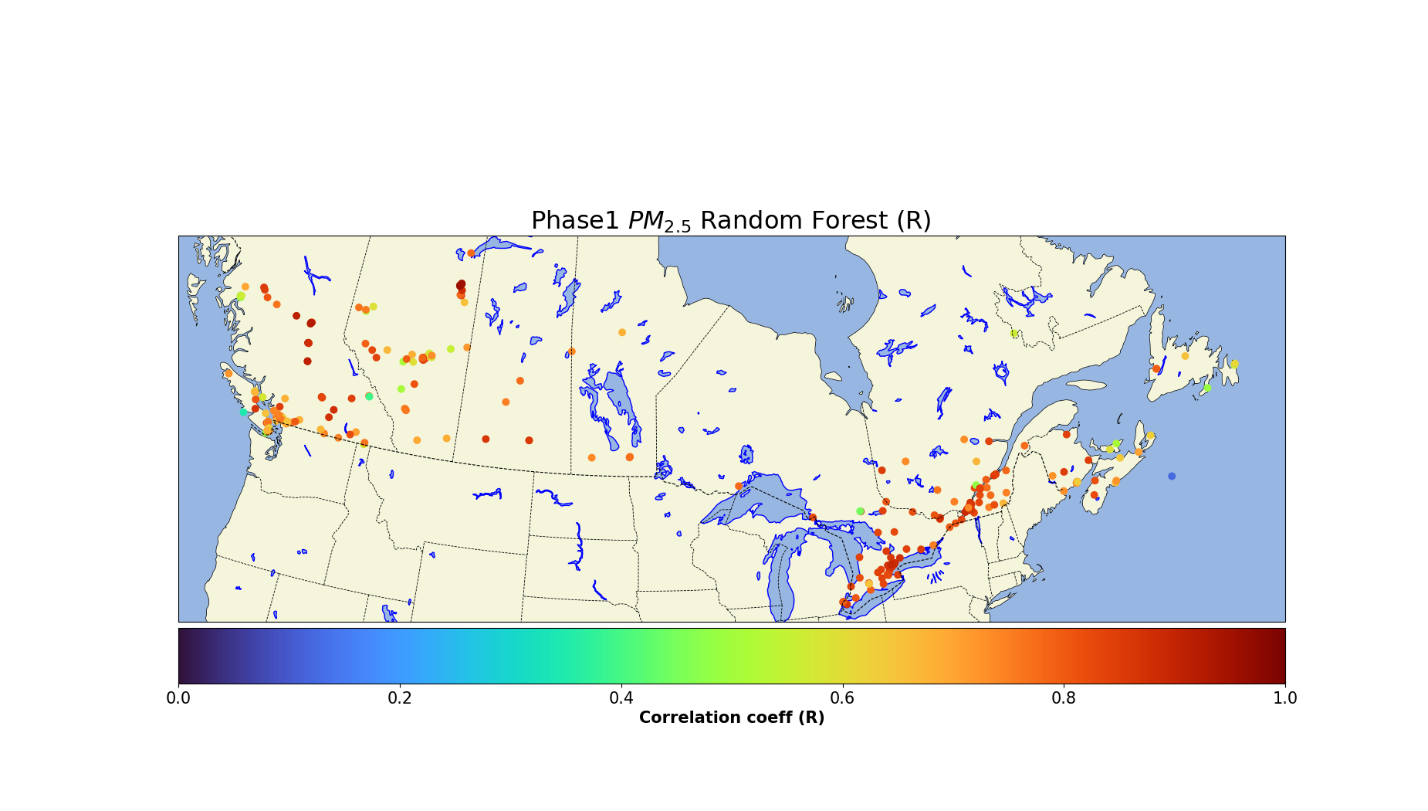

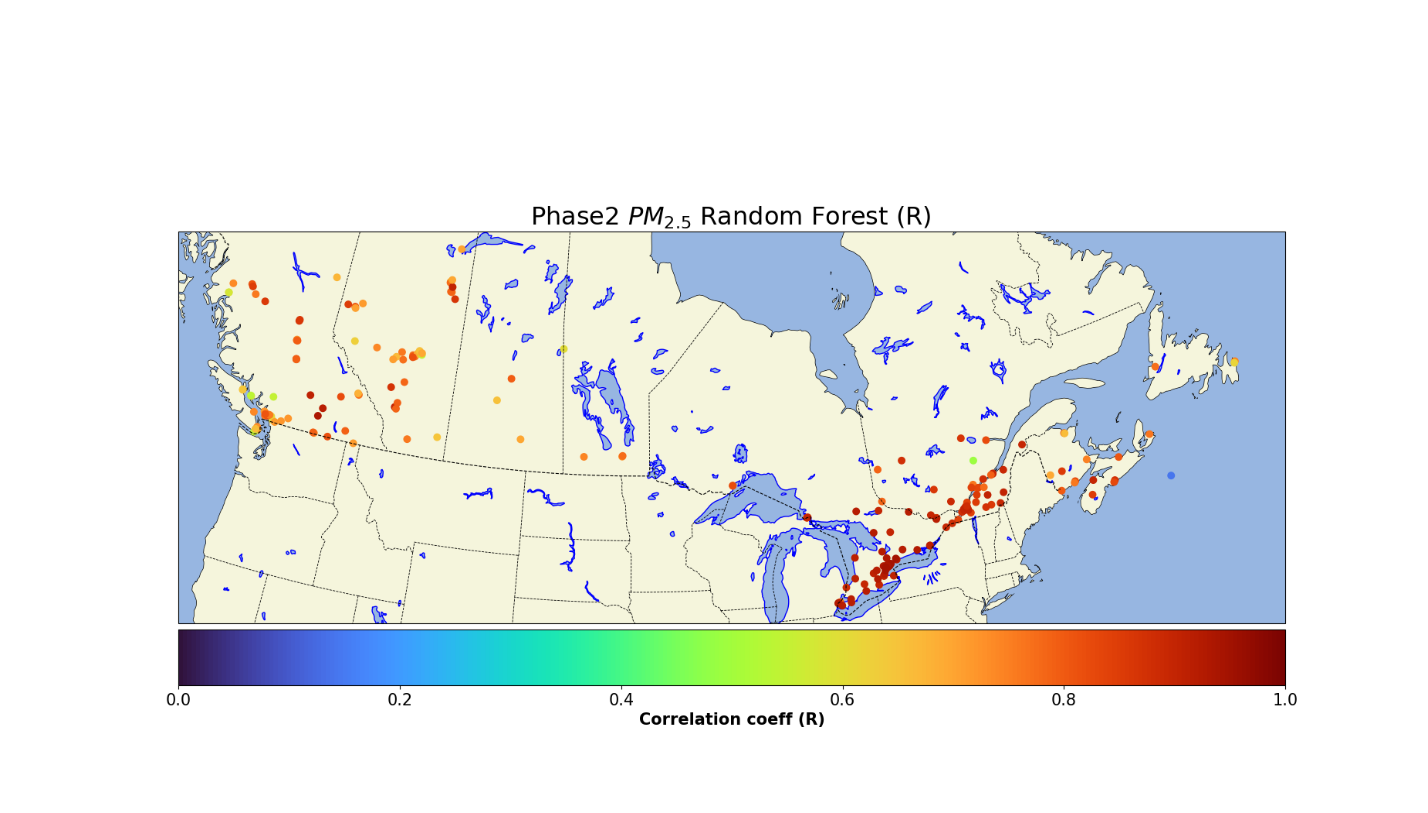

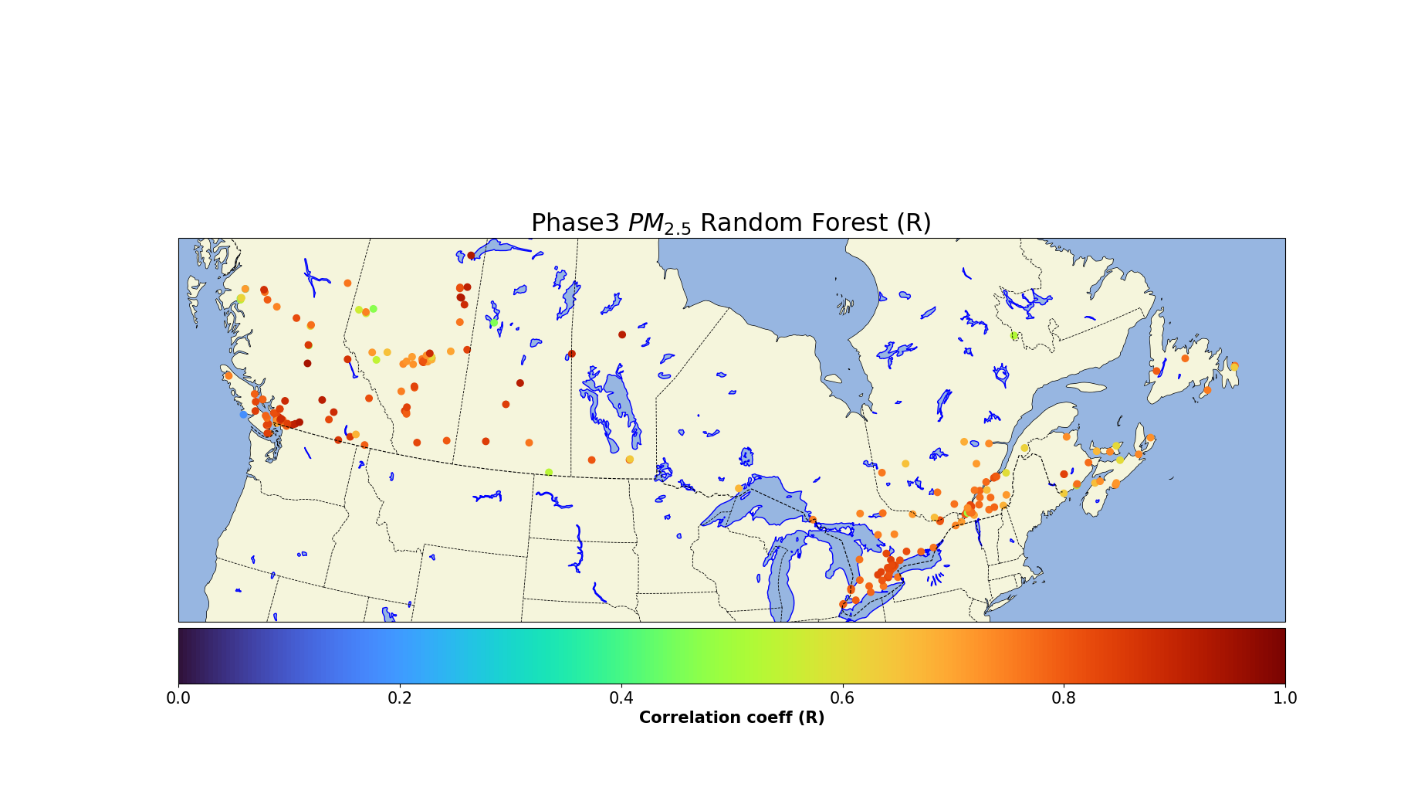


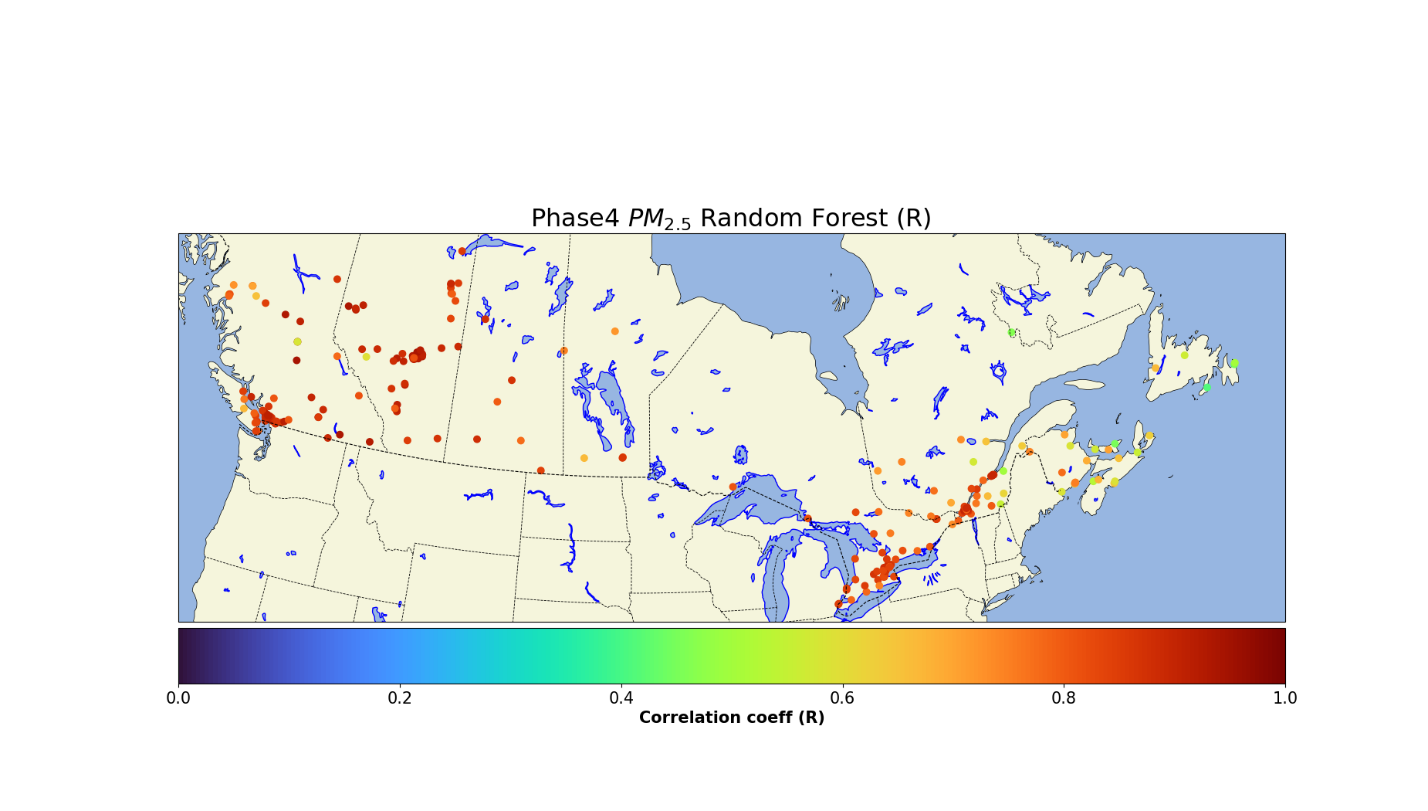


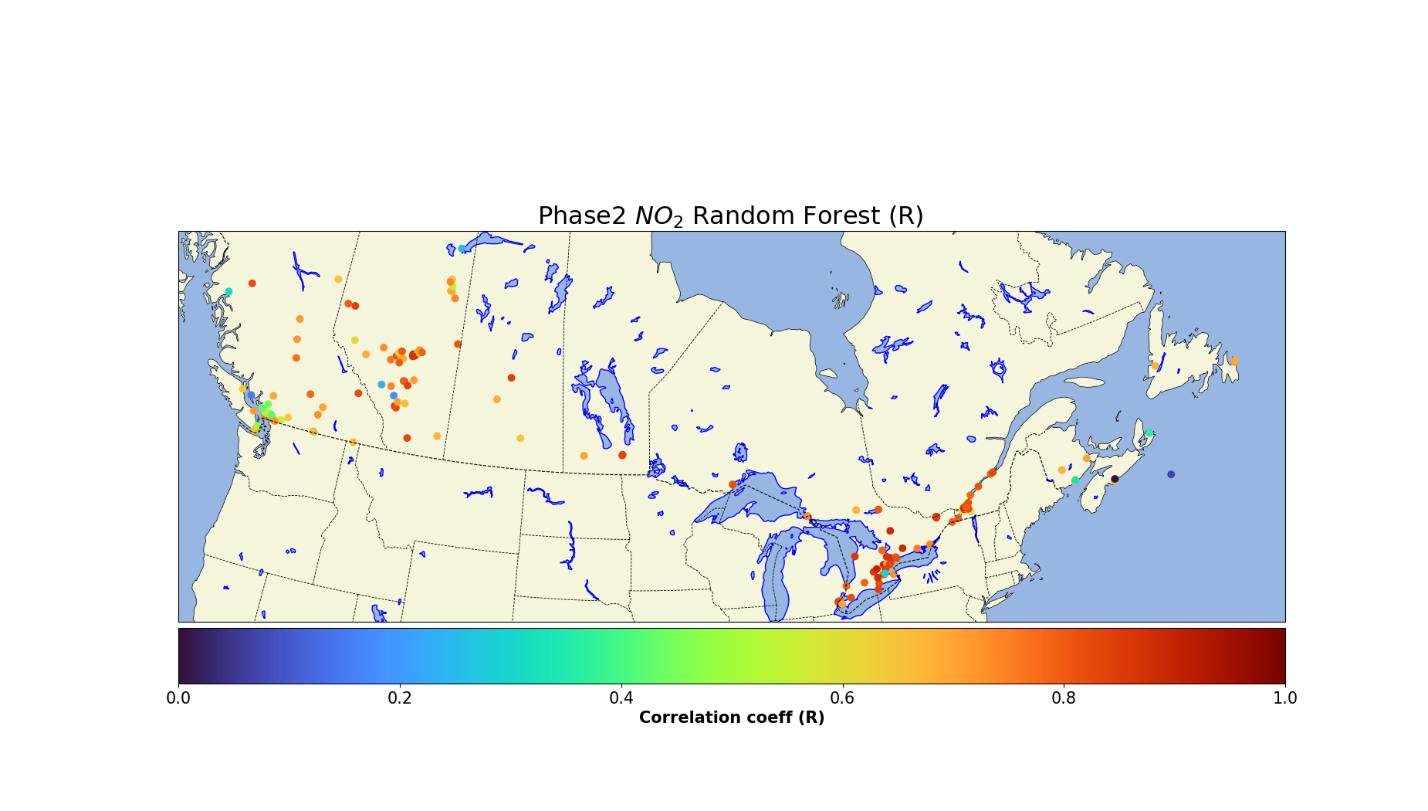

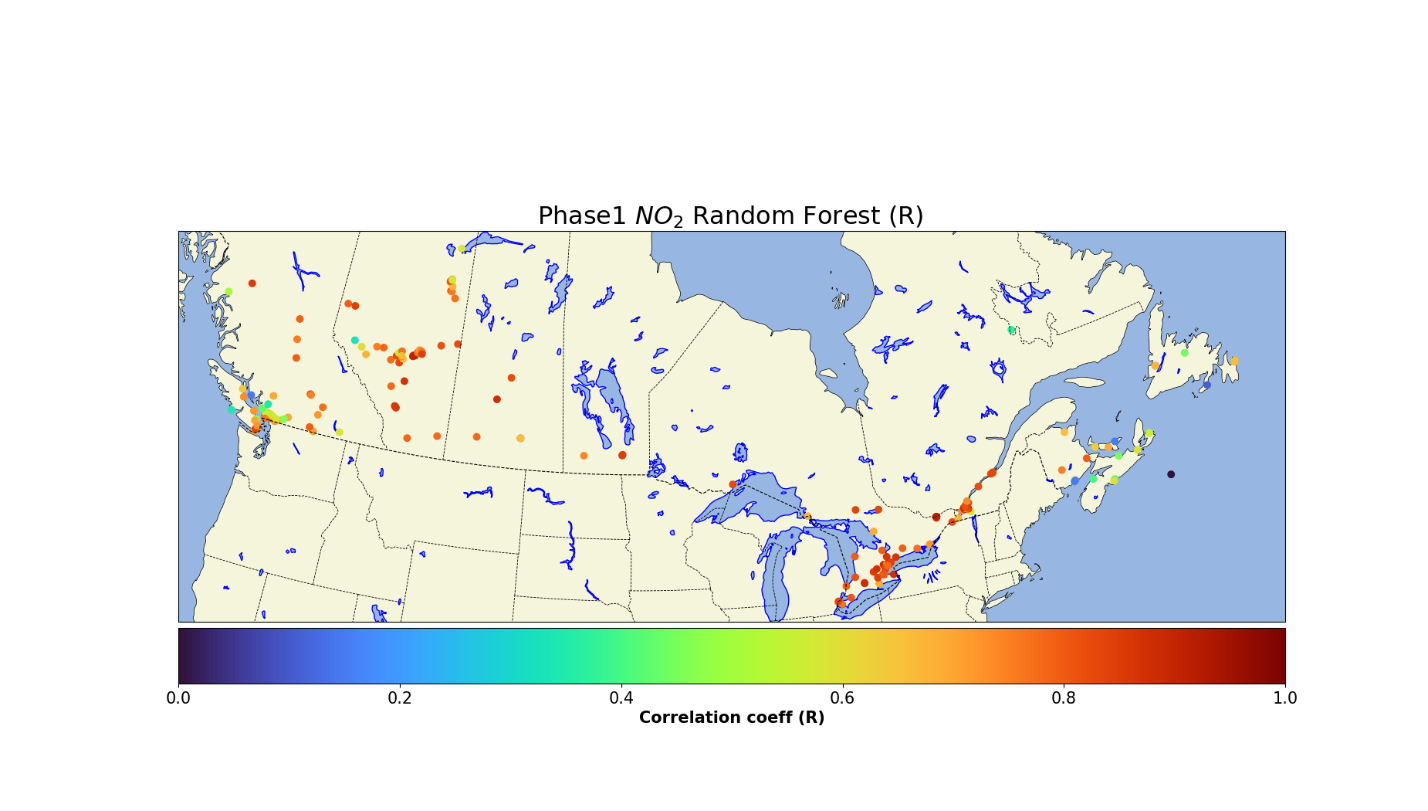


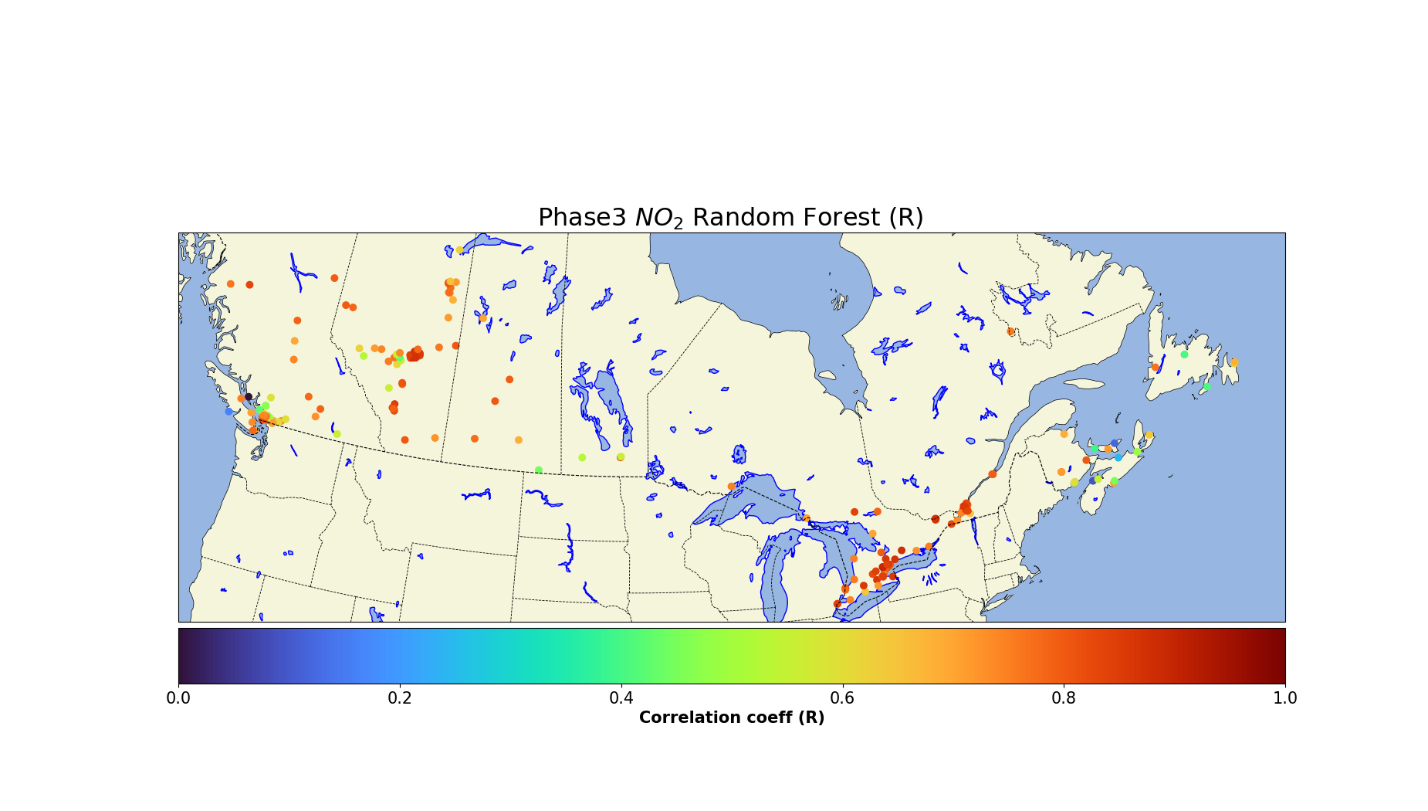


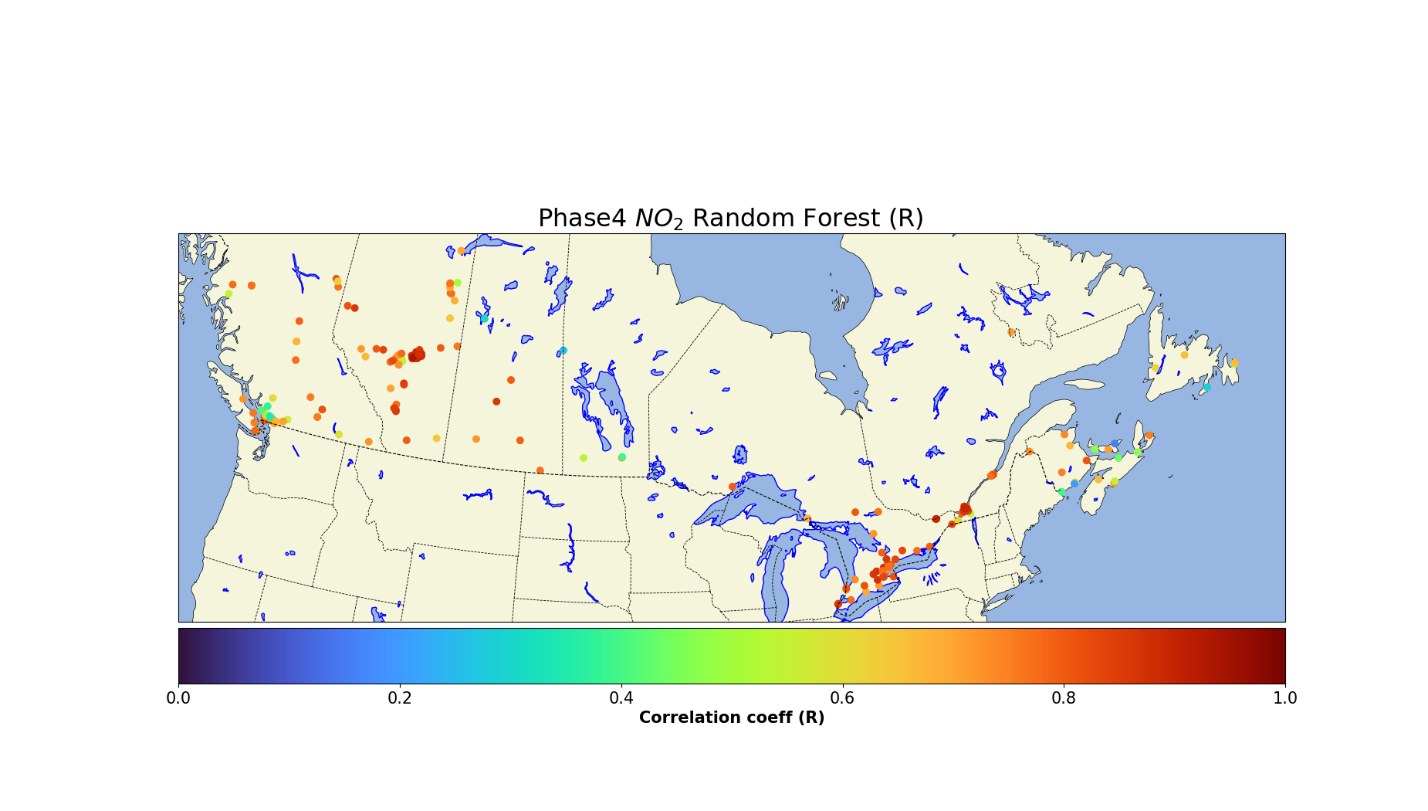


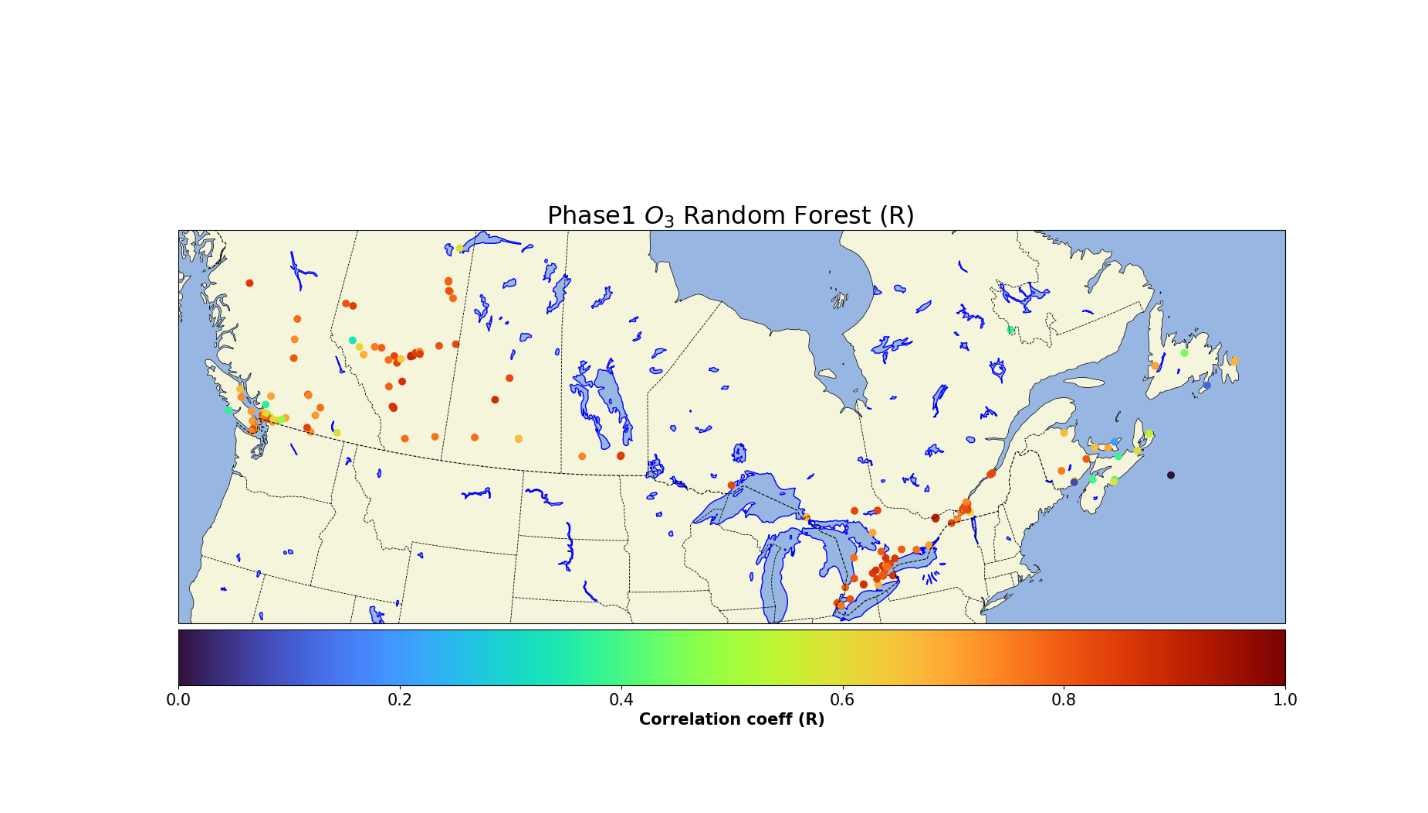


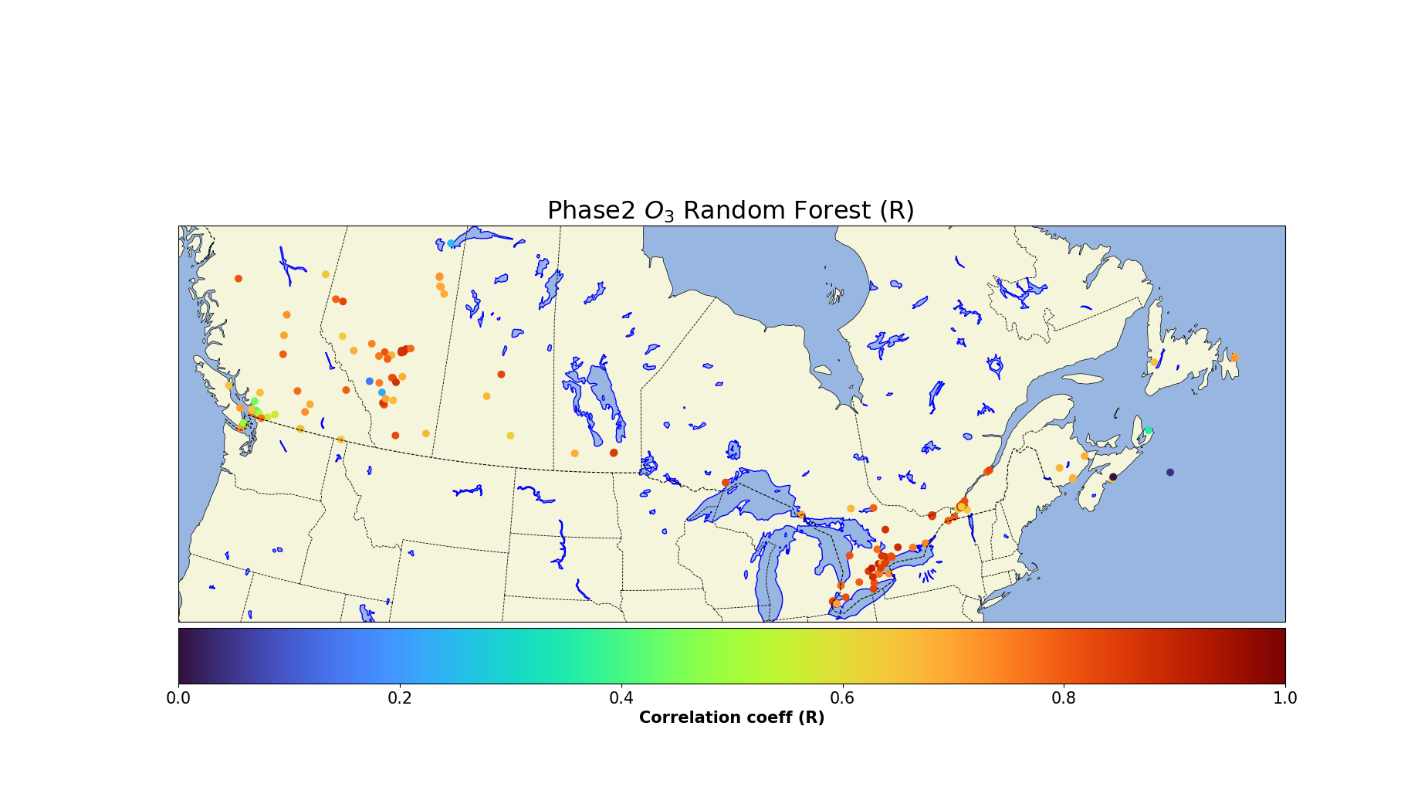


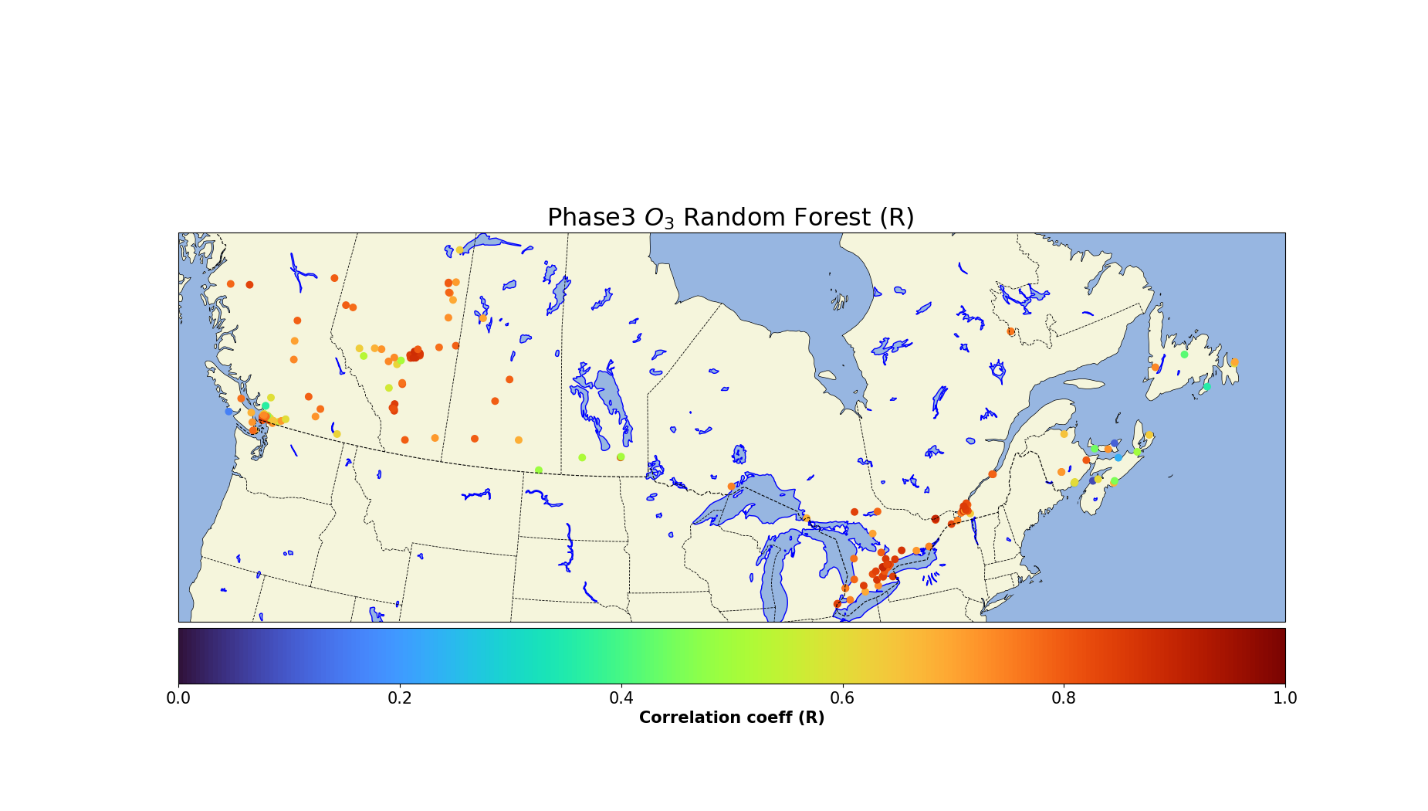


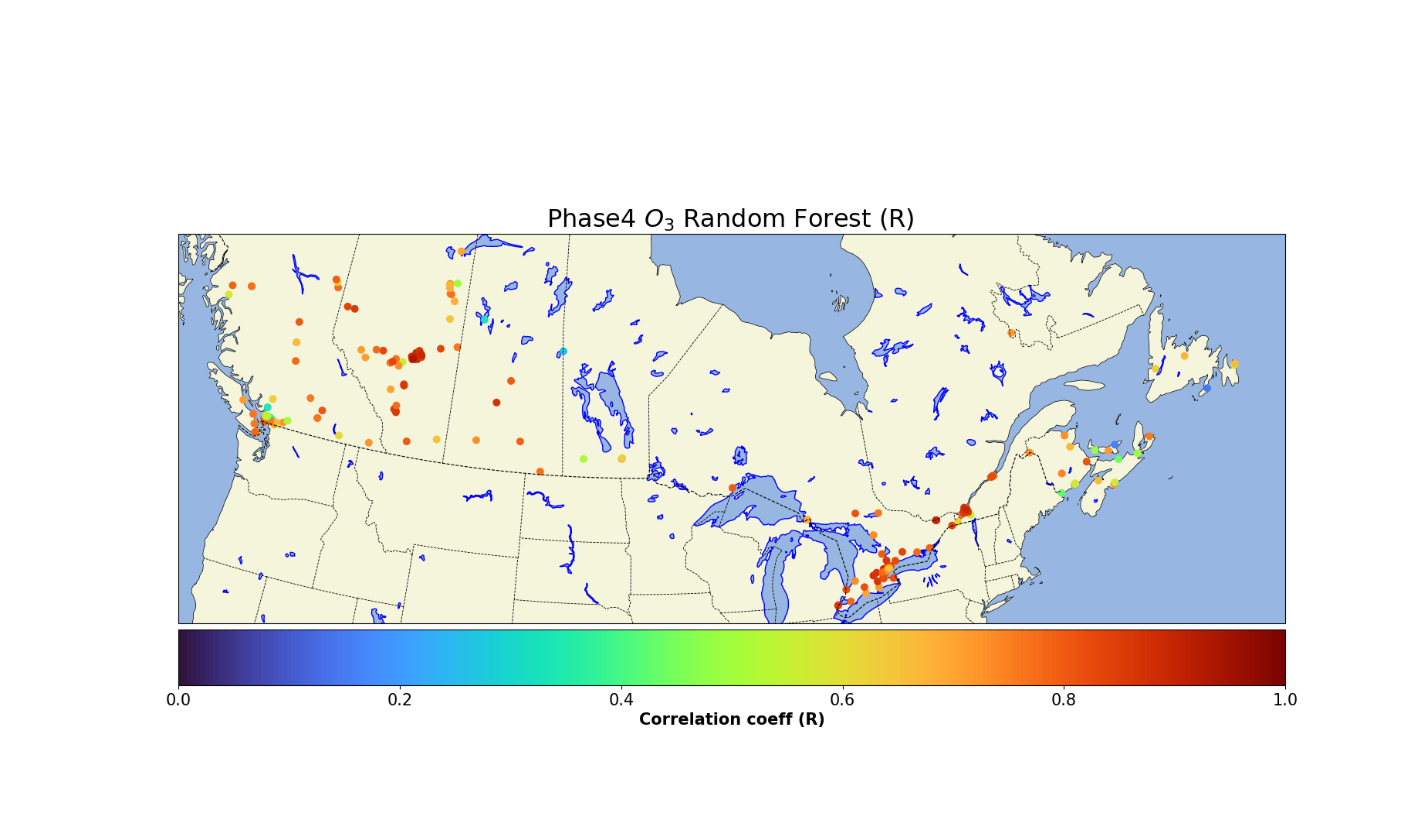


Figure A1 panel (a): Stations based correlation coefficient phase-wise Random Forest for PM_2.5_, NO_2_, and O_3_.


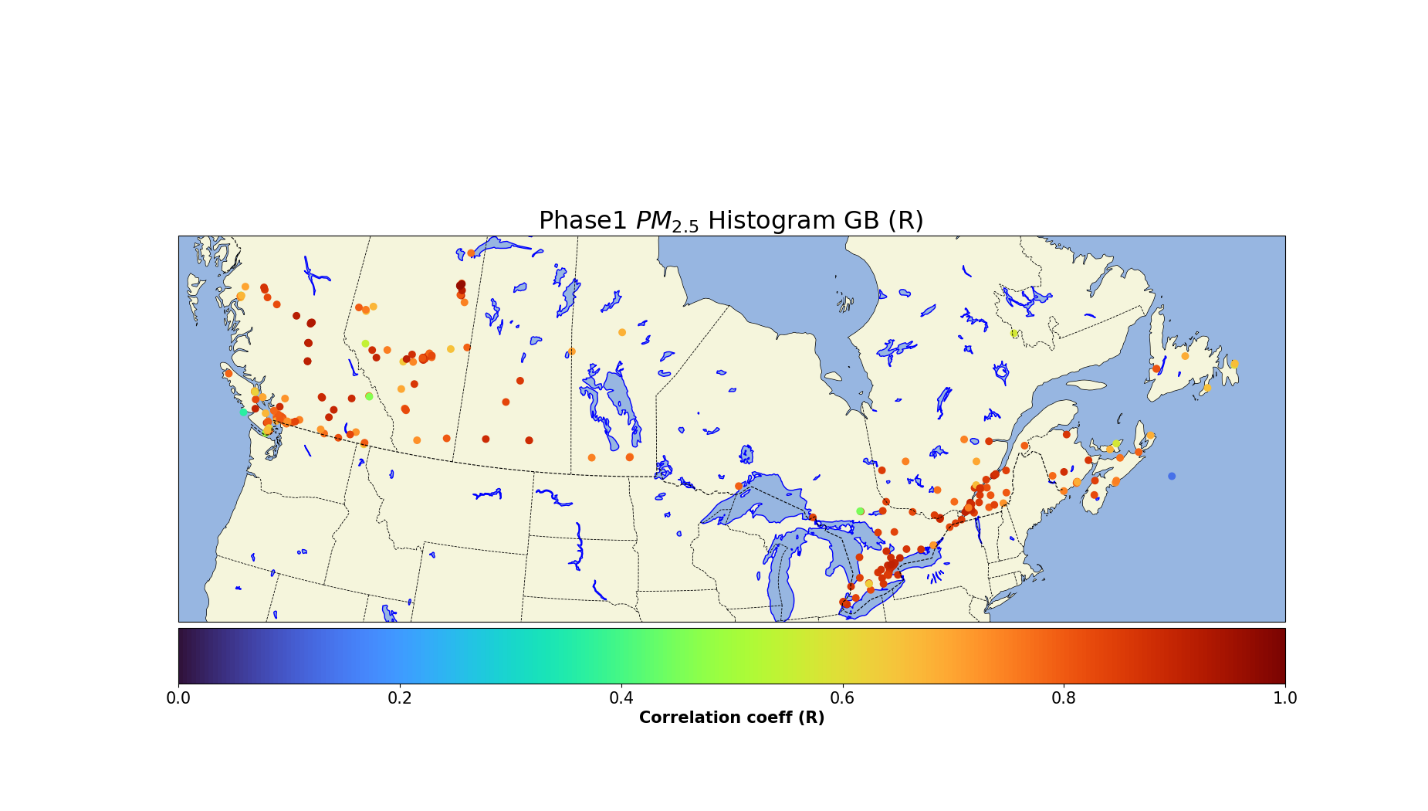


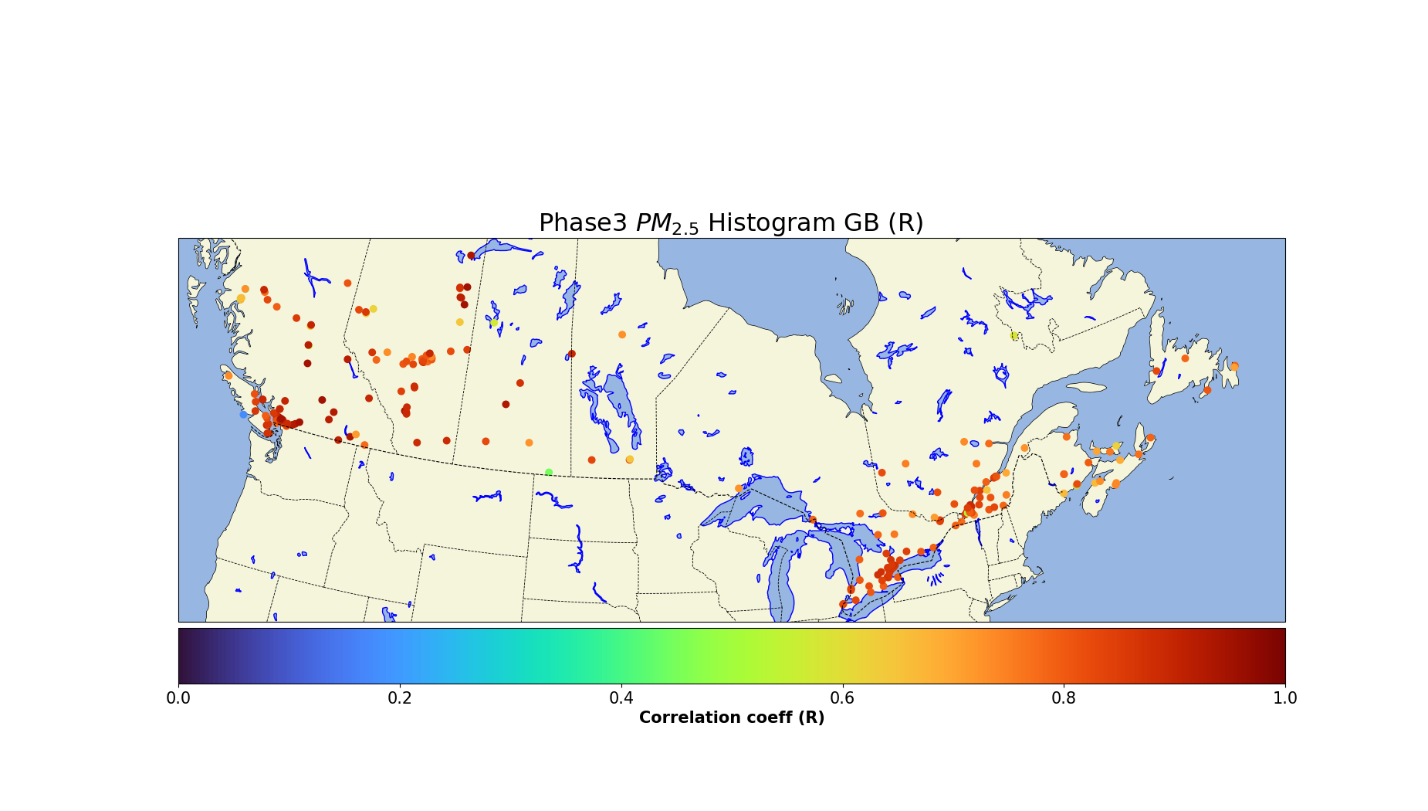

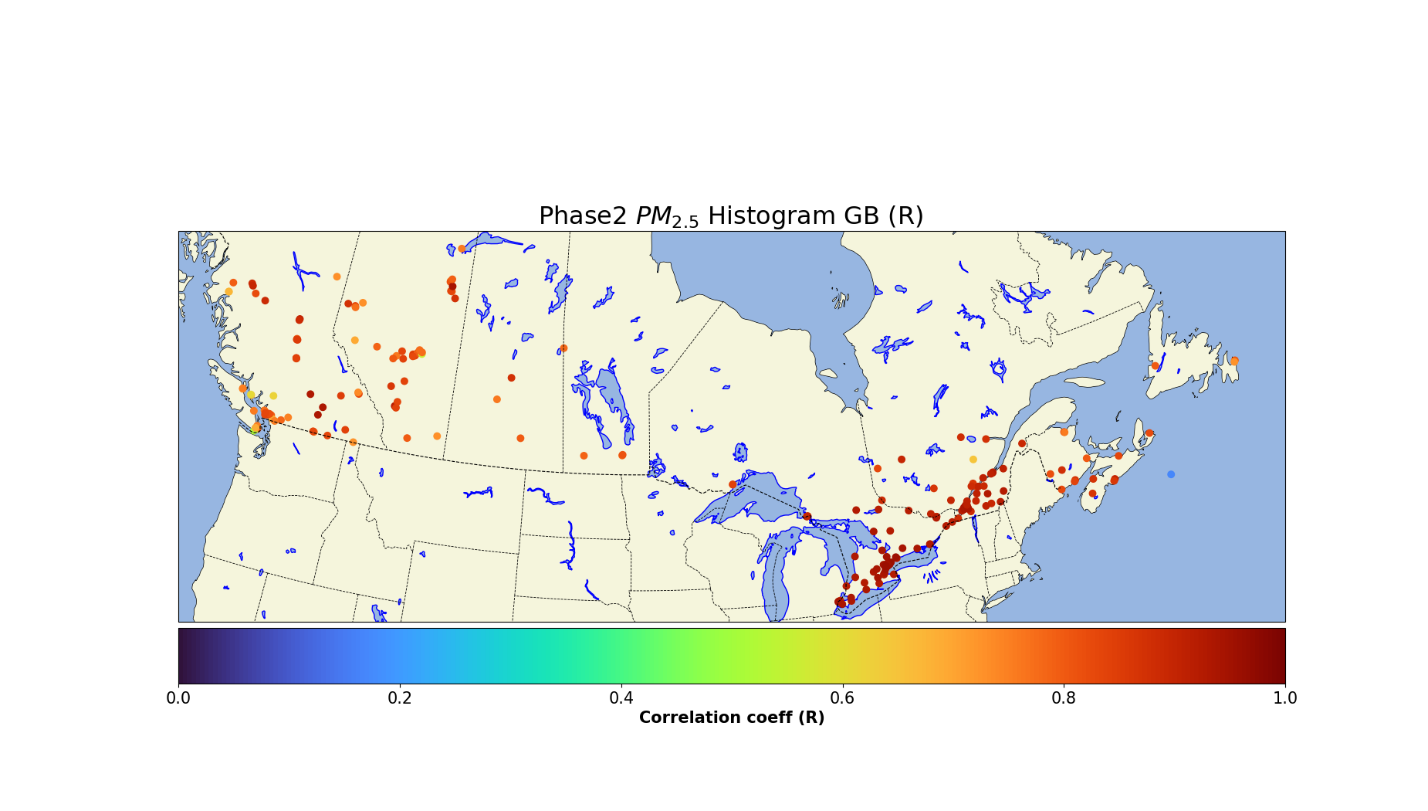


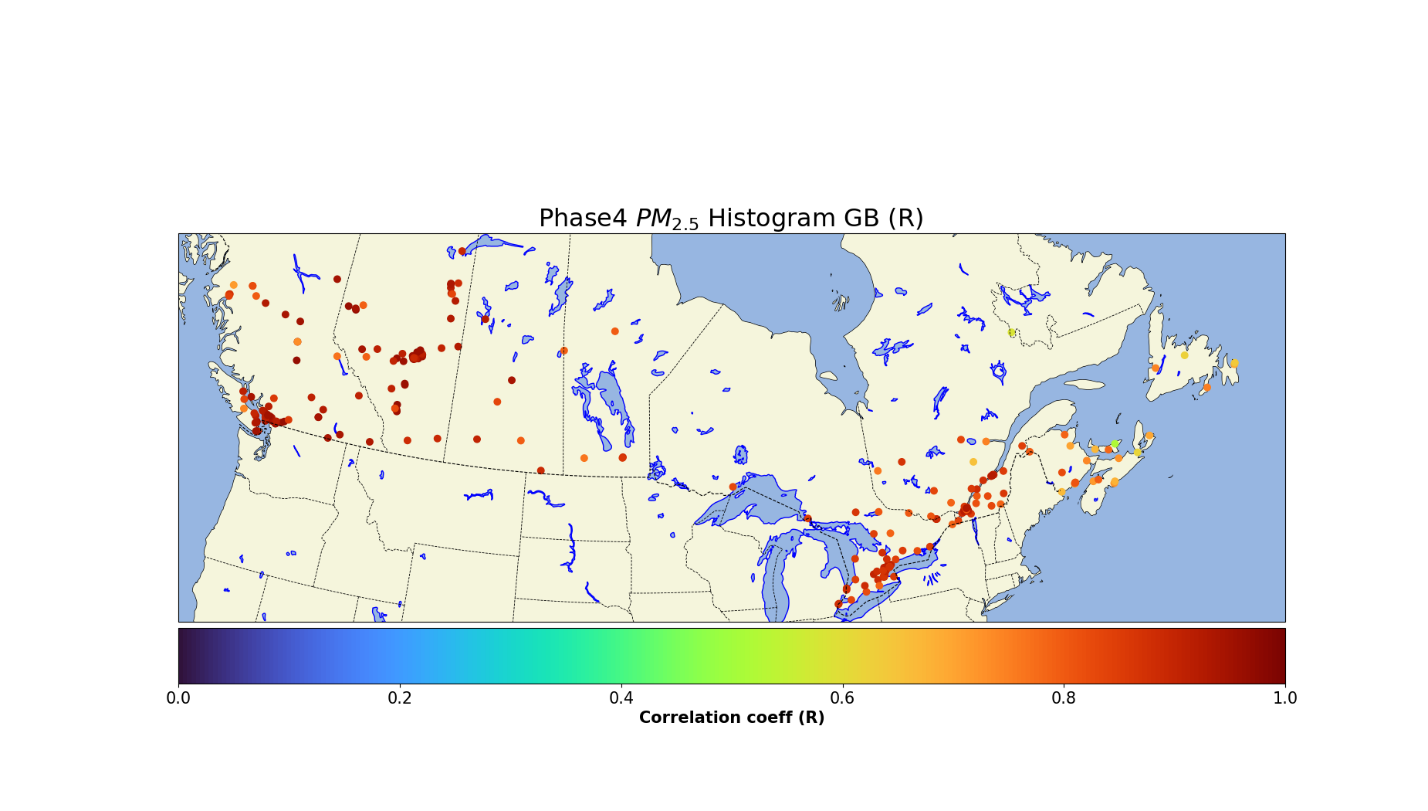


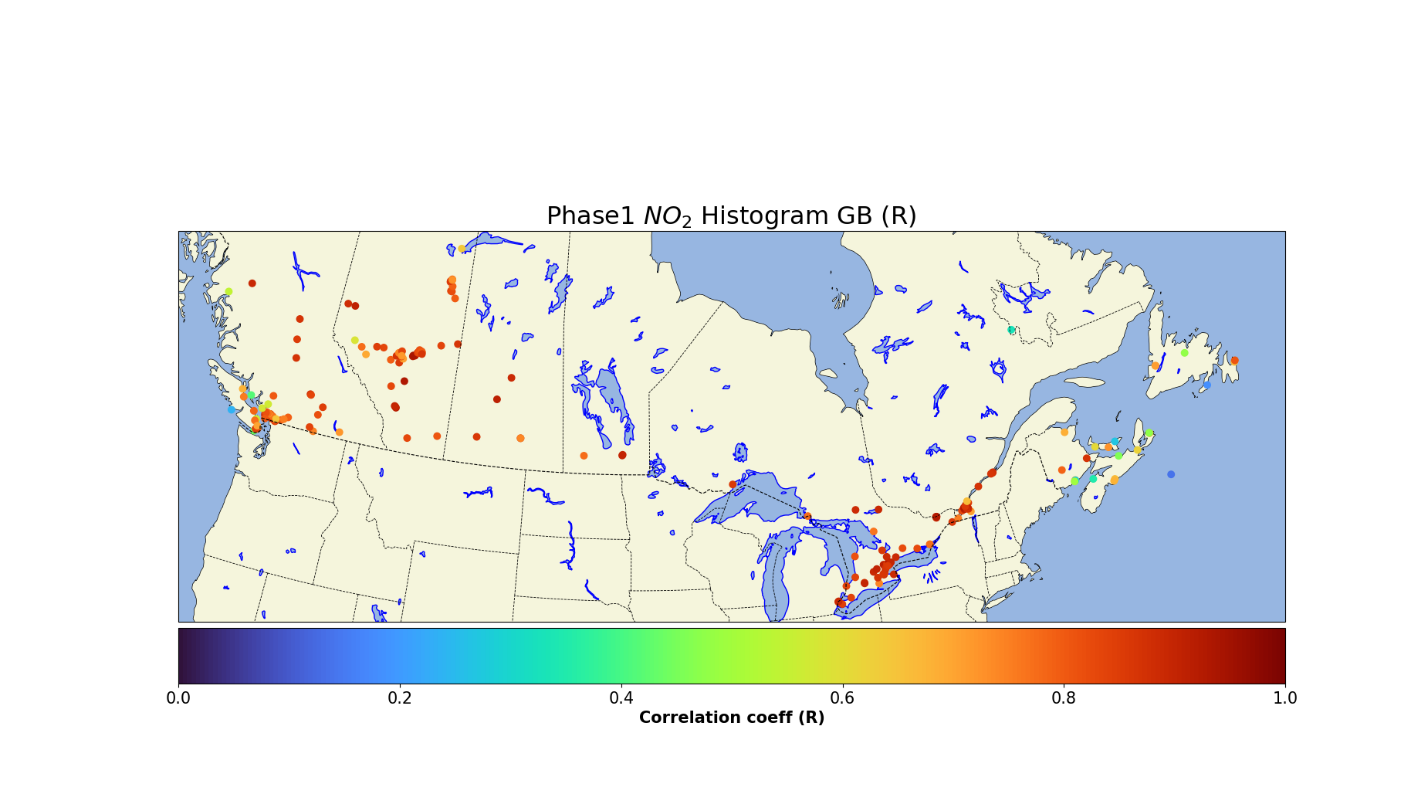

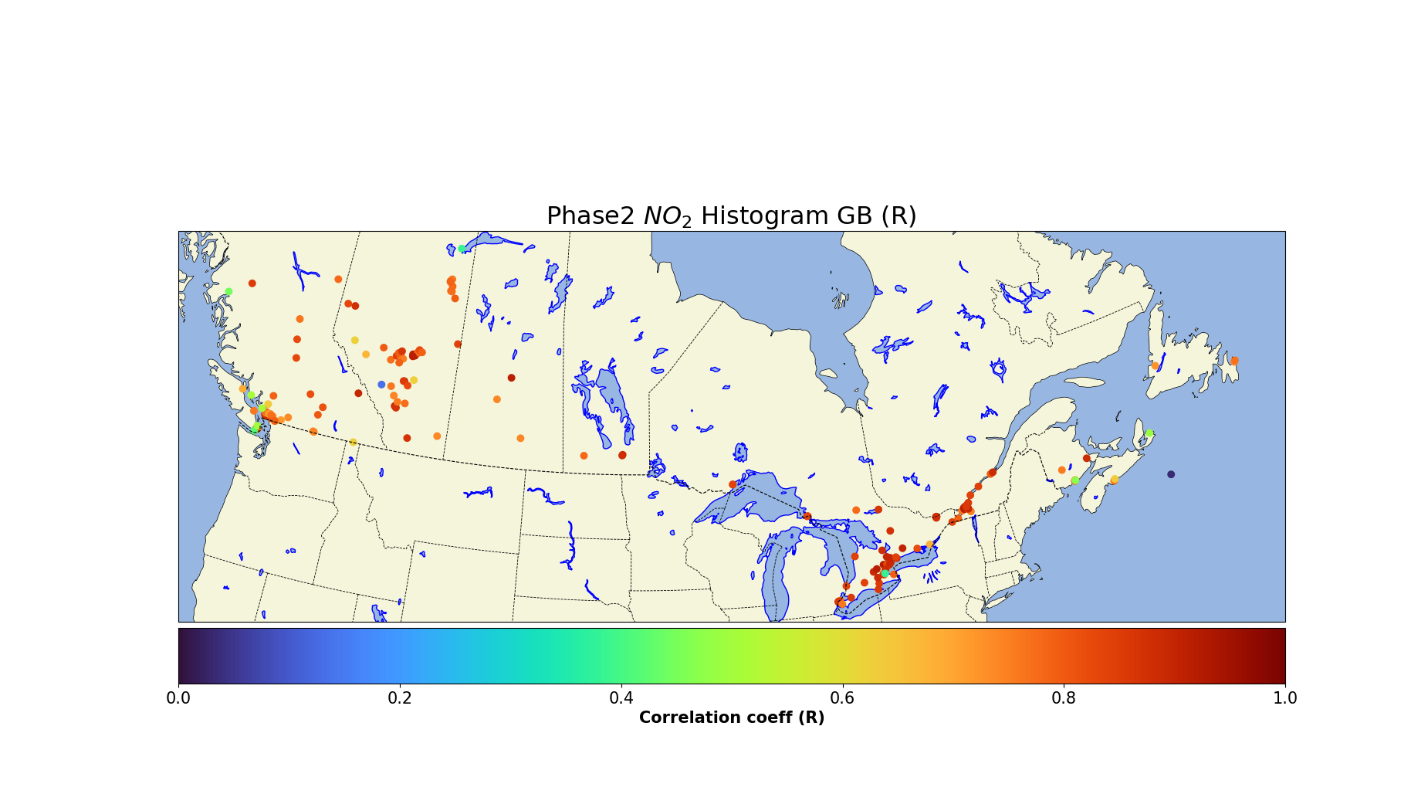


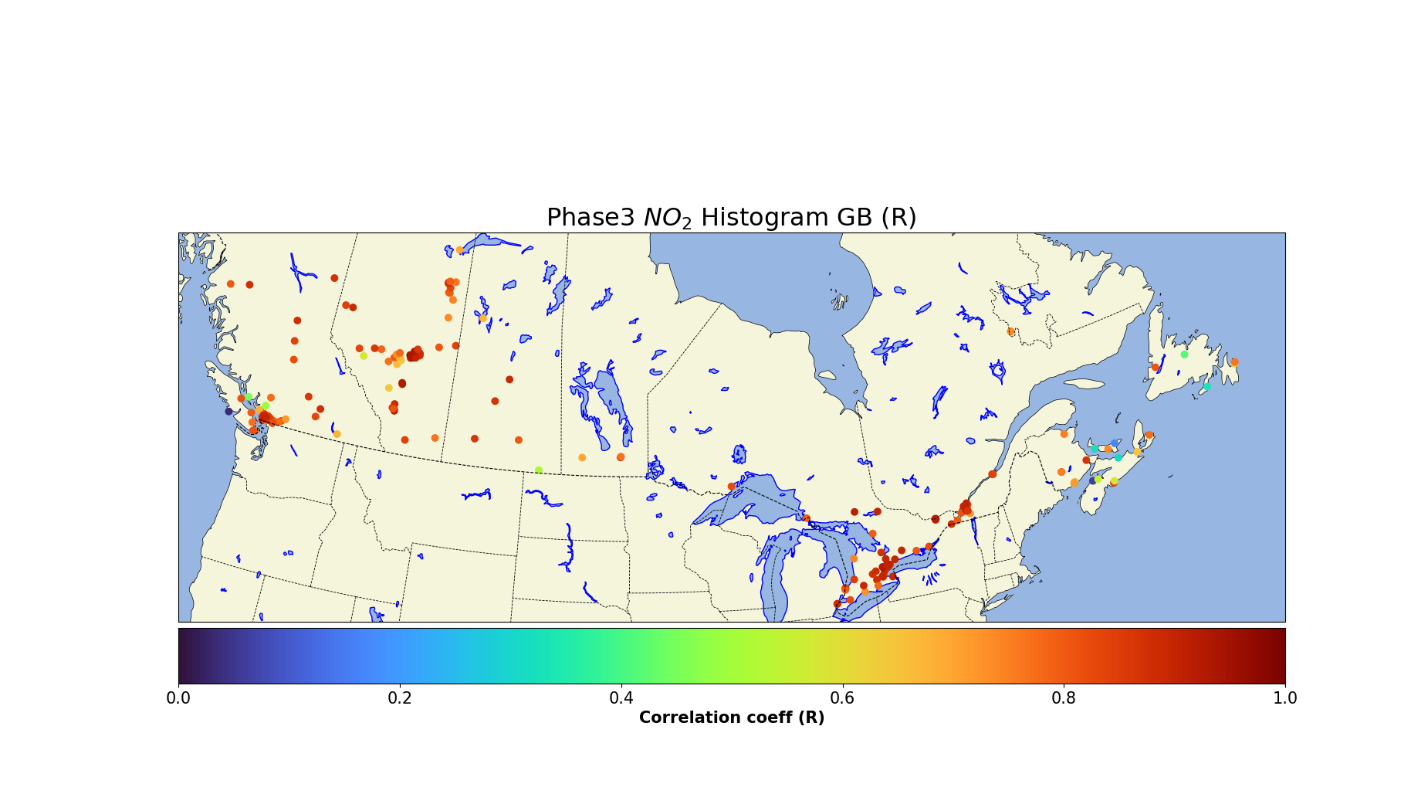


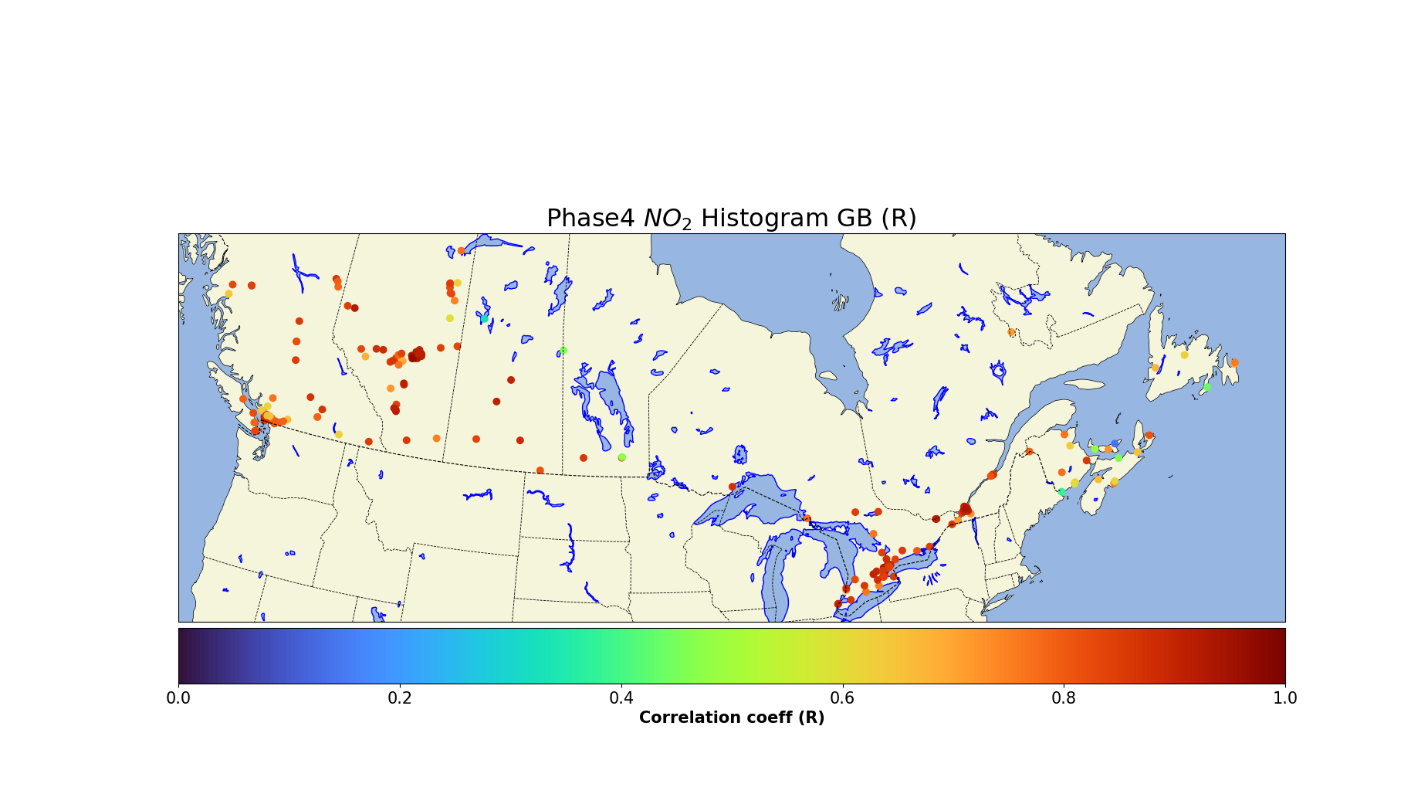


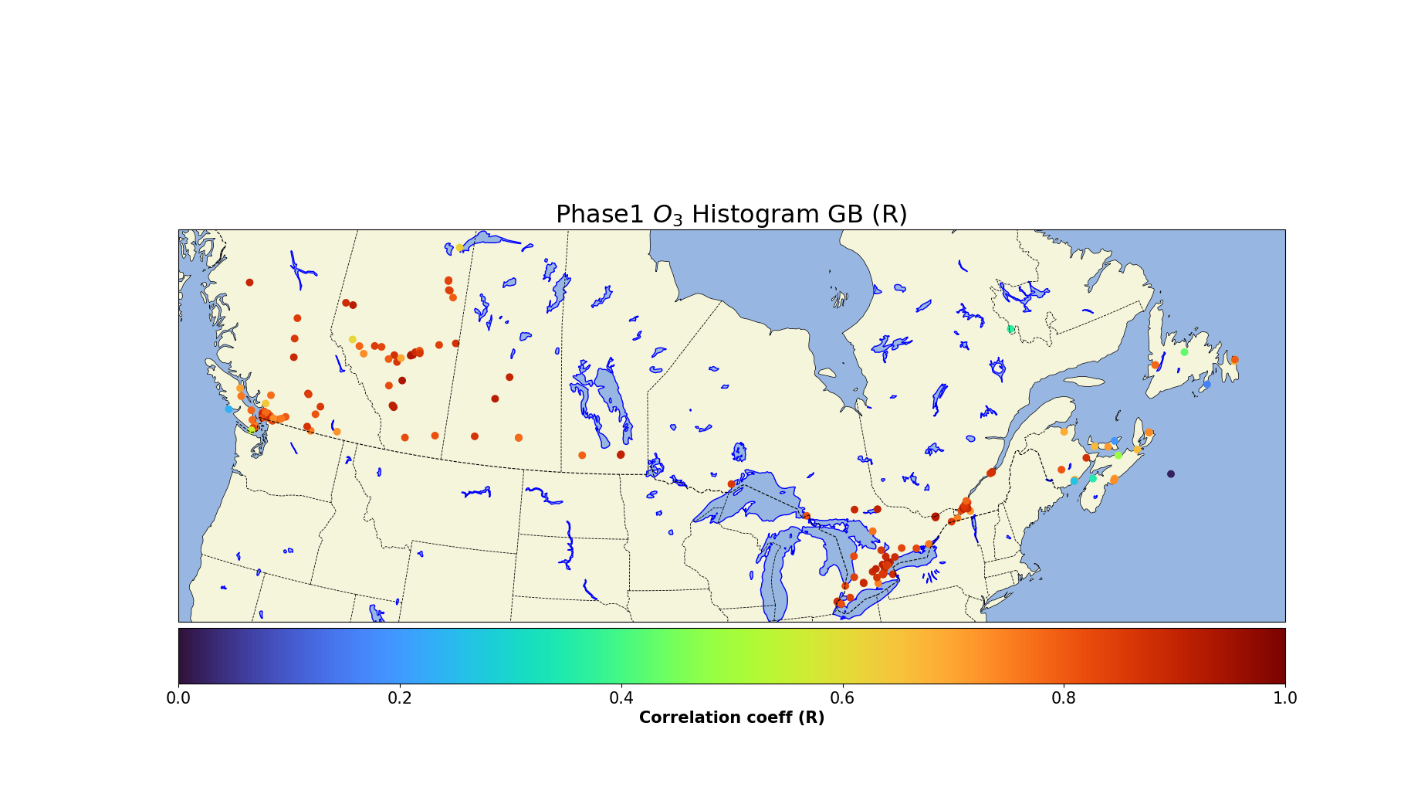


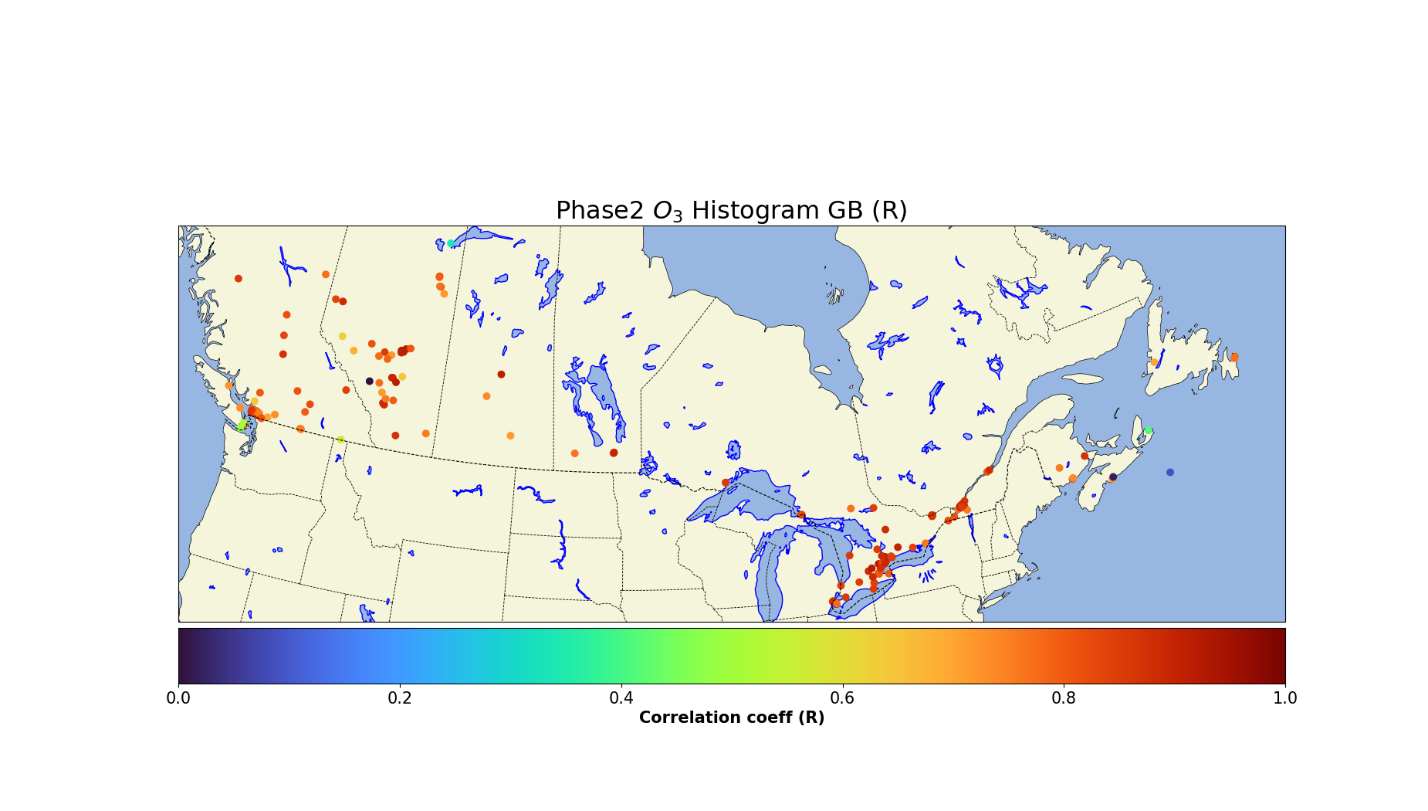


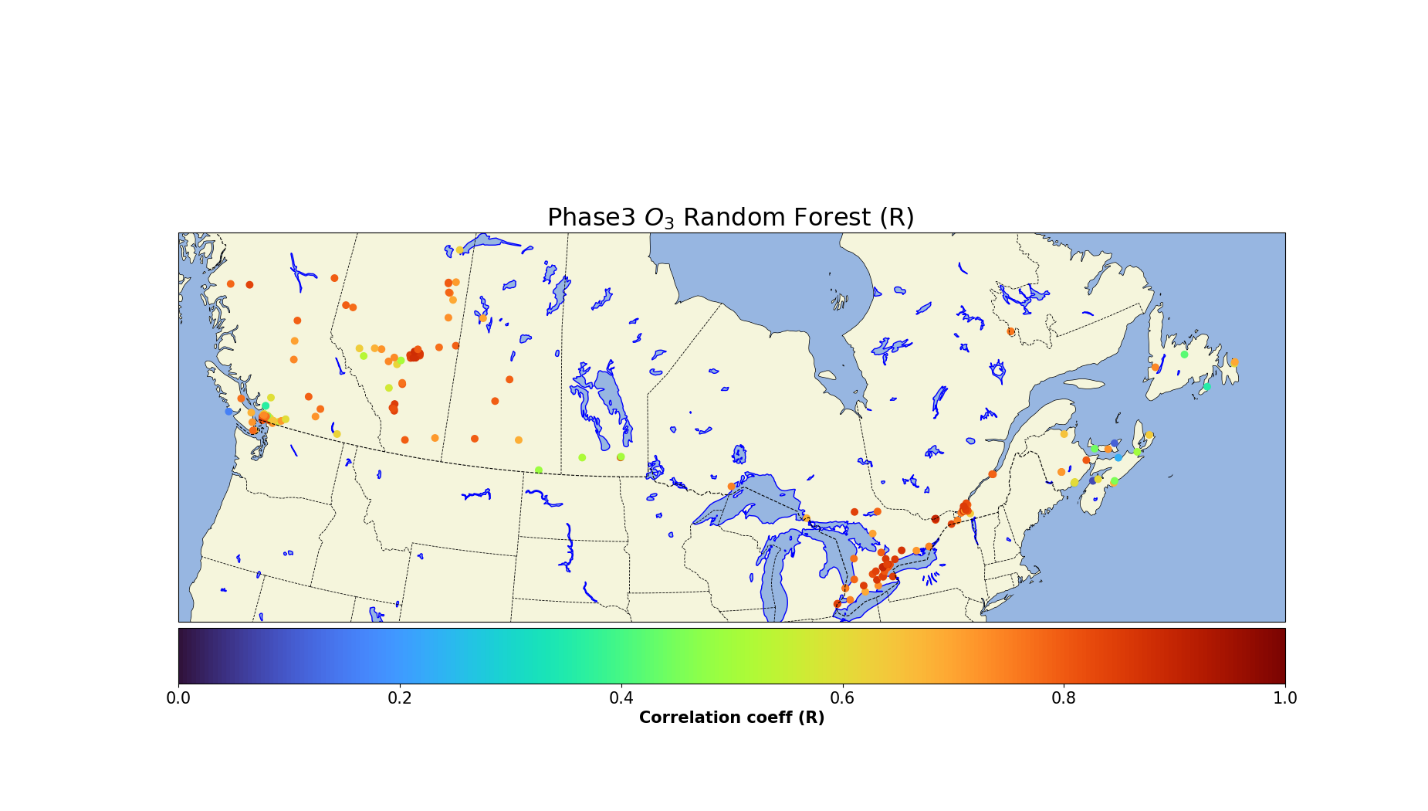


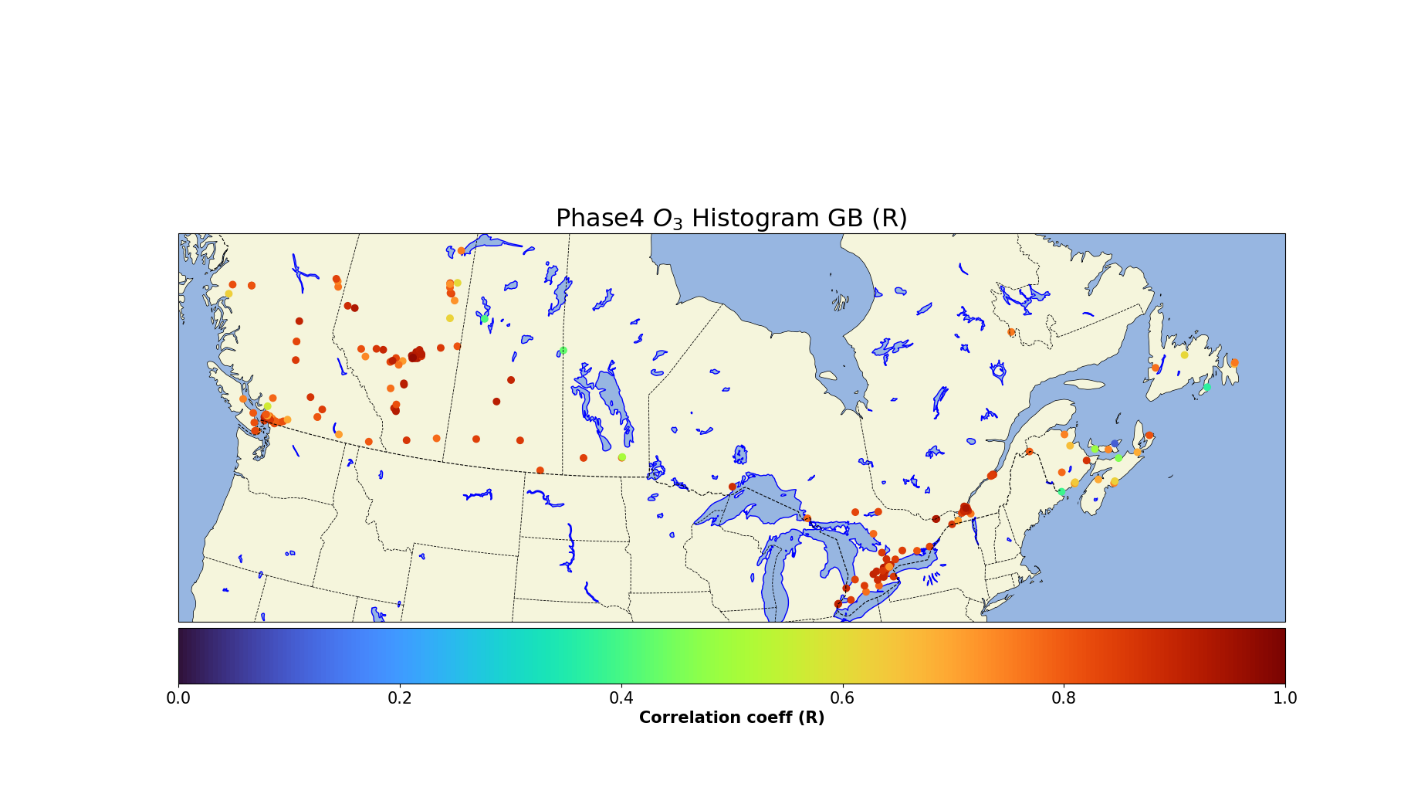
**Figure A1 panel (b): Stations based correlation coefficient phase-wise Histogram Gradient Booster for PM_2.5_, NO_2_, and O_3_.**


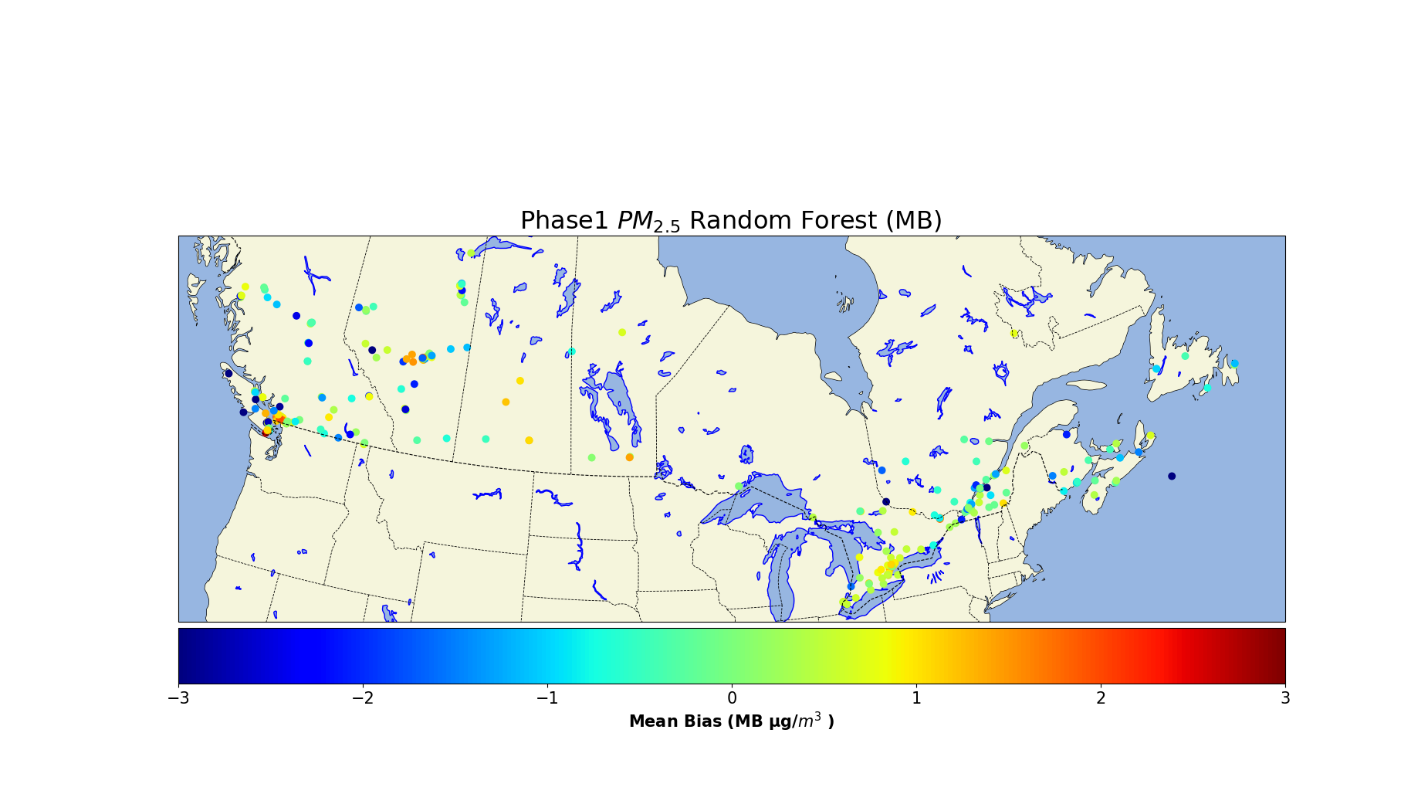

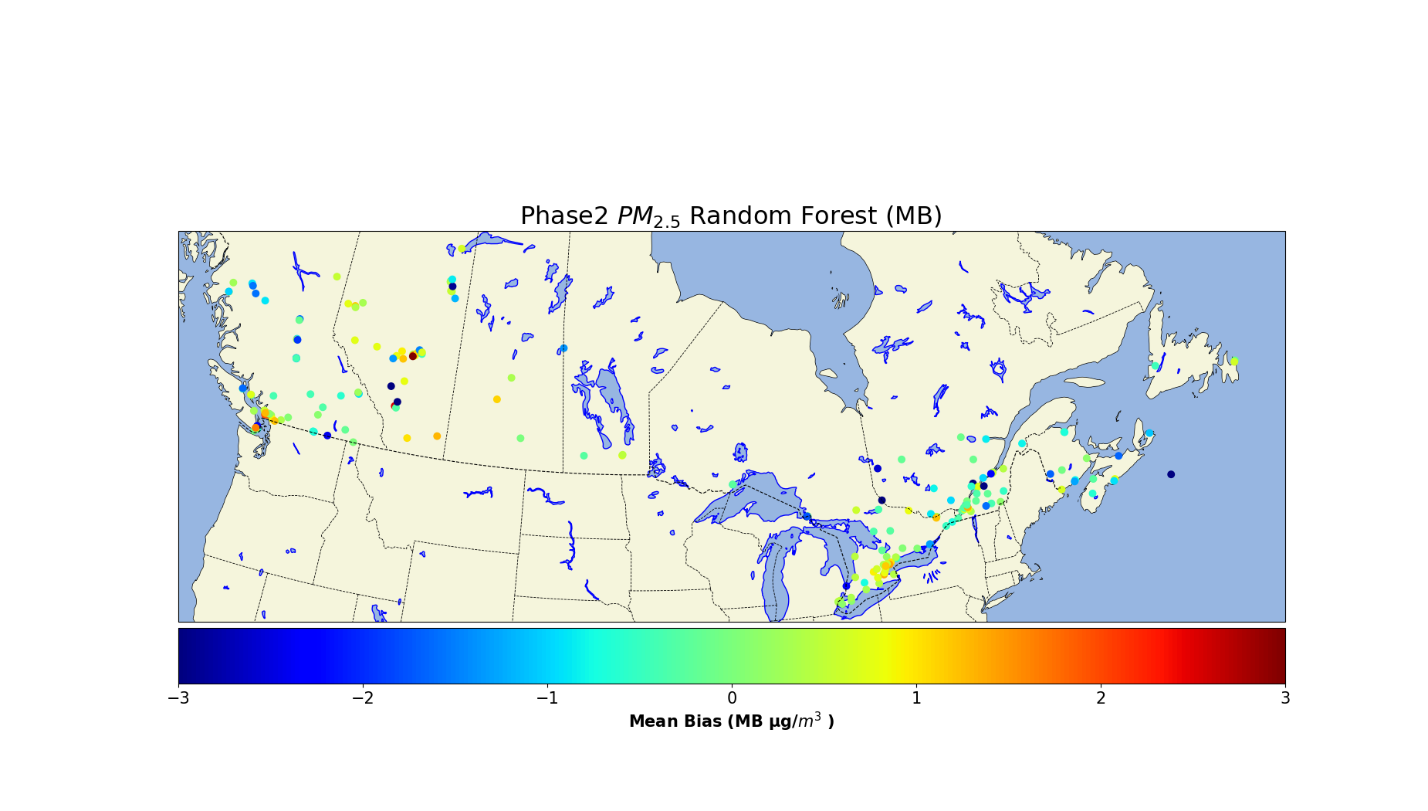


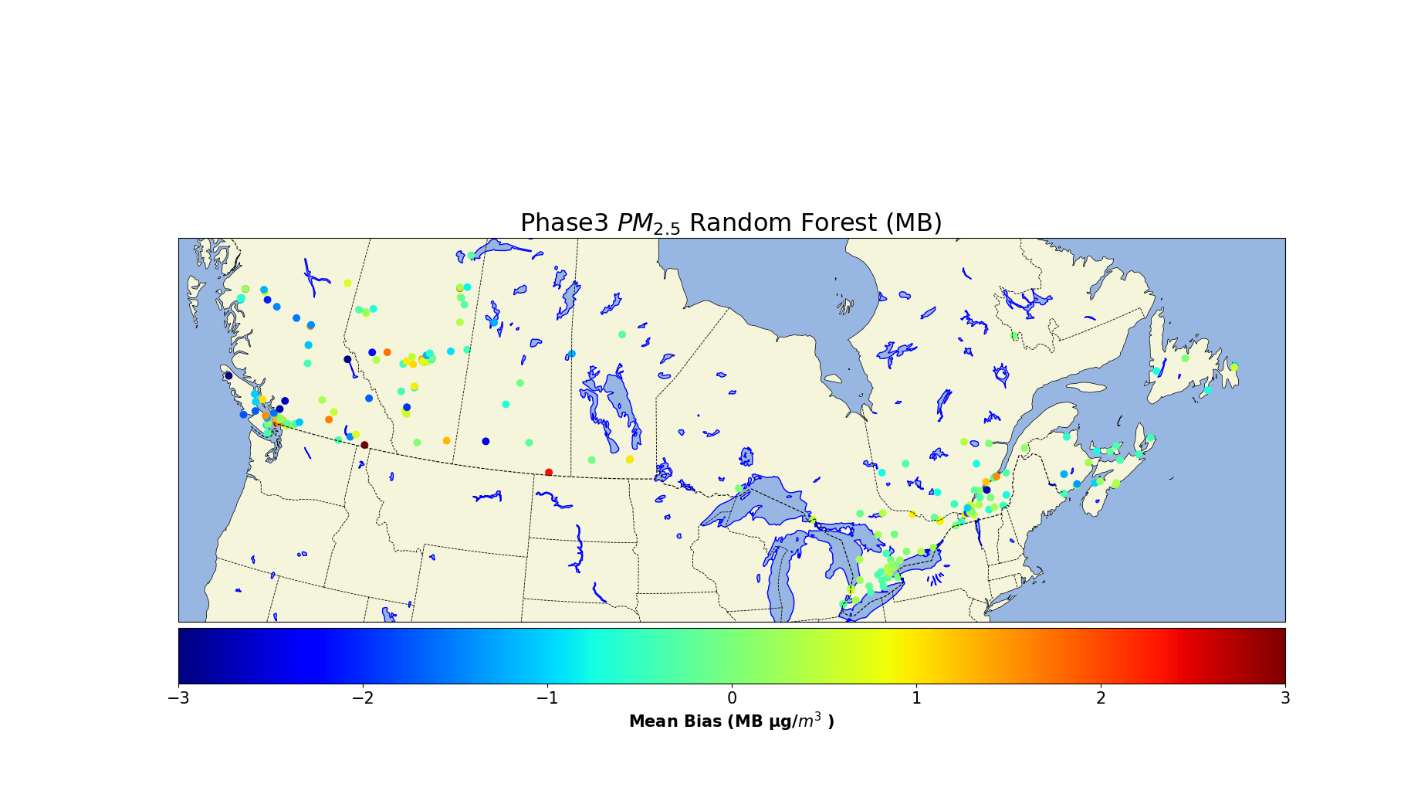


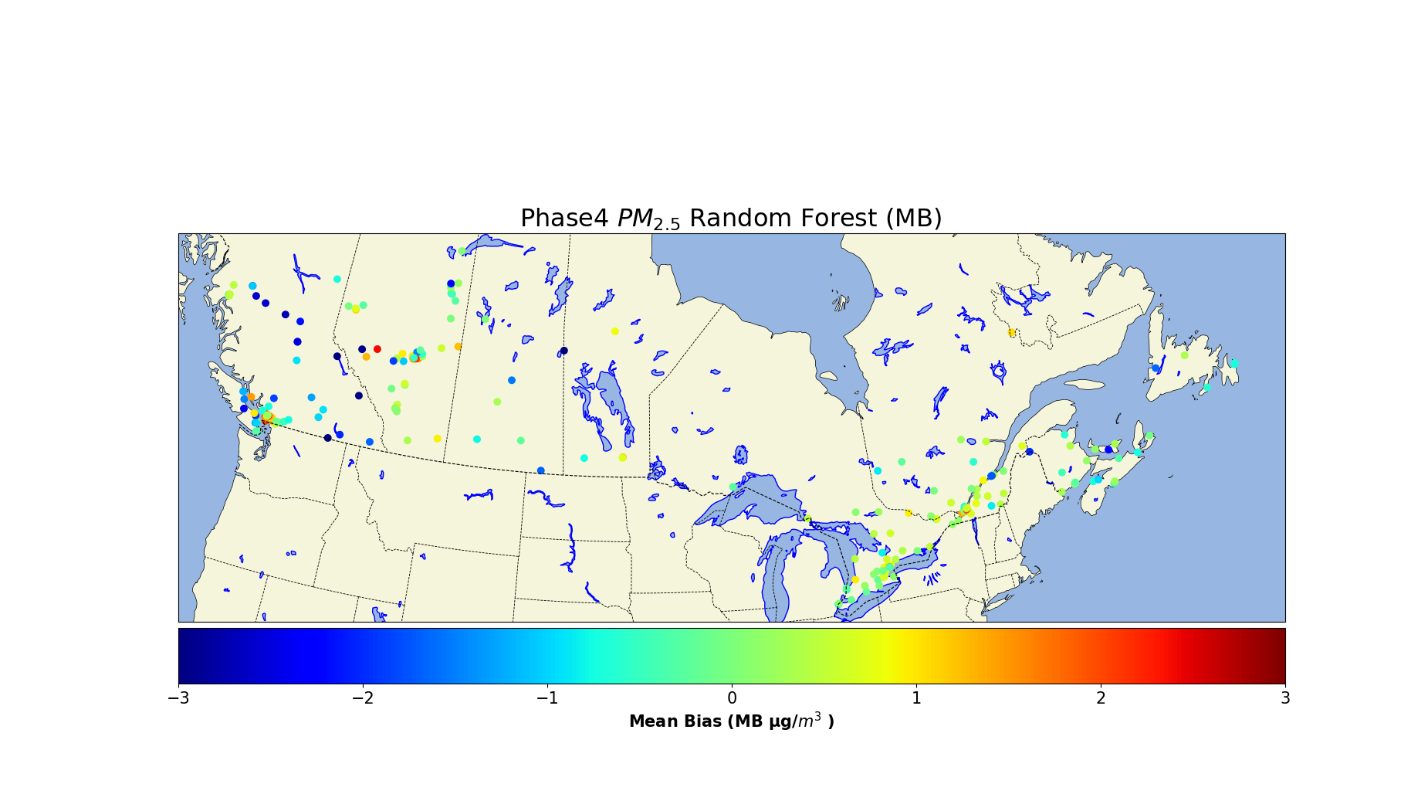


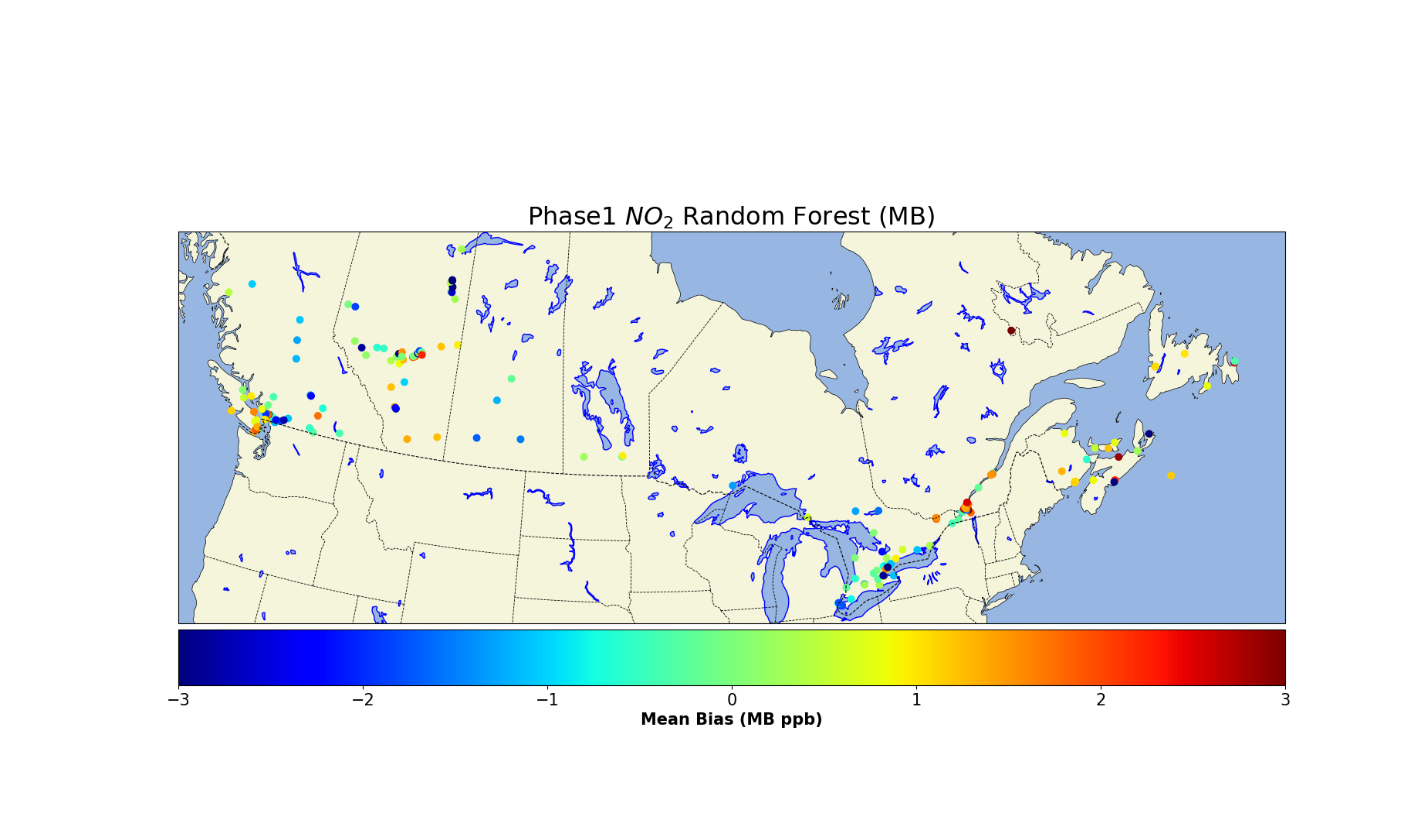


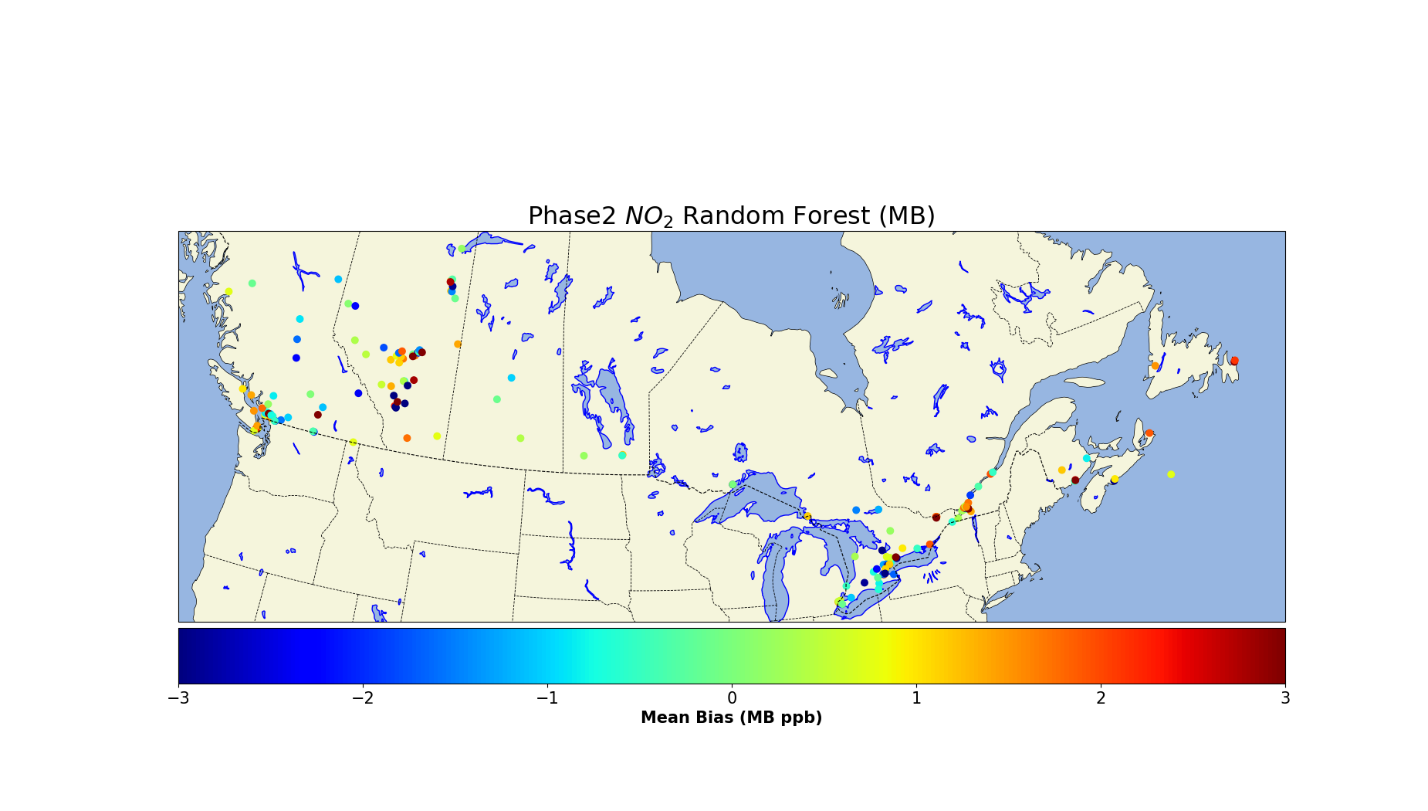


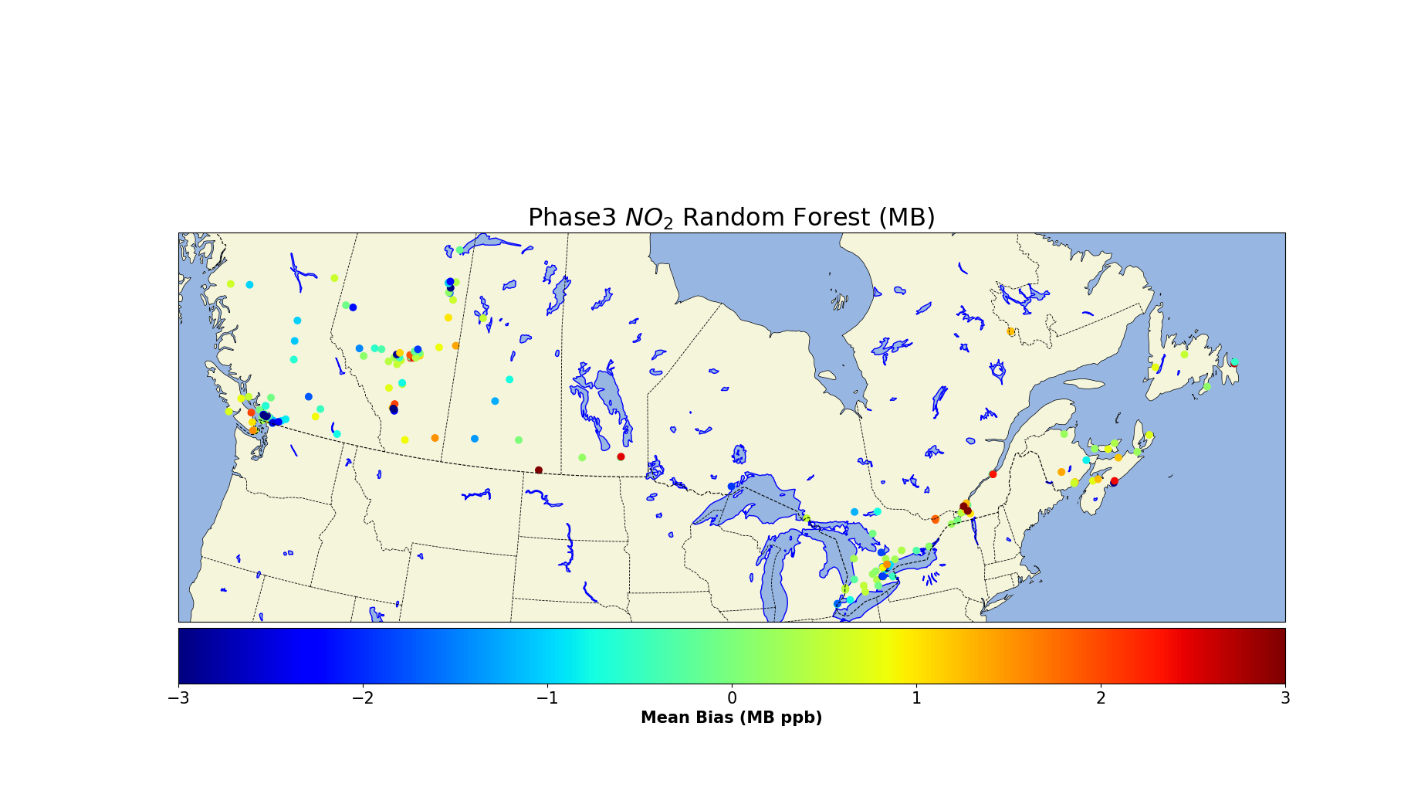


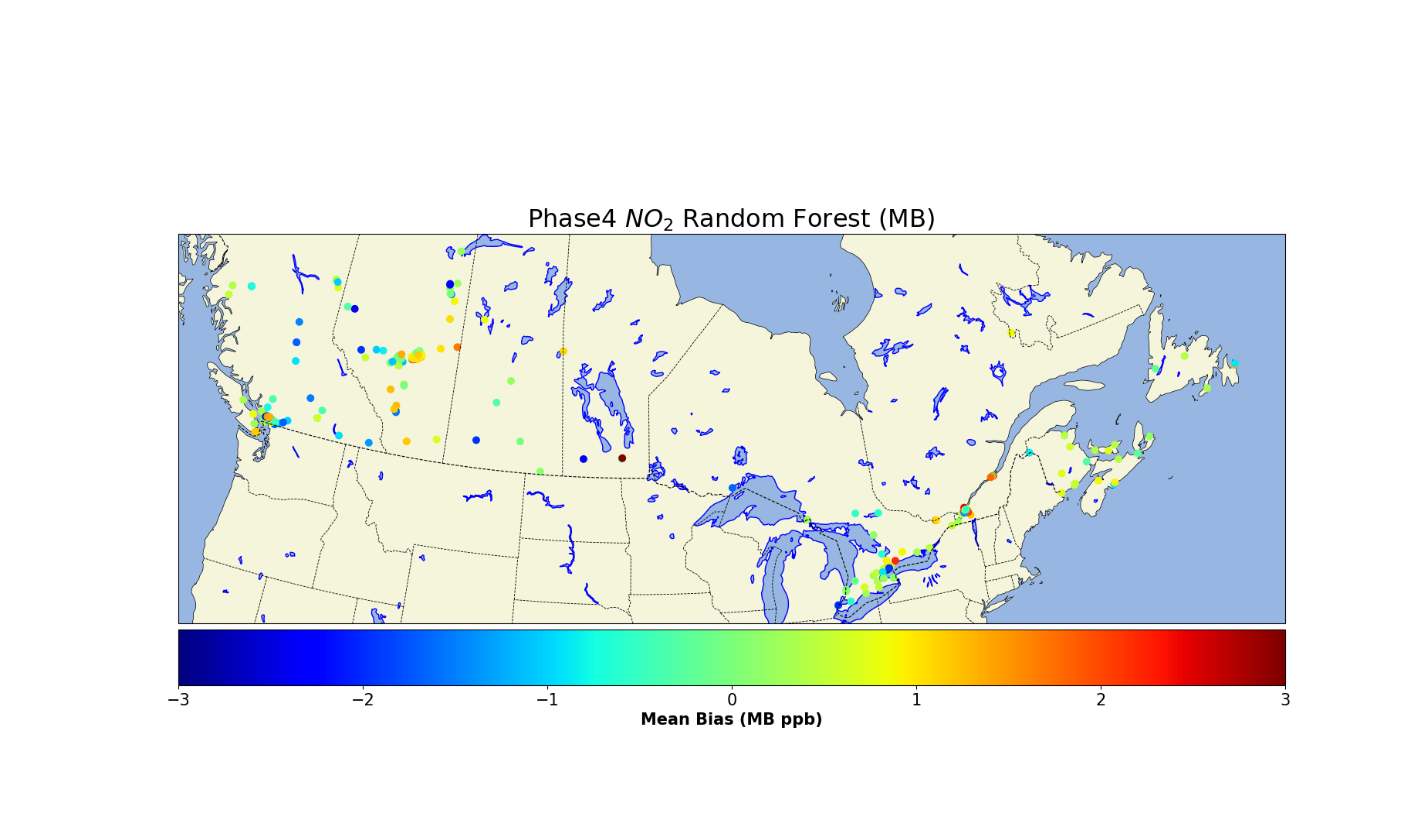


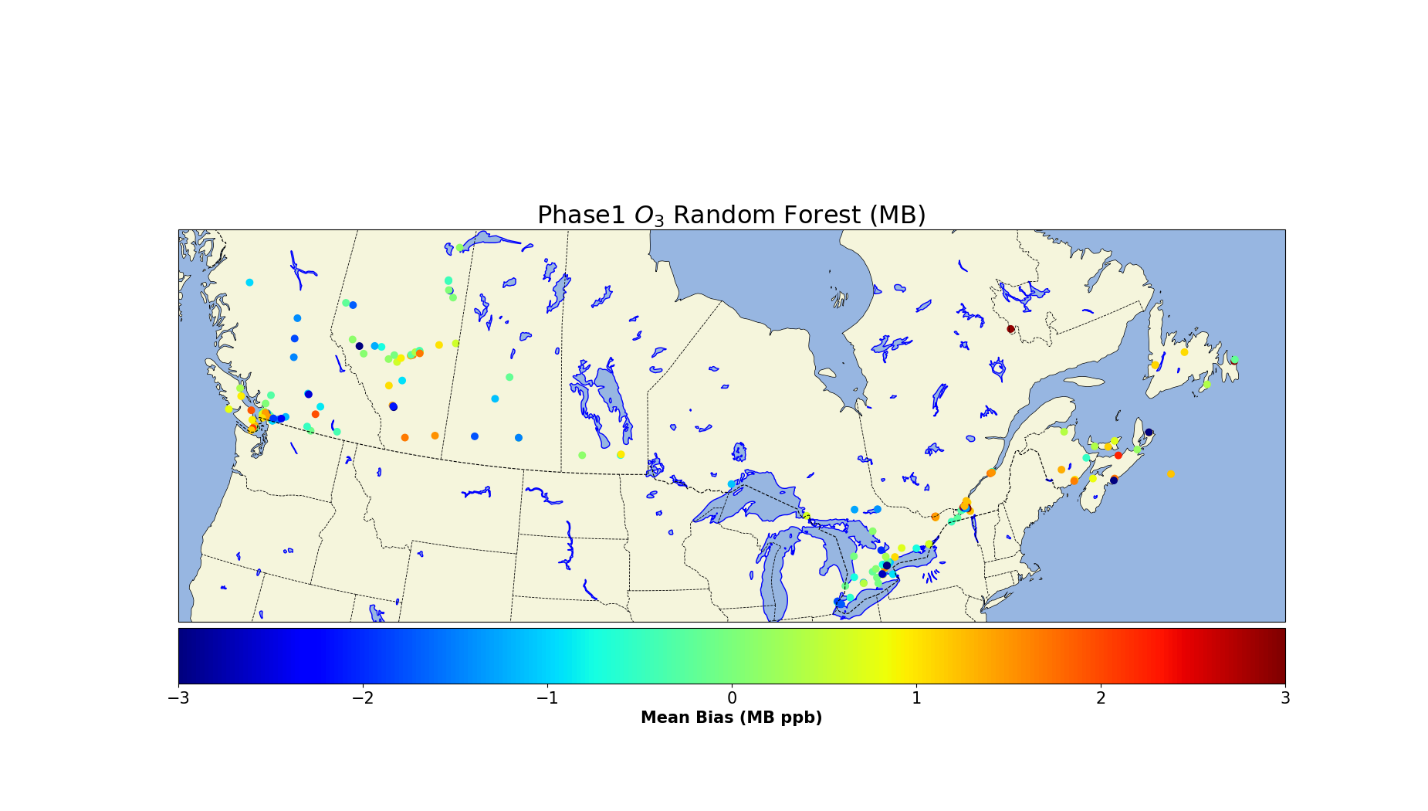


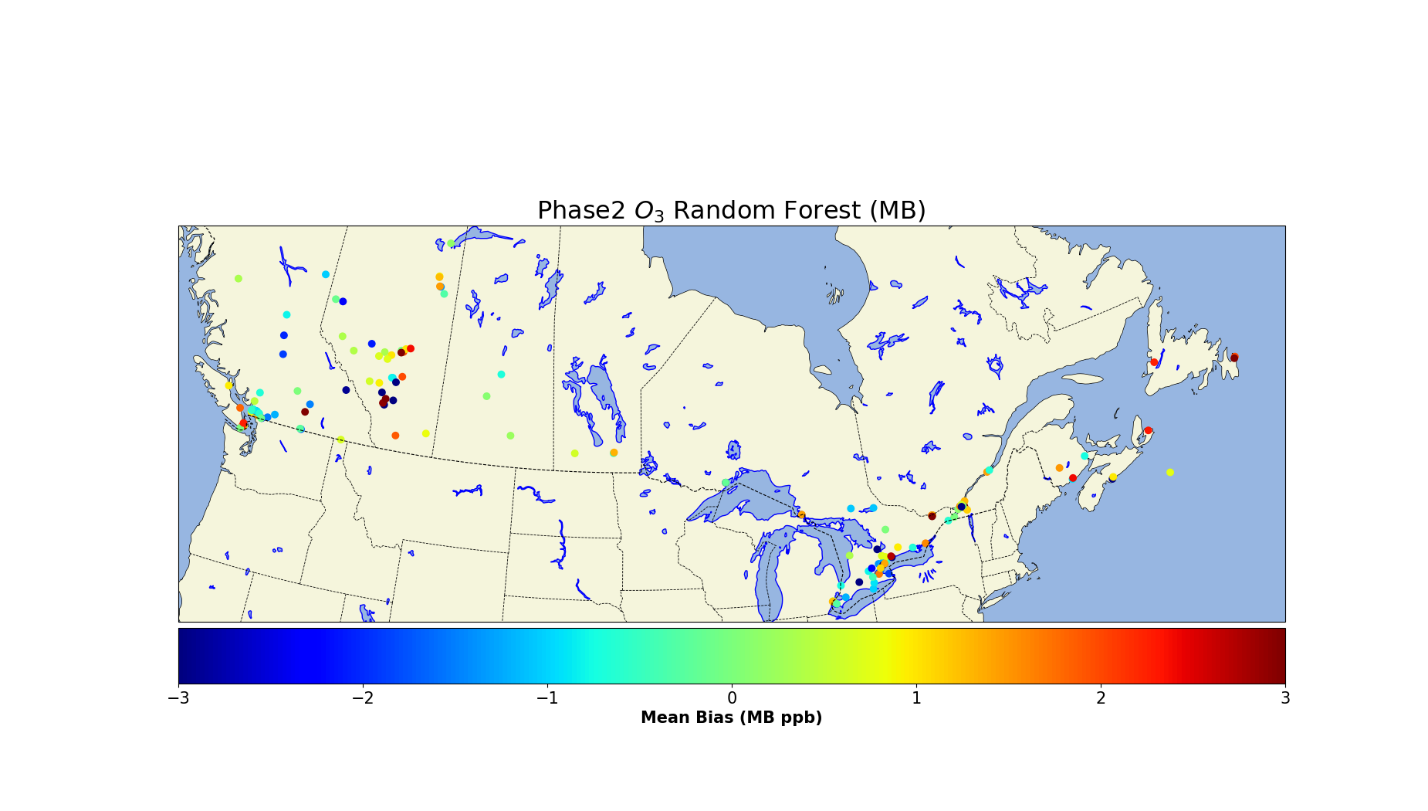


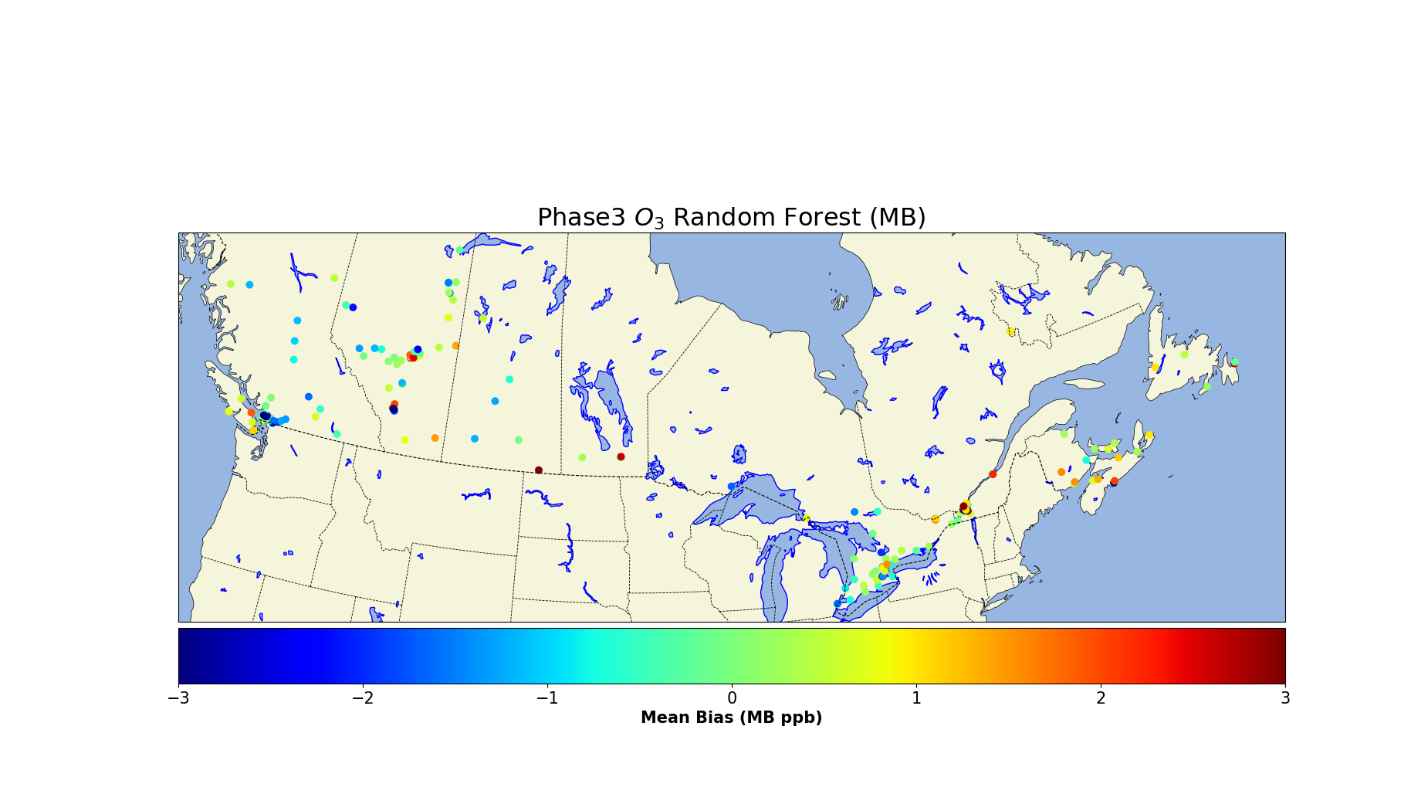


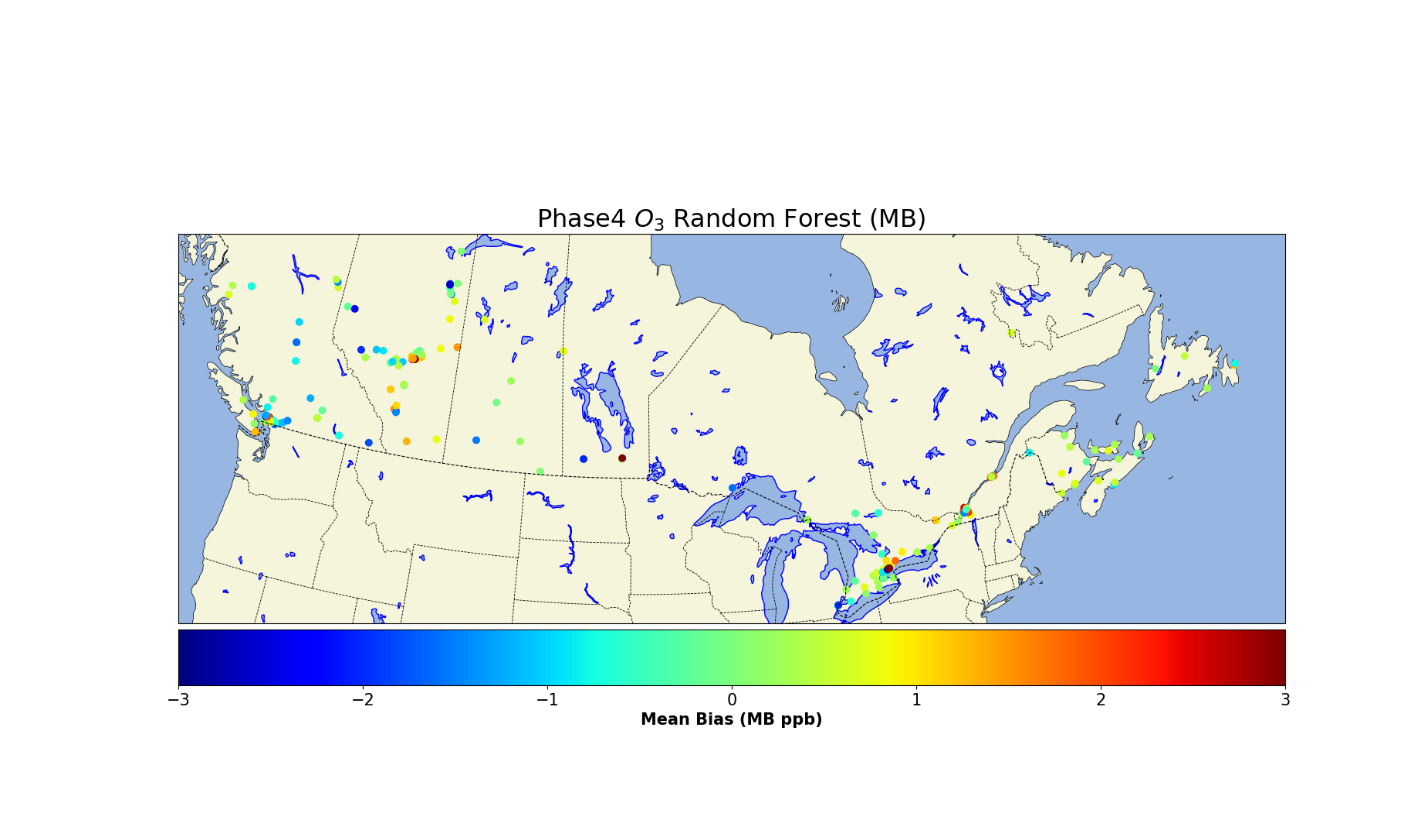


**Figure A2 panel (a): Stations based mean bias phase-wise Random Forest for PM_2.5_, NO_2_, and O_3_.**


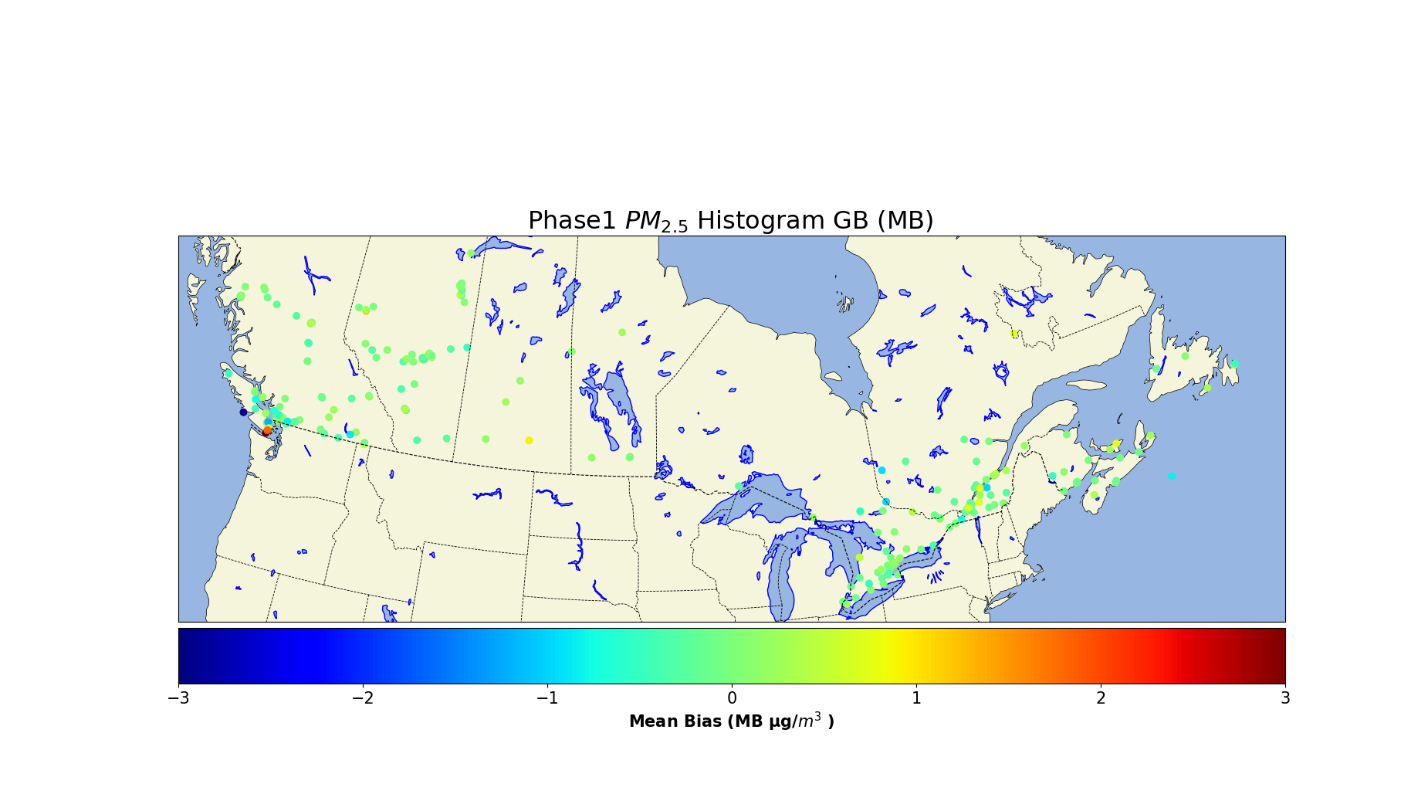


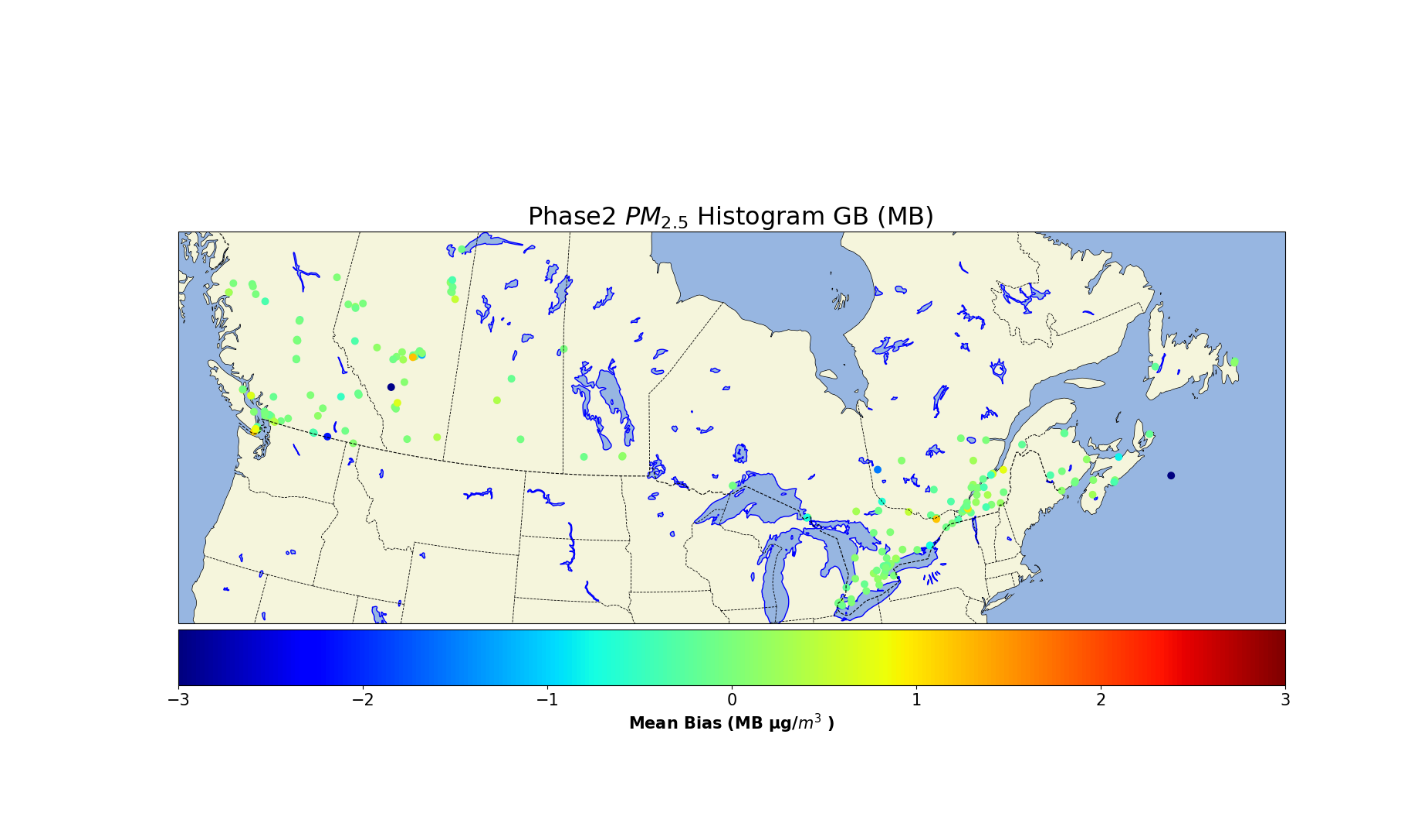


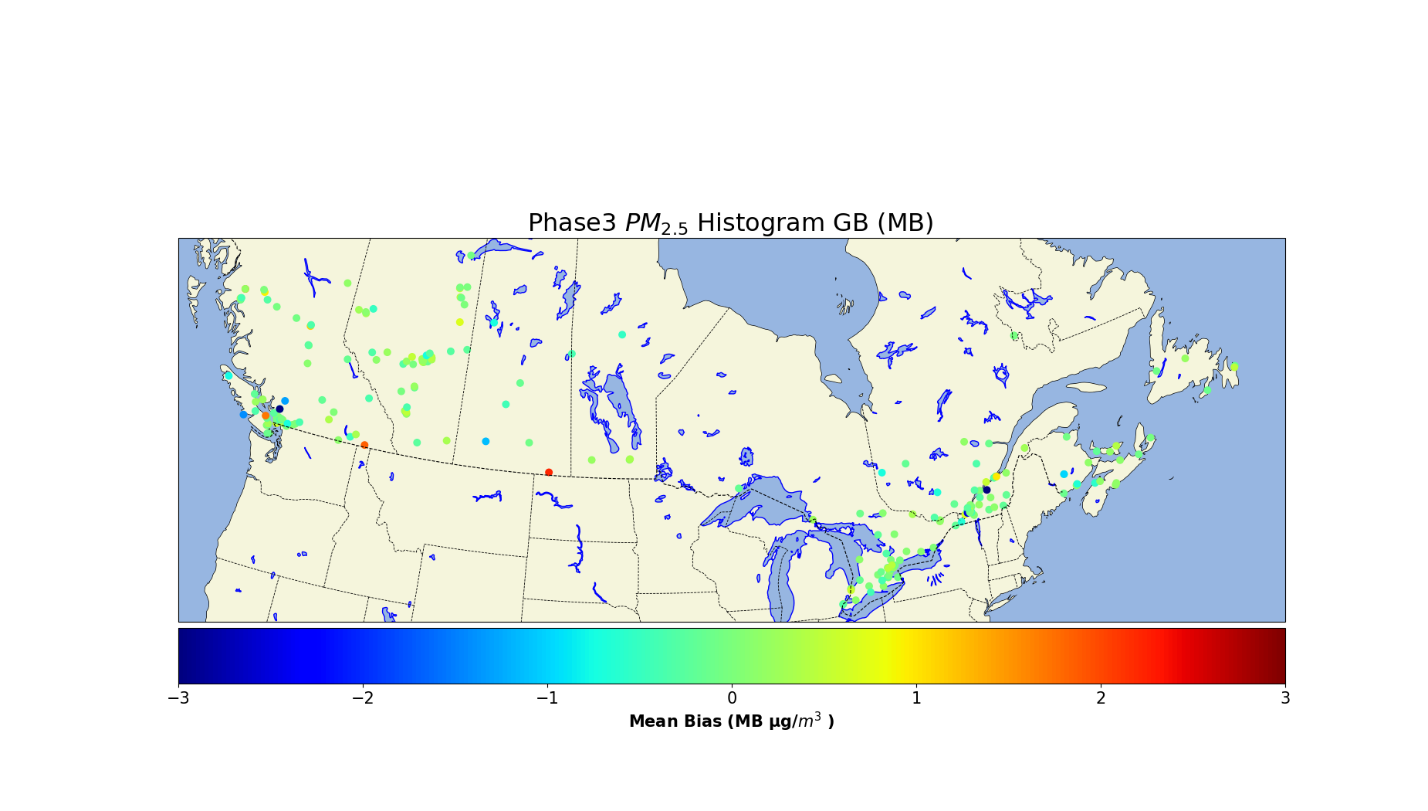


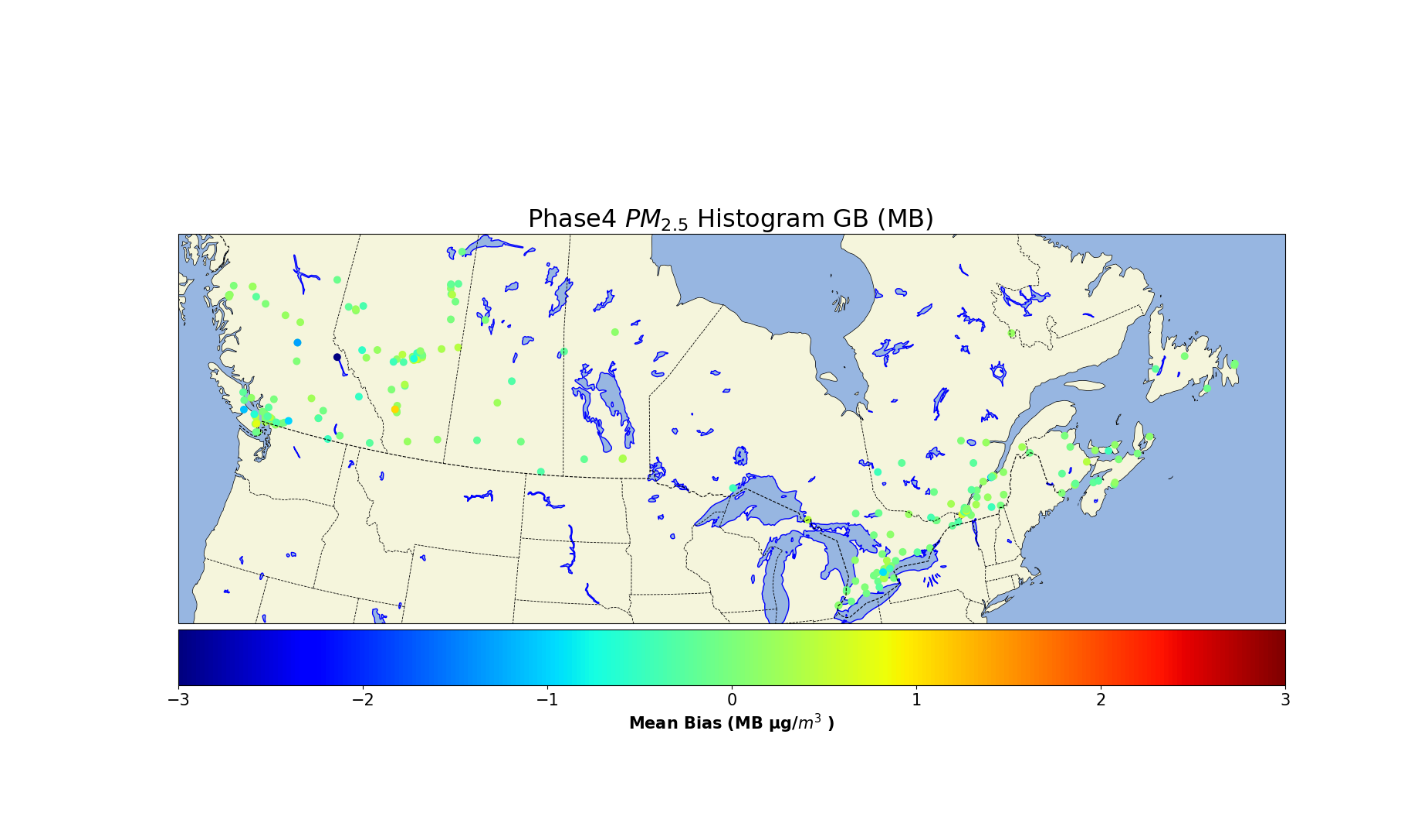


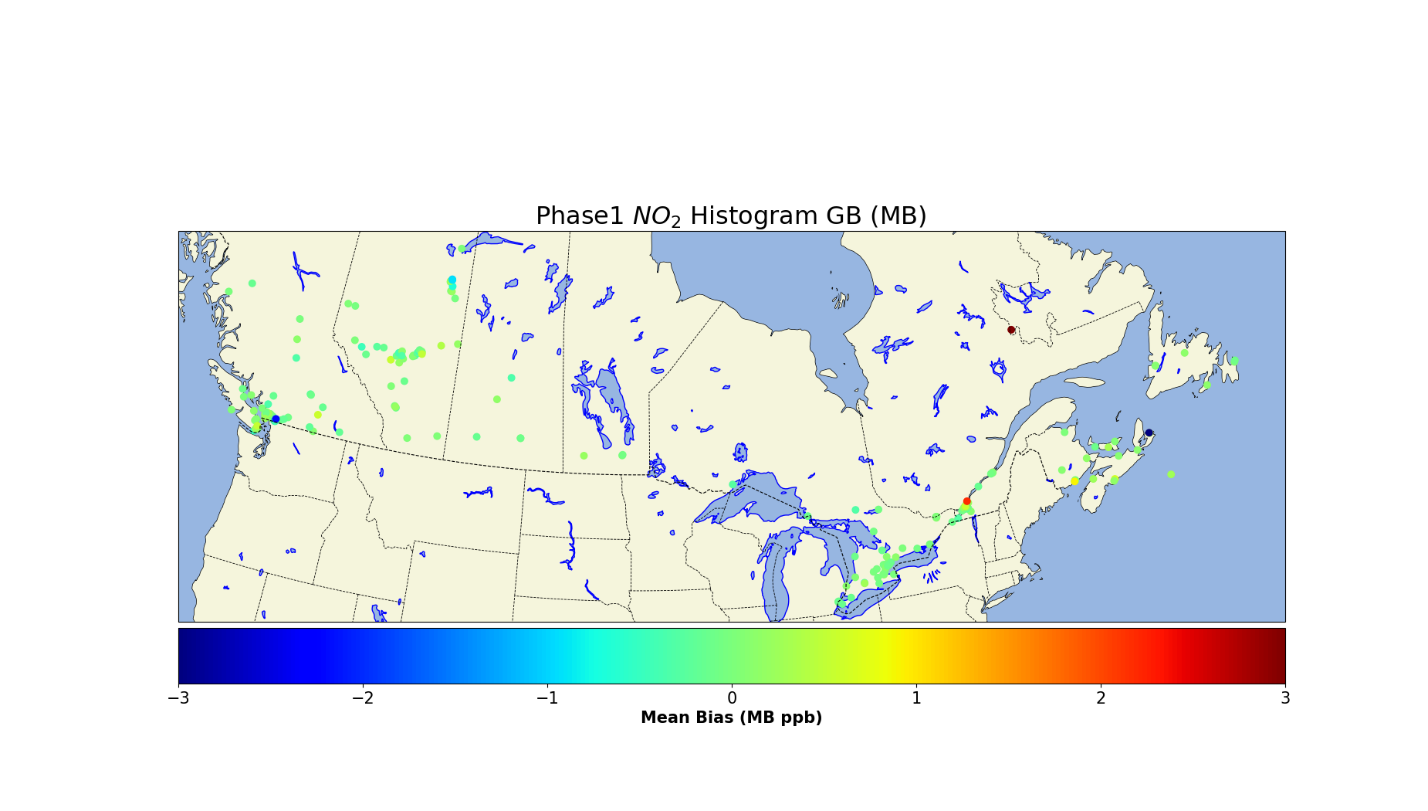


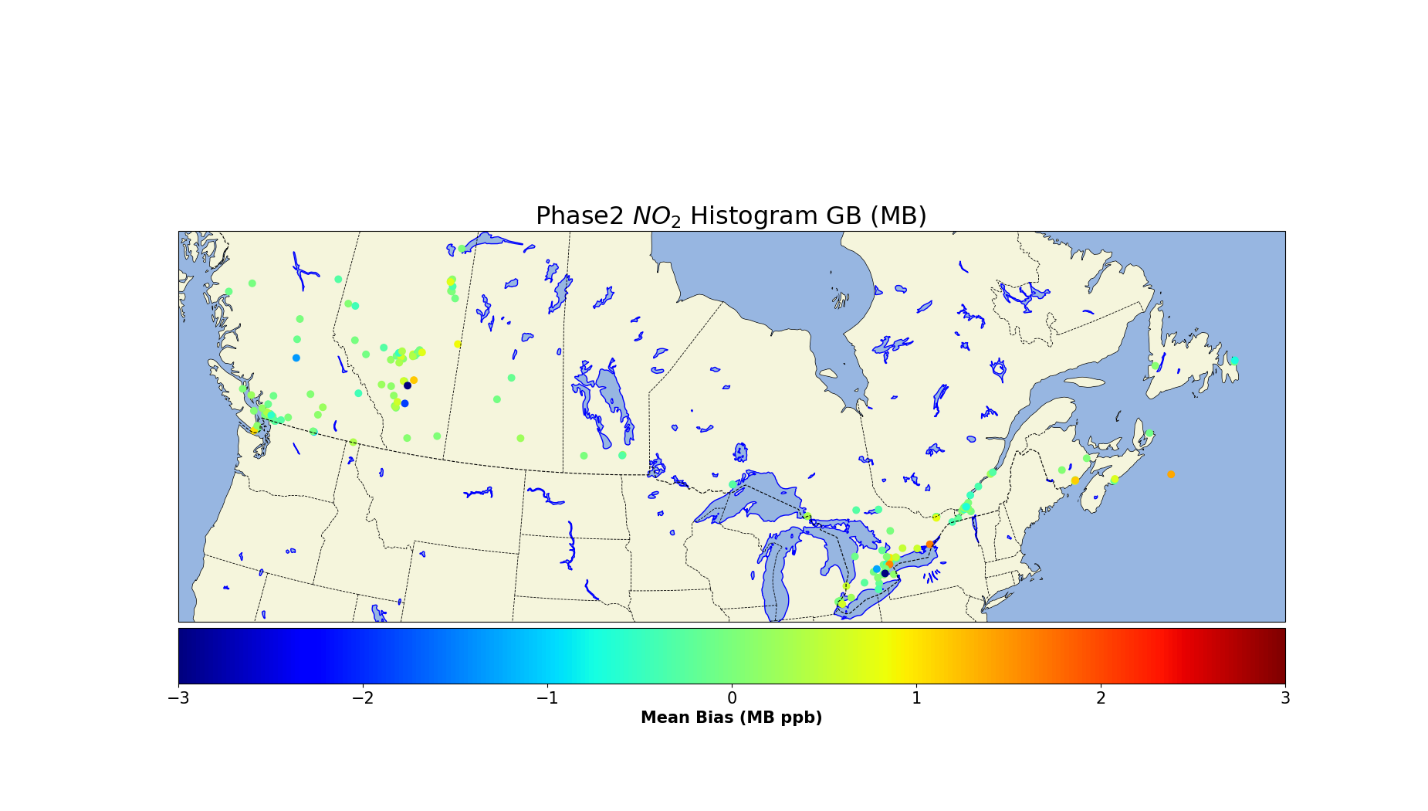


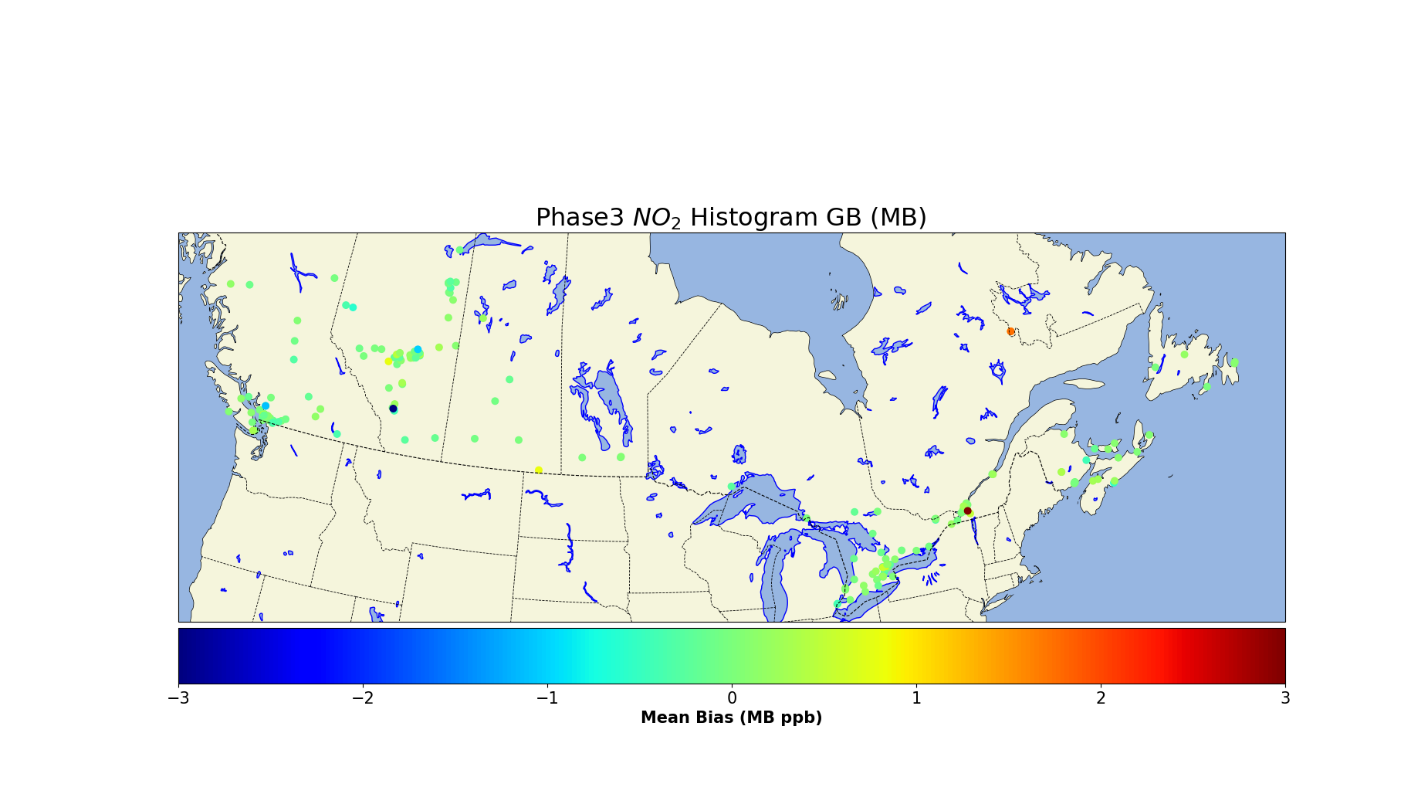


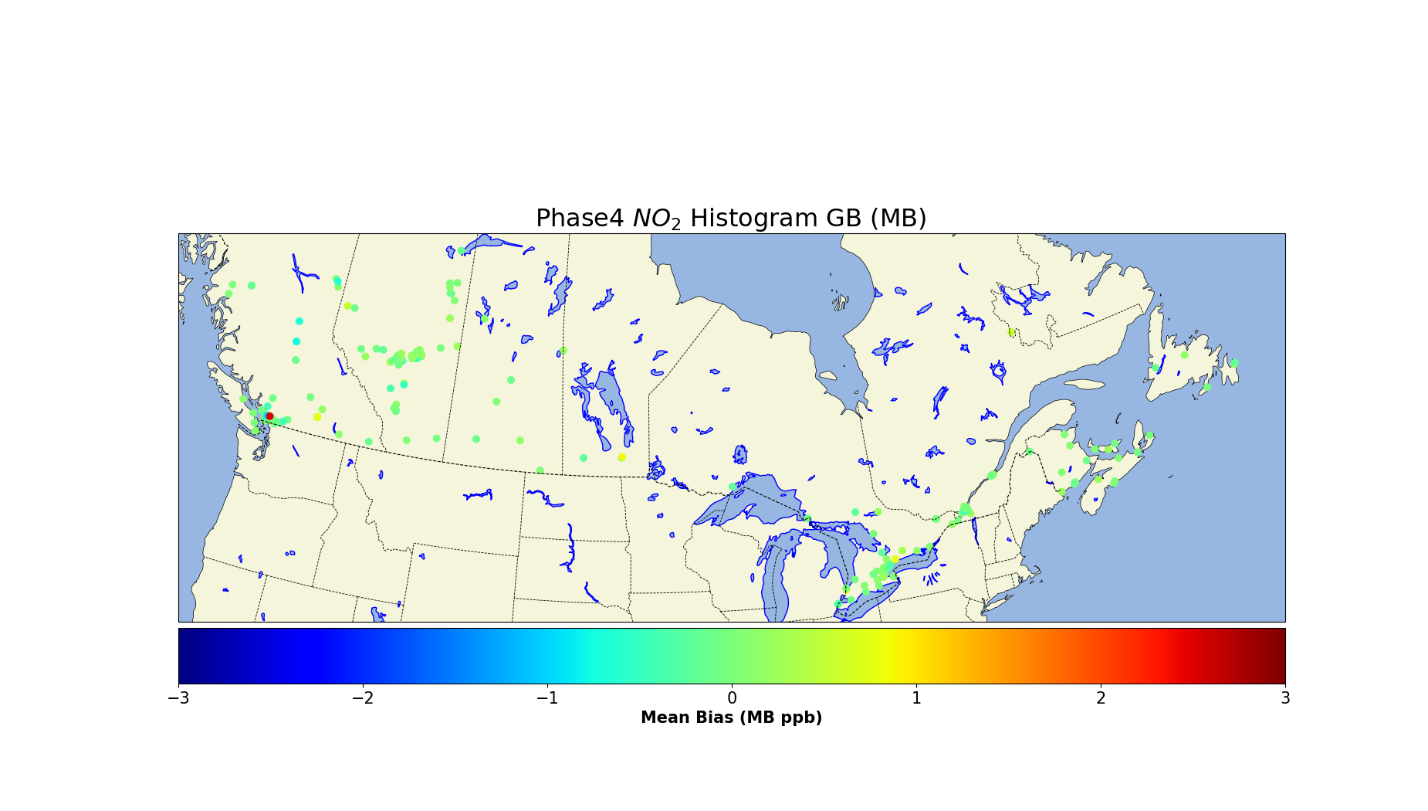


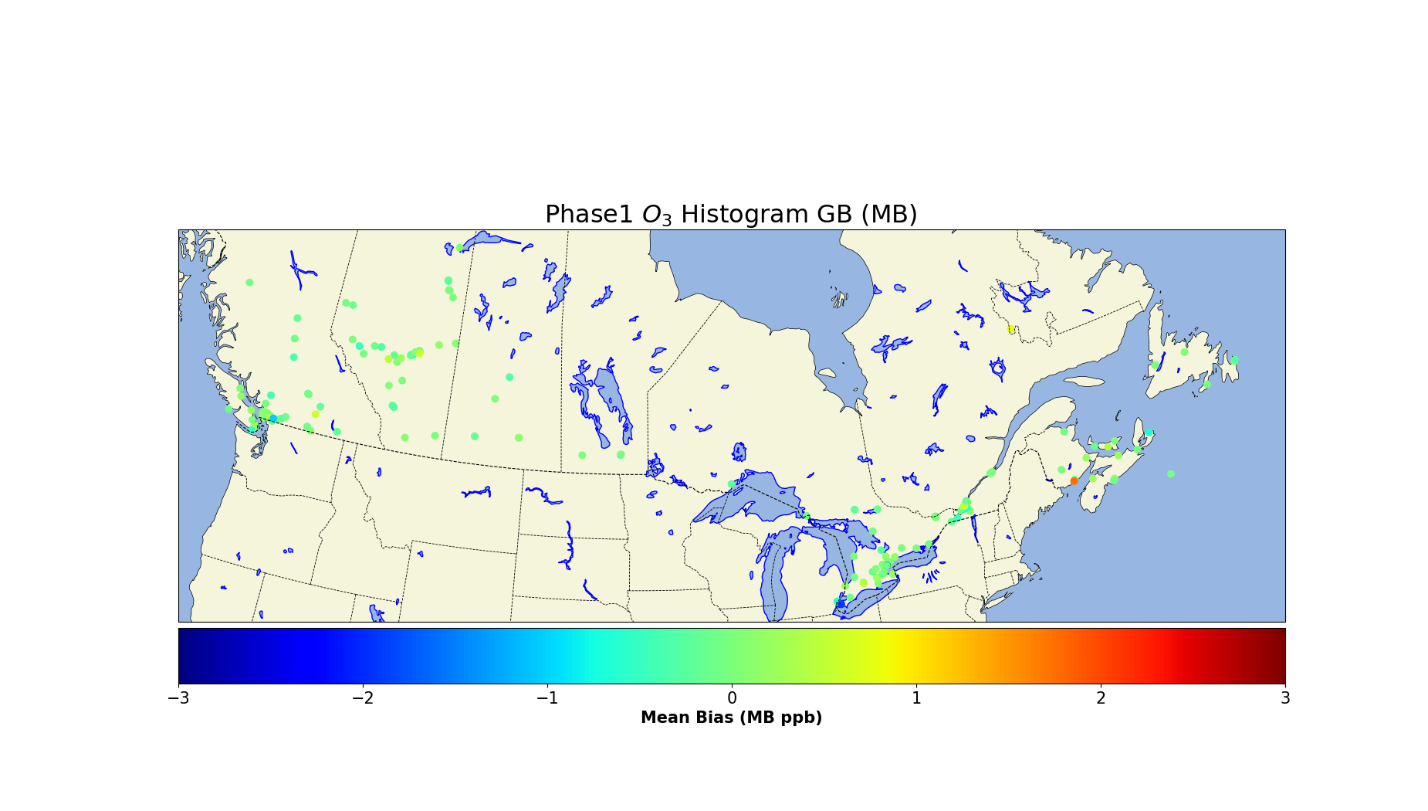


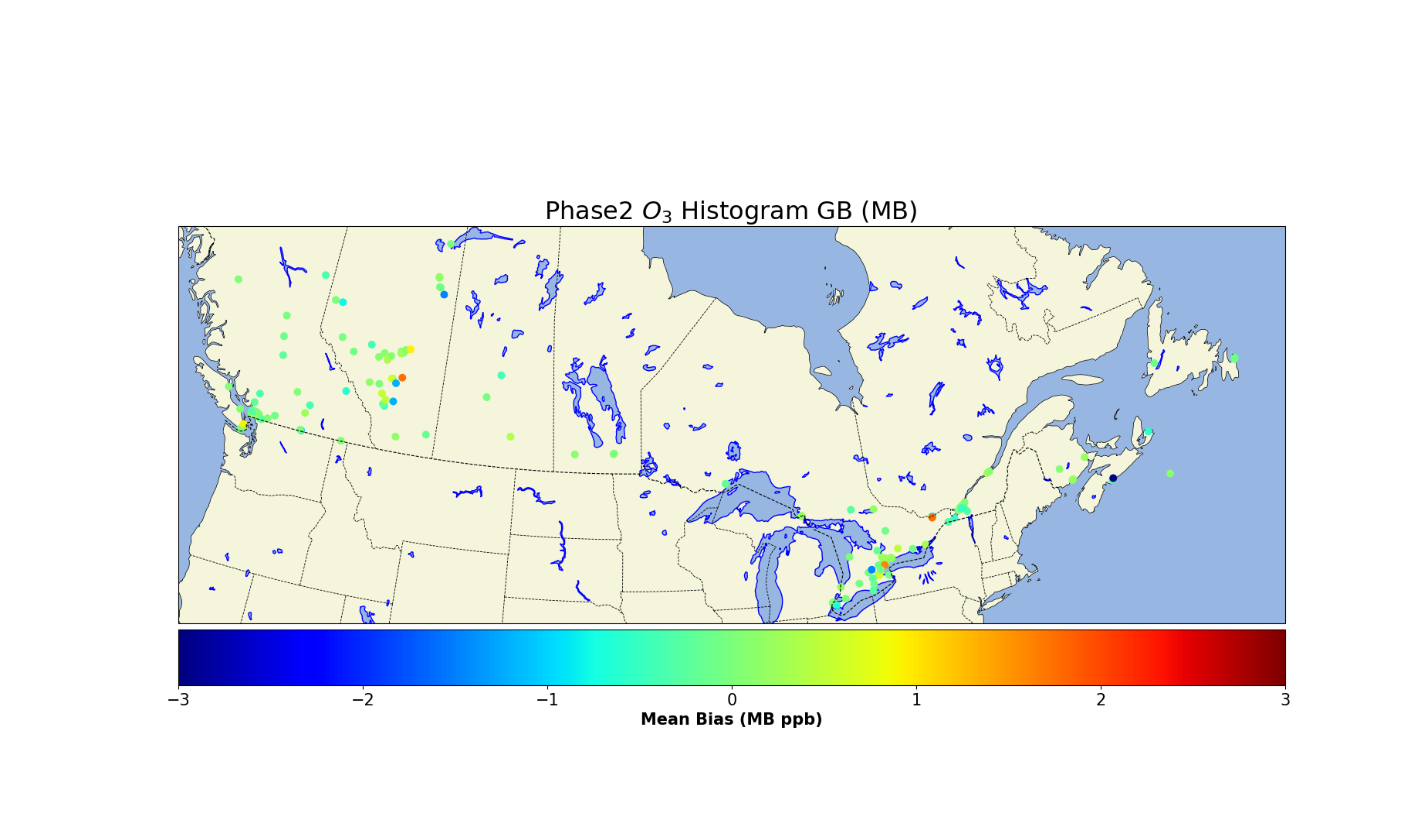


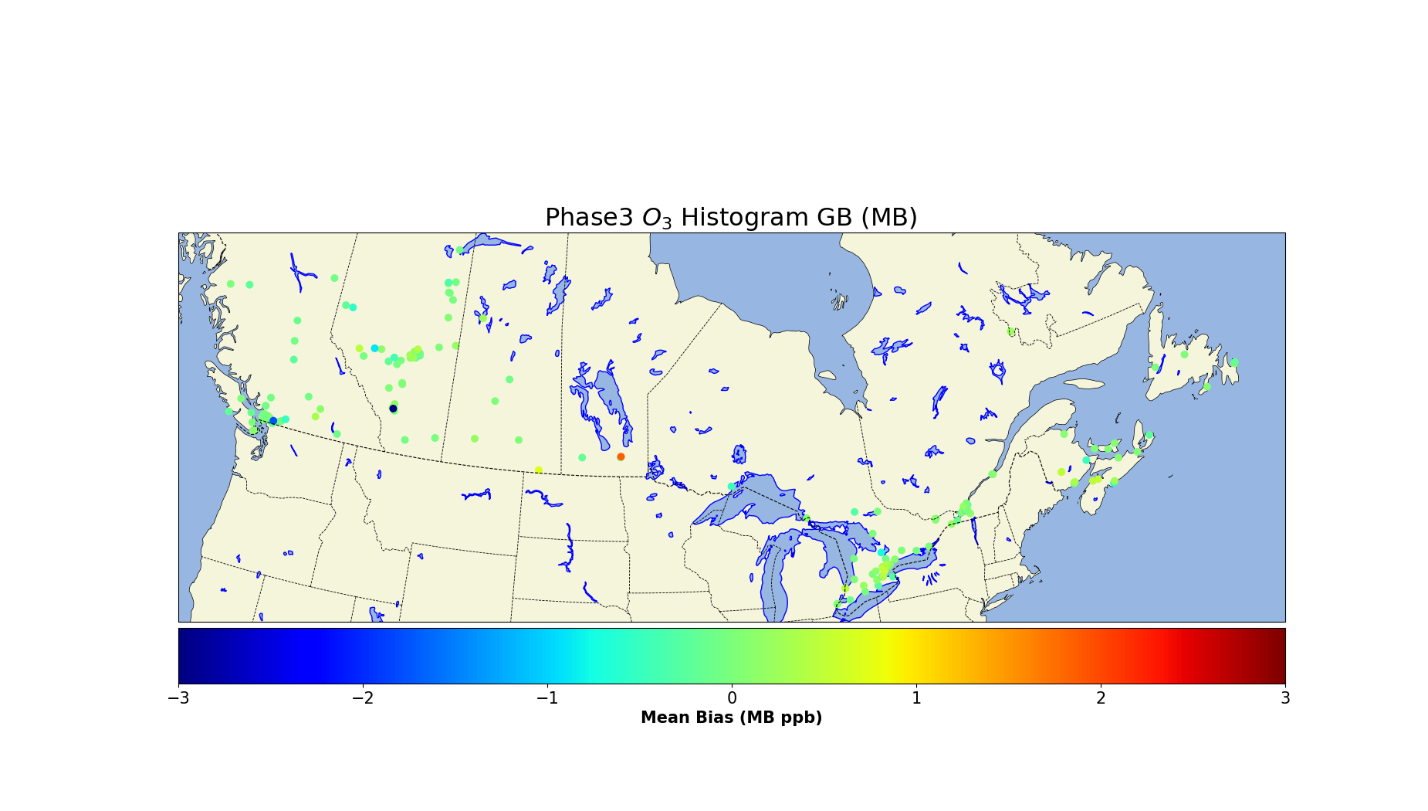


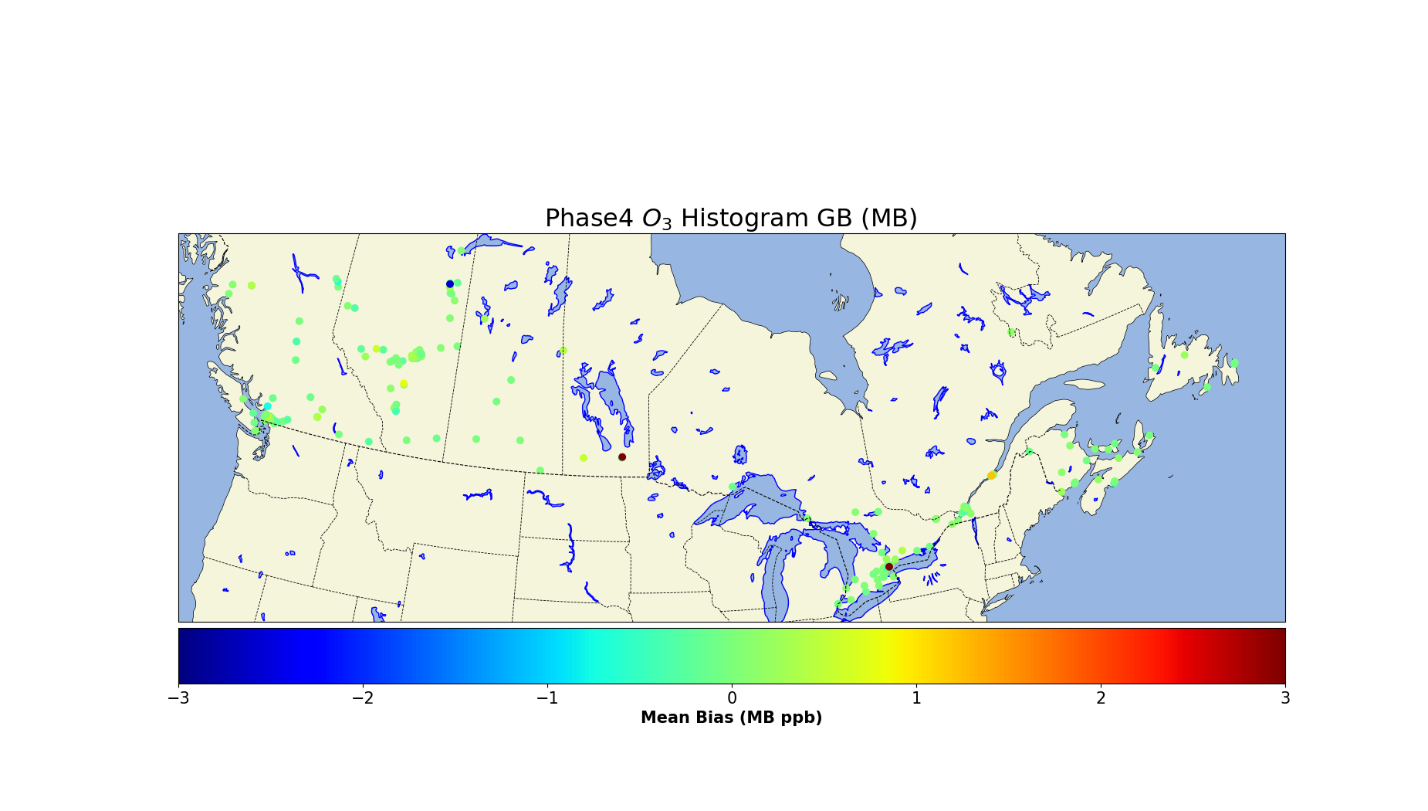


Figure A2 panel (b): Stations based mean bias phase-wise Histogram Gradient Booster for PM_2.5_, NO_2_, and O_3_.


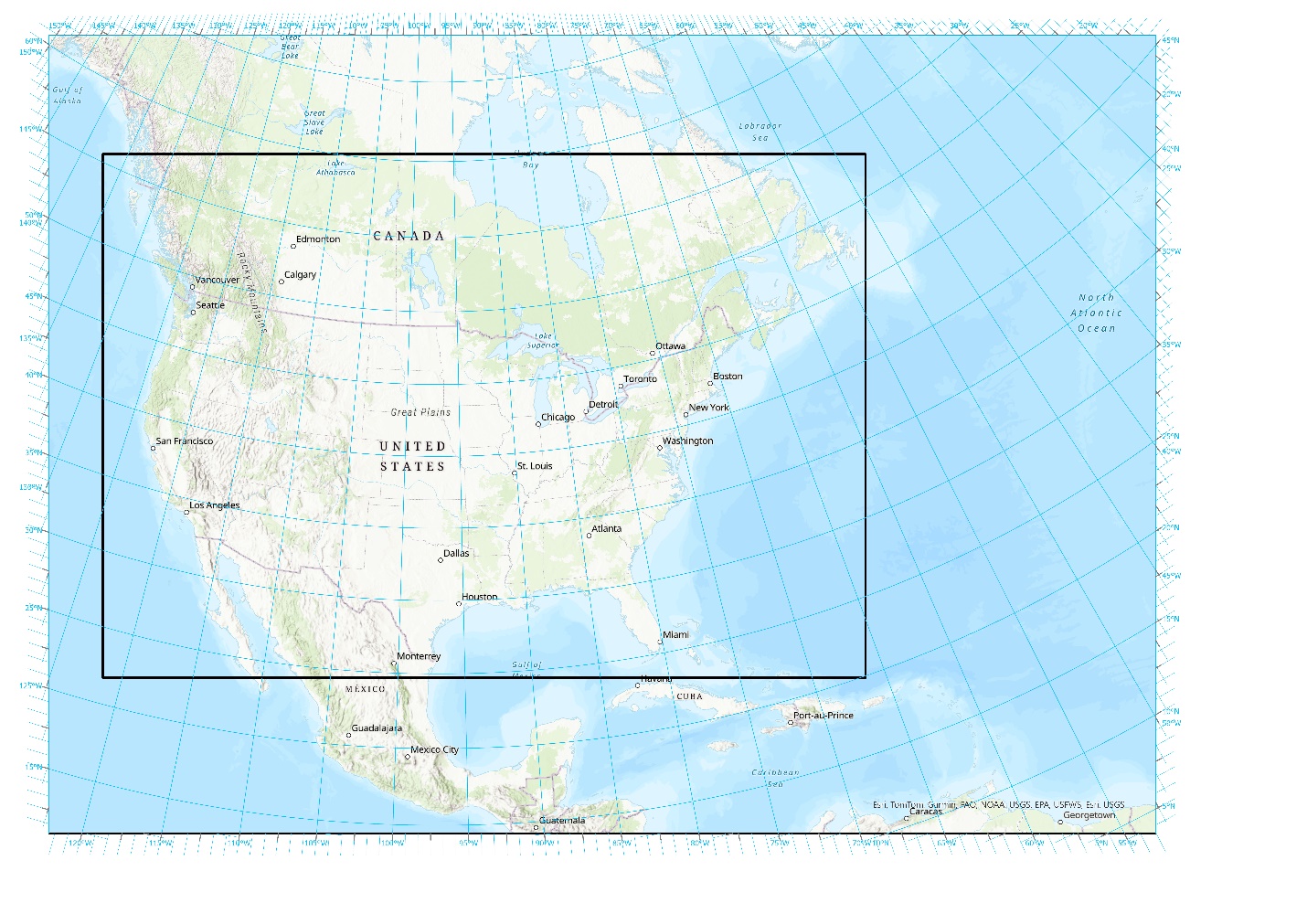


Figure A3: Domain used for CMAQ simulations extends 551 cells east to west, 391 cells north to south, using Lambert Conformal Conic projection.
